# Distinct genomic architectures but the same gene underlie the convergent evolution of a plant supergene

**DOI:** 10.1126/sciadv.aec1996

**Published:** 2026-06-10

**Authors:** Giacomo Potente, Narjes Yousefi, Rimjhim Roy Choudhury, Stefan Grob, Irina A. Gavrilina, Barbara Keller, Emiliano Mora-Carrera, Péter Szövényi, Rebecca L. Stubbs, Hanna Weiss-Schneeweiss, Eva M. Temsch, Gerald M. Schneeweiss, Matthias H. Hoffmann, Giulio Formenti, Ann M. McCartney, Alice Mouton, Henrique G. Leitão, Genevieve Diedericks, Hannes Svardal, Maria Angela Diroma, Chiara Natali, Claudio Ciofi, Étienne Léveillé-Bourret, Elena Conti

**Affiliations:** ^1^ Department of Systematic and Evolutionary Botany, University of Zürich, Zollikerstrasse 107, 8008 Zürich, Switzerland.; ^2^ Zürich-Basel Plant Science Center, ETH-Zürich, Tannenstrasse 1, 8092 Zürich, Switzerland.; ^3^ Department of Evolutionary Biology and Environmental Studies, University of Zürich, Winterthurerstrasse 190, 8057 Zürich, Switzerland.; ^4^ Department of Biology, University of Fribourg, Rue A.-Gockel 3, 1700 Fribourg, Switzerland.; ^5^ Institut de Biologie Moléculaire des Plantes (IBMP) du CNRS, 12 rue du Général Zimmer, 67084 Strasbourg, France.; ^6^ Department of Botany and Biodiversity Research, University of Vienna, Rennweg 14, A-1030 Vienna, Austria.; ^7^ Martin-Luther-Universität Halle-Wittenberg, Botanischer Garten, Am Kirchtor 3, 06108 Halle (Saale), Germany.; ^8^ The Vertebrate Genome Laboratory, The Rockefeller University, 1230 York Ave., New York 10065, NY, USA.; ^9^ Genomics Institute, University of Santa Cruz, 2300 Delaware Ave., Santa Cruz, CA 95060, USA.; ^10^ University of Liege, Arlon Campus environnement, Socio-économie, Environnement et Développement (SEED), Av. de Longwy 185, 6700 Arlon, Belgium.; ^11^ Department of Biology, University of Antwerp, Antwerp, Belgium.; ^12^ Naturalis Biodiversity Center, Leiden, Netherlands.; ^13^ Department of Biology, University of Florence, 50019 Sesto Fiorentino (FI), Italy.; ^14^ Institut de Recherche en Biologie Végétale (IRBV) and Département de Sciences Biologiques, Université de Montréal, Montréal, Québec, Canada.

## Abstract

Evolution reflects a balance between innovation and constraint, often repurposing existing components in new contexts. Convergent evolution exemplifies this interplay, with similar traits evolving independently in different species, yet the genomic mechanisms enabling this repeatability remain poorly understood. Here, by analyzing 10 chromosome-scale genome assemblies, including seven newly generated, we found that the *S*-locus supergene (a cluster of tightly linked genes controlling a floral dimorphism called distyly) arose independently multiple times within the primrose family, Darwin’s iconic system for studying distyly. In each case, the same gene was independently duplicated and co-opted. However, the resulting genomic architectures differed, ranging from hemizygous (present on one chromosome copy) to heterozygous (on both copies), challenging the prevailing view that hemizygosity is intrinsic to *S*-loci and suggesting alternative evolutionary routes to distyly supergene formation. By uncovering multiple mechanisms for supergene origins, our work shows how convergent evolution can produce similar phenotypes by reusing the same genetic building blocks while exploring distinct genomic configurations.

## Teaser

High-quality genome assemblies from multiple plant species reveal different paths of evolution in supergenes.

## INTRODUCTION

Convergent evolution, defined as the independent acquisition of similar traits in distinct lineages (*1*), is a central topic in evolutionary biology, for it showcases the power of natural selection in driving the repeated emergence of similar adaptive traits under similar selective pressures (*2*). Understanding the genetic basis of convergence is a central question in evolutionary biology, and, although recent advances in genomics have made it more accessible, these investigations remain challenging when convergent traits are controlled by multiple, often dispersed genes across the genome. Conversely, supergenes, i.e., nonrecombining genomic regions containing genes that jointly control a set of coadapted polymorphic traits (*3*–*5*), offer a twofold advantage for studying convergent evolution: As simple Mendelian loci, they can be readily associated with phenotypic traits and enable exploring the role of genomic architecture in adaptation and convergence (*6*). When candidate genes of convergent traits are known, it is possible to discern whether phenotypic convergence arises from mutations in the same genes, mutations in different genes with a shared biochemical pathway, or the involvement of entirely different genes producing the same phenotypic outcome (*7*, *8*). In addition, while extensive research has focused on identifying genes responsible for phenotypic convergence, the influence of genomic architecture on convergent evolution remains underexplored.

**Fig. 1.**
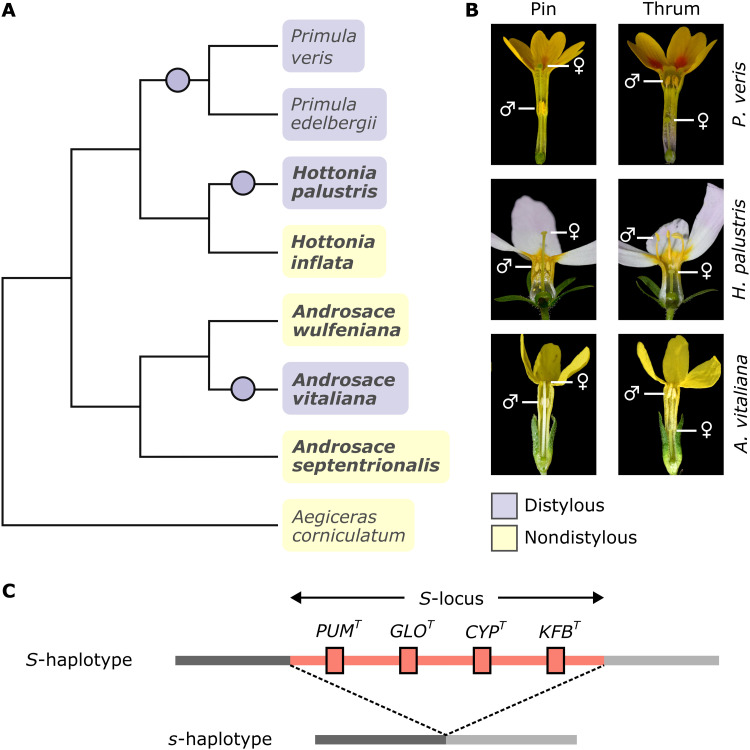
Multiple origins of distyly in Primulaceae. (**A**) Cladogram describing relationships among the eight Primulaceae species whose chromosome-scale assemblies were used in this study and showing the three origins of distyly (purple circles) previously inferred in a phylogenetic study of 265 Primulaceae species (*29*); *Primula* comprises 526 species, of which 82% distylous; *Hottonia* comprises only two species, of which only *H. palustris* is distylous; *Androsace* comprises 175 species, of which only *A. vitaliana* is distylous. *A. corniculatum* was used as nondistylous outgroup. Distylous and nondistylous species are highlighted in purple and yellow, respectively; species whose genome assemblies are presented here for the first time are boldfaced. Haplotype-phased assemblies were generated for the distylous *H. palustris* and *A. vitaliana*. (**B**) Flowers of three distylous species: pins (left) have stigma above anthers, while thrums (right) have anthers above stigma. Photo credit: A. Bernhard. (**C**) Schematic representation of the *S*-locus in *Primula*, comprising four core genes present only in the dominant *S*-haplotype and absent from the recessive *s*-haplotype. In all distylous species studied so far, the *S*-locus is hemizygous in thrums (*S*/*s*) and absent in pins (*s*/*s*).

One of the best studied supergenes is the *S*-locus controlling distyly, a floral dimorphism characterized by the coexistence within the same species of two floral morphs, called “pin” and “thrum,” with reciprocally positioned sexual organs, promoting cross-pollination (Fig. 1B) (*9*–*11*). Having evolved independently multiple times across angiosperms (*12*) distyly represents a prime example of phenotypic convergence. Recently, *S*-loci were characterized in several species from distantly related angiosperm taxa, revealing that in at least five cases, the key gene determining short styles in thrums acts by inactivating brassinosteroids, although the specific gene may differ (*13*–*18*).

Besides this functional convergence, the known *S*-loci are also characterized by convergent genomic architecture, with the *S*-locus consistently being hemizygous in thrums (*S*/*s*) and absent in pins (*s*/*s*) (Fig. 1C) (*13*, *16*–*25*). *S*-loci share several key features with sex-determining regions (e.g., Y chromosomes), including hemizygosity, reduced local recombination, and multiple coadapted alleles, making their evolutionary dynamics broadly comparable (*26*).

The consistent observation of hemizygosity across distylous species led to the hypothesis that *S*-loci evolved by gene duplications and translocations to the same (*S*) haplotype, thus establishing hemizygosity from the onset. This hypothesis received support from several distylous systems (*16*, *17*, *23*–*25*, *27*, *28*). Alternatively, hemizygosity could arise secondarily through gene losses from the recessive (*s*) haplotype, but such a case has not been reported to date. Thus, whether hemizygosity is a universal feature of distyly supergenes and whether it is established from the onset or through subsequent gene losses remain unclear.

In Primulaceae, a plant family where distyly has been studied since Darwin (*9*), a phylogenetic analysis of 265 distylous and nondistylous species inferred three independent origins of distyly: in the ancestor of *Primula*, in *Hottonia palustris*, and in *Androsace vitaliana* (Fig. 1A) (*29*). In *Primula*, in which distyly is accompanied by a self- and intramorph-incompatibility system, the *S*-locus contains four core genes (*CYP^T^*, *GLO^T^*, *KFB^T^*, and *PUM^T^*; Fig. 1C) (*13*, *27*, *30*), two of which have been functionally characterized: in thrums, *CYP^T^* determines short style (*31*) and female incompatibility (*32*) by inactivating brassinosteroids, while *GLO^T^* elevates anthers (*28*). However, the *S*-loci of *H. palustris* and *A. vitaliana* remain unknown.

Here, we analyze ten chromosome-scale genome assemblies from eight Primulaceae species, seven of which were newly generated, to ask the following questions: (i) Since distyly is inferred to have evolved multiple times within Primulaceae (*29*), did the *S*-locus also evolve repeatedly, or did it originate once deep in the phylogeny, followed by independent losses or modifications in nondistylous lineages? (ii) Do the same genes and genomic architectures underpin distyly in Primulaceae (e.g., is the *S*-locus hemizygous in thrums in all species)? (iii) Do supergenes evolve via colocalization of already functionally interacting genes or via the acquisition of new functions (neofunctionalization) by genes that are already colocalized? (iv) Which evolutionary processes cause the expansion of suppressed recombination? This study provides insights into the relationship between phenotypic and genotypic convergence and deepens our understanding of supergene evolution.

## RESULTS AND DISCUSSION

### Comparative genomic analyses of distylous and nondistylous species reveal a lack of interspecific synteny

To investigate *S*-locus origins in Primulaceae, we assembled a dataset comprising 10 genomes from eight species. Of these, seven assemblies from five Primulaceae species were newly generated using a combination of short- and long-read sequencing and Hi-C scaffolding (tables S1 to S3). The new assemblies comprised the distylous *A. vitaliana* and *H. palustris*, for which we generated phased assemblies with pin and thrum haplotypes assembled separately, and the closely related nondistylous *Hottonia inflata* (sister of *H. palustris*), *Androsace wulfeniana*, and *Androsace septentrionalis*. All genome assemblies presented here were at chromosome scale (Table 1, table S4, and figs. S1 to S9). We identified putative centromeres as regions showing the expected local minimum in gene density and corresponding maximum in transposable element (TE) content, often accompanied by large arrays of tandem repeats (figs. S3 to S9 and table S5). In *A. septentrionalis* and *H. palustris*, centromeres were also enriched in long interspersed nuclear elements (LINEs), as observed in other angiosperms (*33*, *34*). In addition, we identified several telomeric repeats, providing additional evidence of assembly quality (figs. S3 to S9 and table S6).

**Table 1.** Summary of the genome assemblies generated for this study.

	*A. septentrionalis*	*A. vitaliana* (pin haplotype)	*A. vitaliana* (thrum haplotype)	*A. wulfeniana*	*H. inflata*	*H. palustris* (pin haplotype)	*H. palustris* (thrum haplotype)
Chromosome number	2*n* = 2*x* = 20	2*n* = 4*x* = 40		2*n* = 4*x* = 40	2*n* = 2*x* = 22	2*n* = 2*x* = 20	
Genome size estimate (flow cytometry; Mbp/1C)	601	437		517	Not available	800	
Genome size estimate (*k*-mers; Mb)	484.55	375.69	374.25	379.20	595.08	650.87	650.87
Assembly size (Mb)	494.22	422.41	416.69	426.45	592.12	834.08	794.25
No. contigs	412	1,615	1,573	2,971	140	2,216	732
N50 (Mb)	50.12	19.06	19.21	18.63	53.8	70.3	71.06
L50	5	11	11	11	5	5	5
Telomeres identified	19/20	11/40	11/40	4/40	20/22	7/20	18/20
Centromeres identified	6/10	18/20	16/20	7/20	11/11	9/10	10/10
Gene number	27,217	41,502	41,062	40,131	21,608	21,902	22,265
TE content	51.86%	29.08%	28.55%	28.69%	64.24%	68.93%	69.25%
BUSCO (genome)	95.6%	95.8%	95.7%	95.7%	95.7%	95.4%	95.7%
BUSCO (proteome)	86.5%	86.2%	87.3%	87.9%	96.1%	95.4%	95.9%

Gene annotation was performed using a combination of ab initio, evidence-based, and comparative gene-prediction approaches, incorporating RNA sequencing (RNA-seq) data from both vegetative and reproductive tissues (tables S7 to S14). High Benchmarking Universal Single-Copy Orthologs (BUSCO) completeness scores were obtained for all gene annotations and for the genome assemblies (fig. S10).

Comparative genomic analyses revealed an overall lack of whole-genome synteny among genera (fig. S11). Furthermore, we identified a whole-genome duplication (WGD) that likely occurred in the common ancestor of *A. vitaliana* and *A. wulfeniana* but was not shared with *A. septentrionalis* (fig. S12), confirming previous studies (*35*).

### The *S*-locus of *Hottonia* and *Primula* evolved in their common ancestor

**Fig. 2.**
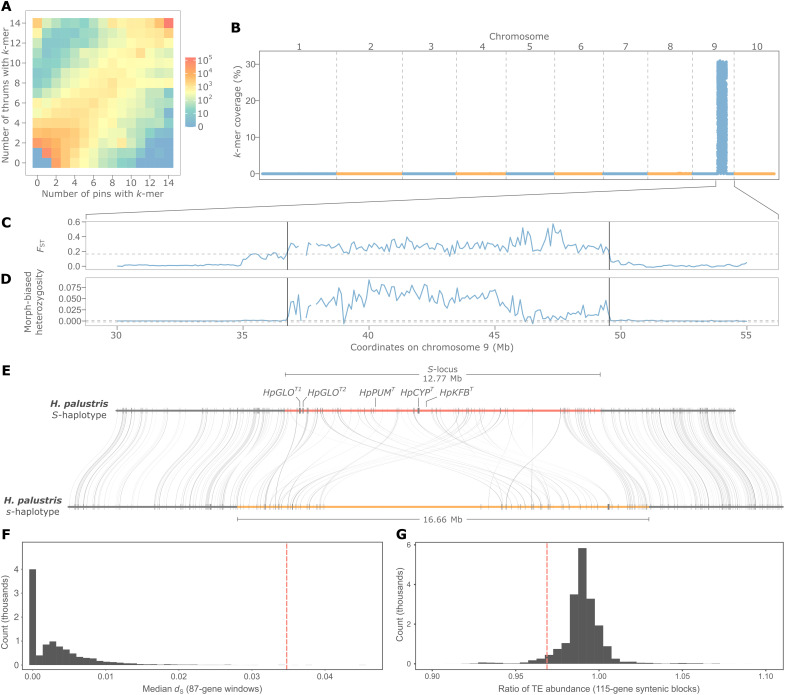
The *H. palustris S*-locus is heterozygous but contains hemizygous genes, including the orthologs of *Primula S*-genes. (**A**) Heatmap illustrating *k*-mer distribution across combinations of pin (*x* axis) and thrum (*y* axis) individuals. Each cell’s color represents the *k*-mer count for that specific combination of thrums and pins. The increased *k*-mer count in the top-left corner compared to the bottom-right corner indicates an abundance of thrum-specific over pin-specific *k*-mers. (**B**) Percentage of sequence covered by thrum-specific *k*-mers (calculated in 5-kb windows) across the *H. palustris* assembly. (**C** and **D**) Distribution of *F*_ST_ (C) and morph-biased heterozygosity (D), in 100-kb windows across chromosome 9 (chr9): 30,000,000 to 55,000,000 bp. The *S*-locus borders are marked by black vertical lines. Dashed horizontal lines represent the 95th percentile of the two distributions. (**E**) Microsynteny between the *S*-haplotypes (red) and *s*-haplotypes (orange). The orthologs to *Primula S*-genes are labeled in the figure. (**F**) Distribution of median *d*_S_ calculated in 87-gene windows between the two haploid assemblies. The red dashed vertical line represents the median *d*_S_ for the 87 genes at the *S*-locus present in both haplotypes (median *d*_S_ = 0.035), which is significantly higher than the genomic background (empirical two-sided *P* < 0.01). (**G**) Distribution of the ratio of TE abundance between 115-gene syntenic windows (*n* = 19,187), calculated across the genome. The red dashed vertical line represents the ratio of TE abundance (*S*/*s* = 0.935) between the *S*- and *s*-haplotypes, which significantly differs from the genomic background (empirical two-sided *P* < 0.01).

To find the genetic basis of distyly in *H. palustris*, we generated short-read sequencing data from 28 individuals (14 pins and 14 thrums; hereafter “population dataset”) and searched for genetic differences associated with morphs. First, we determined which morph carries the *S/s* genotype by searching for morph-specific *k*-mers, i.e., sequences present in one floral morph but absent in the other. We identified 7,023,864 thrum-specific and only 107 pin-specific *k*-mers, suggesting that thrums bear the *S/s* genotype (Fig. 2A), as in all distylous species studied to date (*13*, *16*–*25*). Since the *S*-locus cosegregates with the thrum phenotype, we aligned the thrum-specific *k*-mers to the genome assembly to search whether they mapped in a single region, an approach similar to that used to identify the sex-determining region in heterogametic systems (*36*–*38*). Most (99.99%) thrum-specific *k*-mers mapped to a 12.77-Mb region on chromosome 9 (36.81 to 49.56 Mb) spanning 115 genes and encompassing the putative centromere (Fig. 2B and figs. S13 and S14). This same region displayed elevated *F*_ST_ (genetic differentiation) and *D_XY_* (absolute sequence divergence) between morphs, increased heterozygosity in thrums relative to pins (calculated as the frequency of heterozygotes in 5-kb windows), and strong linkage disequilibrium (Fig. 2, C and D, and figs. S15 and S16). Together, these lines of evidence unambiguously pinpoint this region as the *S*-locus.

We next characterized the structure of the *S*-locus by performing a synteny analysis between the two *H. palustris* haplotypes (Fig. 2E). This analysis revealed that the *S*-locus was mostly heterozygous, with the *S*- and *s*-haplotypes containing 115 and 120 genes, respectively. Coverage analysis across the 28 individuals of the population dataset confirmed consistent gene presence-absence differences: 25 genes were *s*-specific and 22 were *S*-specific across individuals, while 87 genes were present in both haplotypes. The remaining genes (eight in the *s*-haplotype and six in the *S*-haplotype) showed variable presence-absence patterns across individuals (figs. S17 and S18 and tables S15 and S16). In addition to gene presence-absence variation, the two *S*-locus haplotypes were characterized by elevated synonymous divergence (*d*_S_) compared to the genomic background (Fig. 2F) and multiple rearrangements, whereas the flanking regions were highly syntenic (Fig. 2E). These observations suggest recombination suppression across the *S*-locus, consistent with the expectation for distyly *S*-loci. As nonrecombining regions, supergenes are also predicted to accumulate repetitive sequences, including TEs (*39*). However, we observed that TE abundance was significantly higher in the freely recombining *s*-haplotype (85.86%) than in the nonrecombining *S*-haplotype (80.31%) (empirical two-sided *P* < 0.01; Fig. 2G and table S17). This finding underscores that the prediction of TE accumulation in supergenes should be approached with caution, as TE accumulation is influenced by multiple factors, including the species’ evolutionary history and the age, architecture, and genomic location of the supergene (*40*–*42*).

Notably, 5 of the 22 *S*-haplotype–specific genes were orthologous to the *Primula S*-genes: *HpGLO^T1^* and *HpGLO^T2^* (tandemly duplicated copies of *Primula GLO^T^*), *HpPUM^T^*, *HpCYP^T^*, and *HpKFB^T^* (table S18). These five *S*-haplotype–specific genes of *H. palustris* displayed a similar expression pattern as in *Primula* (fig. S19). However, unlike in *Primula* species, where the *S*-genes occur in a fully hemizygous single block (*13*, *27*), in *H. palustris*, they are interspersed with genes present in both haplotypes across a 5.14-Mb region.

The occurrence of the same *S*-genes in the *S*-haplotypes of *Primula* and *H. palustris* suggests that the *S*-locus originated before the divergence between *Primula* and *Hottonia*, indicating that the *S*-loci in the two lineages are orthologous. If so, then the absence of distyly in *H. inflata* would reflect a secondary loss caused by loss-of-function mutations in its *S*-locus. We found that the *S*-locus of *H. inflata* was present but lacked both *CYP^T^* and *GLO^T^*, while *PUM^T^* and *KFB^T^* were retained in both haplotypes (figs. S20 and S21) and had similar expression patterns as in *Primula* (fig. S22) (*43*). These results contrast with those observed in the nondistylous *Primula grandis* (*44*) and in nondistylous populations of *Primula vulgaris* (*45*, *46*), in which the loss of distyly is associated with loss-of-function mutations in *CYP^T^* alone. Given the functions of *CYP^T^* and *GLO^T^* (*28*, *31*, *32*), the absence of both genes in *H. inflata* is fully consistent with the loss of both distyly and self-incompatibility in this species (*47*).

Our results show that the *S*-locus of *Primula* and *Hottonia* originated in their common ancestor and was independently lost in nondistylous *Primula* species and *H. inflata*, contrary to previous phylogenetic analyses of phenotypic trait evolution that had inferred independent origins of distyly in *Primula* and *H. palustris* (*29*). Unlike the fully hemizygous *S*-loci characterized so far (*13*, *16*–*18*, *20*–*24*), the *H. palustris S*-locus contains both heterozygous and hemizygous genes, as in the distantly related *Turnera subulata* and *Cordia subcordata* (*19*, *25*). However, unlike these two species, where the hemizygous *S*-genes cluster together to form a contiguous hemizygous block, in *H. palustris*, the hemizygous *S*-genes are interspersed among genes present in both haplotypes (Fig. 2E). Furthermore, the genomic region containing the *S*-locus in *H. palustris* (which is on chromosome 9) was not syntenic to the one containing the *S*-locus in either *Primula veris* or *Primula edelbergii* [in chromosomes 1 and 2, respectively (*27*, *30*); fig. S23], suggesting that the *S*-locus was translocated to other chromosomes at least twice since its origin in the most recent common ancestor (MRCA) of the two genera. However, despite multiple translocations, the *S*-locus retained a pericentromeric location in all three species. In *H. palustris*, the centromere lies within the *S*-locus; in *P. edelbergii*, the *S*-locus is located 3.30 Mb from the centromere (4.95% of the chromosome length) (*30*); in *P. veris*, the *S*-locus is located 0.23 Mb from the centromere (0.47% of the chromosome length). This observation may reflect the propensity of pericentromeric regions for gene gains, losses, and structural rearrangements (*48*), all of which could have influenced the evolution of the *S*-locus in these species.

### The *A. vitaliana S*-locus originated independently from the *Primula*/*Hottonia S*-locus

**Fig. 3.**
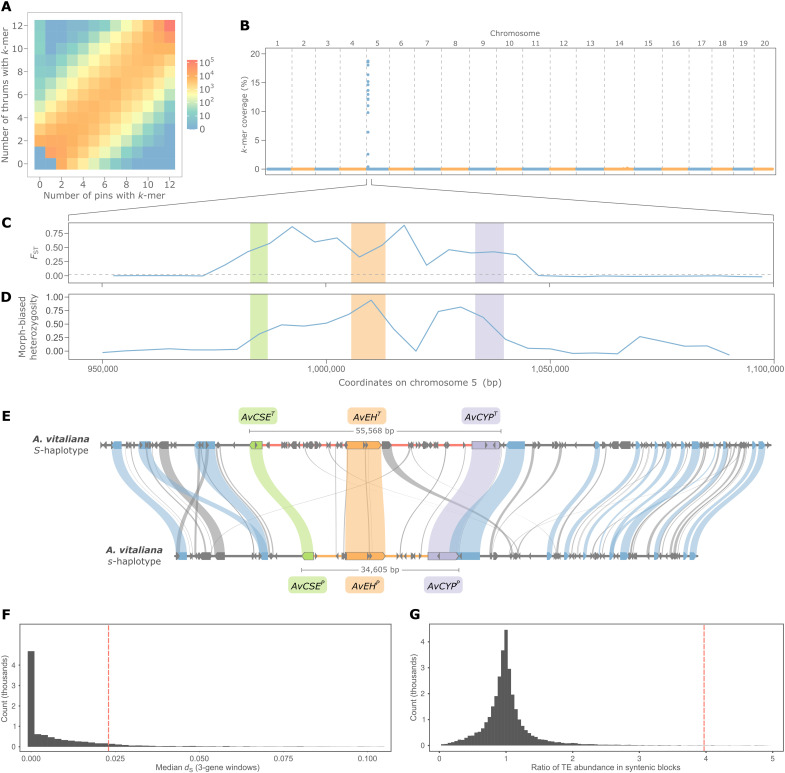
The *A. vitaliana S*-locus is heterozygous and consists of three genes, including one orthologous to a *Primula S*-gene (*CYP^T^*). (**A**) Heatmap illustrating *k*-mer distribution across combinations of pin (*x* axis) and thrum (*y* axis) individuals. Each cell’s color represents the *k*-mer count for that specific combination of thrums and pins. The increased *k*-mer count in the top-left corner compared to the bottom-right corner indicates an abundance of thrum-specific over pin-specific *k*-mers. (**B**) Percentage of sequence covered by thrum-specific *k*-mers (calculated in 5-kb windows) in the *A. vitaliana* assembly. (**C**) *F*_ST_ distribution, calculated in 5-kb windows, across chromosome 5: 950,000 to 1,100,000 bp. The increase in *F*_ST_ overlaps with the region enriched for thrum-specific *k*-mers and represents the *S*-locus, whose three genes are highlighted by colored blocks. (**D**) Distribution of morph-biased heterozygosity, calculated as the number of thrums carrying the heterozygous genotype minus the number of pins carrying the heterozygous genotype for 882 single-nucleotide polymorphisms (SNPs; chromosome 5: 950,000 to 1,100,000 bp) identified in 16 individuals (8 thrums and 8 pins) and averaged in 5-kb windows. (**E**) Microsynteny between the *S*- and *s*-haplotype. Genes and TEs are represented as blue and gray rectangles, respectively; *S*-genes are colored in green (*AvCSE*), orange (*AvEH*), and purple (*AvCYP*); the *S*-haplotype is marked as a red line, while the *s*-haplotype as an orange line. (**F**) Distribution of median *d*_S_ calculated in three-gene windows between the two haploid assemblies. The red dashed vertical line represents the median *d*_S_ for the three genes at the *S*-locus (median *d*_S_ = 0.0023), which does not significantly differ from the genomic background (empirical two-sided *P* > 0.05). (**G**) Distribution of the ratio of TE abundance between three-gene syntenic windows (*n* = 32,872), calculated across the genome. The red dashed vertical line represents the ratio of TE abundance (*S*/*s* = 3.660) between the *S*- and *s*-haplotypes, which is significantly higher than the genomic background (empirical two-sided *P* < 0.05).

To search for the *S*-locus of *A. vitaliana*, we adopted the same approach as for *H. palustris* (see above). We generated two short-read datasets, one consisting of 24 individuals (12 pins and 12 thrums) collected in the Wallis canton in Switzerland (“Wallis dataset”) and one consisting of 14 individuals (7 pins and 7 thrums) obtained from herbarium specimens (“herbarium dataset”). The results reported below refer to the Wallis dataset; consistent results were observed in the herbarium dataset and are reported in the Supplementary Materials. We detected more thrum-specific than pin-specific *k*-mers (18,990 versus 2896), indicating that thrums bear the *S/s* genotype also in this species (Fig. 3A). Most thrum-specific *k*-mers, as well as elevated *F*_ST_ and increased heterozygosity in thrums compared to pins, were observed in a ~70-kb region at one end of chromosome 5 [975,000 to 1,045,000 bp; Fig. 3, B to D, and figs. S24 to S27], thereby identifying this region as the *S*-locus.

Synteny analysis showed that three genes are contained in the *A. vitaliana S*-locus and are present in both haplotypes in the same order and orientation, with synteny extending into the *S*-locus flanking regions (Fig. 3E). Following the same naming convention used in *Primula* (*13*), we name these genes *AvCSE^T^*, *AvEH^T^*, and *AvCYP^T^* for *S*-alleles, where “*T*” refers to thrums, and *AvCSE^P^*, *AvEH^P^*, and *AvCYP^P^* for *s*-alleles, where “*P*” refers to pins. Caffeoyl shikimate esterases (*CSE*) participate in lignin biosynthesis, while epoxide hydrolases (*EH*) are involved in lipid biosynthesis, and both are presumed or confirmed to be involved in defense against pathogens (*49*, *50*). Since genes involved in defense, lipid and lignin biosynthesis are also differentially regulated in response to incompatible pollination in other species (*51*), we speculate that *AvCSE^T^* and *AvEH^T^* may play a role in self-incompatibility. If the *S*-alleles played a role in controlling distyly, then we would expect them to be up-regulated compared to *s*-alleles in floral tissues. We observed that, in thrum flowers, *AvEH^T^* and *AvCYP^T^* were up-regulated compared to *AvEH^P^* and *AvCYP^P^* (fig. S28), while *AvCSE* alleles were expressed at the same level. Our results therefore are consistent with the hypothesis that the *S*-alleles *AvEH^T^* and *AvCYP^T^* control distyly in *A. vitaliana*. Unlike the *H. palustris S*-locus, we did not detect elevated *d*_S_ in *A. vitaliana S*-genes (Fig. 3F). Nevertheless, this region contained sequence variants in strong linkage disequilibrium that cosegregate with floral morphs (figs. S29 to S32), and TE density in the *S*-haplotype (25.40%) was significantly higher than that of the *s*-haplotype (6.94%) (empirical two-sided *P* < 0.05; Fig. 3G, fig. S33, and table S17), consistent with the classical expectations for a nonrecombining supergene.

Together, the presence of three tightly linked genes carrying variants that cosegregate with floral morph, combined with allele-specific upregulation for two of them in floral tissues, indicates that the entire three-gene block represents the *S*-locus supergene in *A. vitaliana*. However, it is also possible that only one of these genes determines floral morph, e.g., by acting as a master regulator, and that the linked variants and TEs are coinherited along with it without contributing to the phenotype. Functional studies will therefore be required to determine the specific contribution of each gene to distyly.

The *A. vitaliana S*-locus represents the first distyly *S*-locus that is entirely heterozygous, for it consists of three genes present in both haplotypes. This contrasts with all previously characterized *S*-loci, in which the genes controlling floral morphs are hemizygous in thrums and absent from pins. Another distinctive feature of the *A. vitaliana S*-locus is its nonpericentromeric location: It is located near the end of chromosome 5, ~7.3 Mb away from the centromere (~43% of the chromosome length), unlike the pericentromeric *S*-loci of *Primula* and *Hottonia*. It has been proposed that supergenes are more likely to emerge in regions already characterized by reduced recombination, such as pericentromeric regions (*3*, *5*, *52*). Several examples support this hypothesis, such as the sex-determining regions of kiwifruit (*37*), *Nepenthes* (*53*), and *Rumex* (*54*), and the self-incompatibility locus in *Petunia* (*55*). However, the *A. vitaliana S*-locus, similar to other supergenes that are not embedded in a broader low-recombining region [e.g., the distyly *S*-locus of *Linum tenue* (*20*)], challenges this hypothesis and suggests that supergenes can originate without being located in regions already characterized by reduced recombination. Last, we note that *A. vitaliana* represents another example of an independently evolved *S*-locus containing a gene involved in brassinosteroid inactivation, *AvCYP^T^*.

### The same gene (*CYP^T^*) was independently co-opted in the *S*-loci of *Androsace* and *Primula/Hottonia*

Previous analyses on *Primula* showed that the *S*-locus evolved via multiple, asynchronous gene duplications and independent translocations (*27*), followed by neofunctionalization of the two key *S*-genes: *CYP^T^* acquired a style-specific expression (*31*), while *GLO^T^* became involved in controlling floral-organ growth (*28*). Dating these gene duplication events is therefore crucial for estimating when the *S*-genes originated and providing an upper bound on the timing of their neofunctionalization. Previous phylogenetic analyses on the *S*-locus in *Primula* estimated the duplication ages of *CYP^T^* and *GLO^T^* around 43 and 37 million years ago (Ma), respectively, thus preceding *Primula* divergence from *Hottonia* by 12 to 18 Myr (*13*, *27*, *29*). However, the sparse sampling of *Primula*’s closest relatives left much uncertainty regarding the duplication ages of these key *S*-genes.

**Fig. 4.**
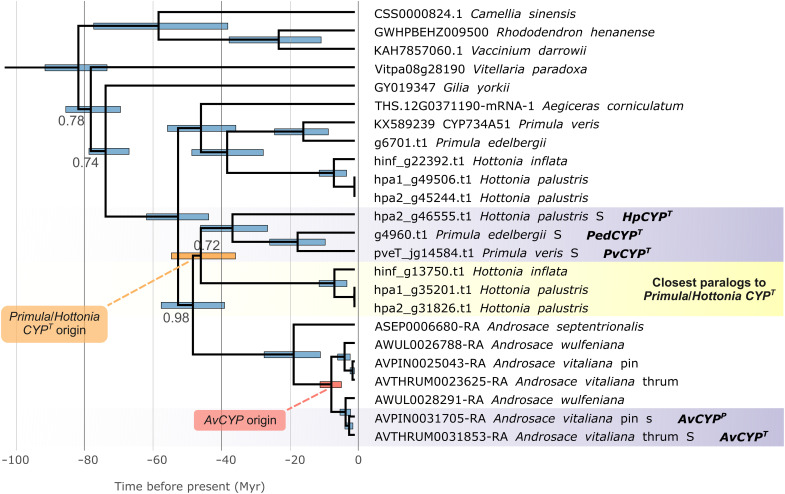
Phylogeny of the *S*-gene CYP^T^ and close homologs. Bayesian chronogram of *CYP734* nucleotide sequences in selected genomes. Bottom scale bar indicates time before present in million years. Blue bars at nodes represent 95% Bayesian credibility intervals around age estimates, and the orange bar represents the same for the duplication event that gave rise to *CYP^T^* in *Primula* and *Hottonia*, while the red bar represents the same for the *Androsace* WGD that gave rise to *AvCYP*. The closest paralogs to the *Primula*/*Hottonia CYP^T^* (which are now present only in *Hottonia* species) are highlighted in yellow. Branch labels represent posterior probabilities of <1, while those without a numeric label have posterior probabilities of 1. The nucleotide sequences were aligned at the amino acid level and filtered with OMM_MACSE resulting in a 1713-bp-long alignment with 16.9% gap or ambiguous characters and 56.6% average pairwise similarity.

The genomic data presented here allowed us to improve estimates of *S*-gene phylogenetic histories (figs. S34 to S40). We inferred node-dated phylogenies for all gene families comprising the *S*-genes identified in Primulaceae. Five gene phylogenies (*CYP*, *GLO*, *KFB*, and *PUM*, *EH*; figs. S35 to S38 and S40) supported the previously identified *Pv-*α WGD at the root of Primulaceae (*27*), dating it at 53 (35 to 81) Ma. The topologies of the *KFB* and *PUM* phylogenies suggest that *KFB^T^* and *PUM^T^* originated through the *Pv-*α WGD (figs. S37 and S38). Conversely, the other *S*-genes in these two genera originated through more recent duplication events (*CCM^T^*, 7.7 Ma; *GLO^T^*, 36 Ma; *CYP^T^*, 45 Ma), as evidenced by gene tree topology. These duplicates were strongly supported as nested within the older *Pv-*α WGD event in their respective estimated phylogenies (Fig. 4 and figs. S34 to S36). Furthermore, our analyses revealed that the closest paralog of *CYP^T^* is not *CYP734A51*, as previously suggested (*31*), but rather a newly found paralog present in *Hottonia* genomes (*hinf_g13750*, *hpa1_g35201*, and *hpa2_g31826*) but absent from all *Primula* genomes sequenced to date (Fig. 4). The gene phylogenies of all *S*-genes except *CCM* (figs. S35 to S40) also strongly support a WGD event shared between *A. vitaliana* and *A. wulfeniana* around 8.0 (3.7 to 24.0) Ma, before the divergence between the *S-* and *s*-alleles of *A. vitaliana* dated at 7.3 (2.7 to 13.0) Ma for *EH*, 1.7 (0.6 to 2.9) Ma for *CYP*, and 1.4 (0.3 to 3.0) Ma for *CSE* (Fig. 4 and figs. S35, S39, and S40).

Our results show that *AvCYP^T^* originated via duplication of the same gene whose duplication gave origin to the *Primula*/*Hottonia CYP^T^*. This means that duplicates of the same gene were independently and asynchronously co-opted to create the distylous phenotype, first in the MRCA of *Primula* and *Hottonia* and later in *A. vitaliana*. In both cases, neofunctionalization of *CYP^T^* was preceded by a duplication event: In the MRCA of *Primula* and *Hottonia*, this was an isolated duplication followed by translocation to the *S*-locus, while in *A. vitaliana*, this occurred in the *Androsace*-specific WGD. However, all these gene copies coalesce in the MRCA of Primulaceae, making them orthologs likely with the same or very similar functions.

These results demonstrate that the convergent evolution of distyly occurred via repeated co-option of the same brassinosteroid-inactivating gene, *CYP^T^*, within Primulaceae. Since genes affecting brassinosteroid inactivation have been reported in most *S*-loci studied to date (*13*–*18*), this may suggest that developmental or genetic constraints favor the repeated recruitment of brassinosteroid-related genes in the evolution of distyly. Alternatively, given the broad developmental roles of brassinosteroids in plant development (*56*), the repeated involvement of brassinosteroid-inactivating genes in distyly may just be a consequence of the large number of brassinosteroid-related genes in plant genomes. Further comparative analyses will be required to discriminate between these two alternative hypotheses.

### The *S*-locus of *A. vitaliana* originated via a selective sieve

Two main models have been proposed for the origin of supergenes. The “translocation” model posits that functionally interacting but genomically dispersed genes are later brought into proximity via translocation (*57*). The *S*-loci of most distylous species studied to date evolved in accordance with this model, with the *S*-genes originating via stepwise duplications and translocations (*58*). Conversely, the “Turner’s sieve” model posits that supergenes may arise via mutations in genes that are already physically linked, when these mutations confer a fitness advantage (*59*). In the case of distyly *S*-loci, a “segmental duplication” model has been proposed, whereby a genomic block containing multiple genes was duplicated as a unit (for example, through segmental duplication or WGD), after which mutations in the duplicated copies contributed to the evolution of the *S*-locus (*60*) . Under this scenario, the *S*-gene progenitors were already linked before acquiring their role in the control of distyly, making this model consistent with the Turner’s sieve model.

To test whether the physical linkage among *AvCSE*, *AvEH*, and *AvCYP* precedes the emergence of distyly, we performed synteny analyses among nine Primulaceae genomes of both distylous and nondistylous species and observed that these three genes are collinear across all species, including the nondistylous *Aegiceras corniculatum*, a species sister to the clade comprising *Primula*, *Hottonia*, and *Androsace* (fig. S41). This suggests that the progenitors of *AvCSE*, *AvEH*, and *AvCYP* already colocalized before the mutations that led to their involvement in the control of distyly. These findings also challenge the hypothesis that hemizygosity is an intrinsic feature established at the origin of distyly supergenes. Instead, they raise the possibility that hemizygosity may arise secondarily through gene losses from the *s*-haplotype. Because distyly evolved in *A. vitaliana* more recently than in *Primula*/*Hottonia*, its diallelic *S*-locus may represent an early stage in the evolution of *S*-loci that precedes the emergence of hemizygosity. Alternatively, it may reflect an independent evolutionary route by which distyly supergenes can arise.

Our results show that the *S*-locus of *A. vitaliana* evolved according to the Turner’s sieve model (*59*) and consistently with the segmental duplication model (*60*), as its *S*-genes duplicated simultaneously, as a single block, through a WGD. This scenario parallels recent findings in Oleaceae, where the *S*-locus controlling self-incompatibility and distyly also arose via neofunctionalization of colocalizing genes following WGD, albeit involving a different gene set (*22*).

### Two evolutionary strata in the expanded *S*-locus of *H. palustris*

The *S*-locus is much larger in *H. palustris* (12.75 Mb; 115 genes) than in *Primula* (~260 kb; 4 to 5 genes), despite sharing a common origin. Two scenarios could explain this: Either suppressed recombination expanded beyond the original *S*-locus in *H. palustris*, thereby increasing the size of the nonrecombining, morph-linked region (de facto increasing *S*-locus size), or the *S*-locus was originally larger and later contracted in *Primula*.

**Fig. 5.**
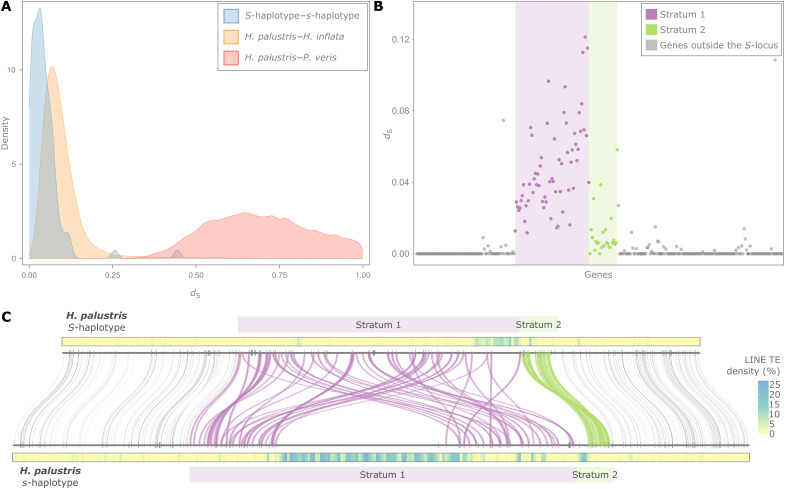
Expansion of suppressed recombination in the *H. palustris S*-locus. (**A**) Distributions of *d*_S_ calculated between syntenic orthologs of *H. palustris–P. veris* (*n* = 15,964; red curve), *H. palustris–H. inflata* (*n* = 18,830; orange curve), and between the *S*- and *s*-haplotype of *H. palustris* (*n* = 87; blue curve). The suppression of recombination in the *H. palustris S*-locus (blue curve) is more recent than the divergence between *H. palustris* and *P. veris* (red curve) and overlapping with the divergence between *H. palustris* and *H. inflata* (orange curve). (**B**) *d*_S_ values obtained between the *S*- and *s*-alleles for the 87 *S*-genes present in both haplotypes, as well as 93 and 151 additional genes at the left and right side of the *S*-locus, respectively. Genes were ordered on the basis of their position in the *s*-haplotype. The *d*_S_ distribution follows a stair-like pattern, indicative of two evolutionary strata: stratum 1 (purple) and stratum 2 (green). (**C**) Microsynteny between the *S*- and *s*-haplotypes of *H. palustris*. On top and bottom of the two haplotypes are density plots representing the proportion of sequence covered by LINE TEs (calculated in 100-kb windows), which are enriched in centromeric regions (see also figs. S8, S9, and S14). Ribbons connect syntenic genes in the two haplotypes (purple for genes in stratum 1, green for genes in stratum 2, and gray for genes outside the *S*-locus).

To disentangle these two possibilities, we calculated *d*_S_ between the two *S*-locus haplotypes of *H. palustris* and between *H. palustris* and *P. veris*. The results showed that divergence between the *H. palustris S*- and *s*-haplotypes was lower than divergence between *H. palustris* and *Primula* orthologous genes, indicating that recombination suppression between *S*-locus haplotypes in *H. palustris* occurred after the split from *Primula* (Fig. 5A). In addition, the genomic regions syntenic to the *H. palustris S*-locus in *Primula* and *Androsace* contain largely overlapping sets of genes (fig. S23 and table S19). Given the phylogenetic relationships among these genera, with *Androsace* sister to the clade comprising *Primula* and *Hottonia*, this shared gene content likely reflects the ancestral state of this region. The additional genes present within the *H. palustris S*-locus are therefore more parsimoniously explained by lineage-specific incorporation of genes into the nonrecombining region in *H. palustris*, rather than by extensive gene losses in *Primula*. Together, these observations indicate that the *S*-locus expanded in *H. palustris*, representing the first description of suppressed recombination expansion beyond a hemizygous supergene.

Several sex-determining regions and supergenes were shown to have expanded via successive, localized events of recombination suppression, leaving a stair-like pattern of divergence across the region (“evolutionary strata”) (*61*–*63*). To investigate whether a similar process occurred in the *S*-locus of *H. palustris*, we examined in more detail the *d*_S_ between the *S*- and *s*-alleles. We identified two evolutionary strata characterized by significantly different synonymous divergence (Wilcoxon rank-sum test, *P* < 0.001): an older stratum comprising 63 genes (stratum 1; *d*_S_ = 0.012 to 0.121) and a younger stratum comprising 24 genes (stratum 2; *d*_S_ = 0 to 0.058; Fig. 5B). Genes within the older stratum show more structural rearrangements between haplotypes, and the most highly differentiated among them lie on opposite sides of the centromere (marked by elevated LINE density) in the two haplotypes (Fig. 5C). This pattern suggests that the centromere, as well as the locally reduced recombination typical of pericentromeric regions, may have facilitated the initial expansion of recombination suppression around the *S*-locus. A plausible scenario is that structural changes in the immediate flanking regions of a hemizygous *S*-locus first extended the nonrecombining block (stratum 1), with later, more limited rearrangements generating stratum 2 (Fig. 5C). These observations suggest that, in *H. palustris*, the centromere likely contributed to the evolution of the *S*-locus. A comparable involvement of centromeres has been suggested for the evolution of other supergenes (*62*) and in the formation of sex-determining regions in several plant lineages (*37*, *38*, *64*).

**Fig. 6.**
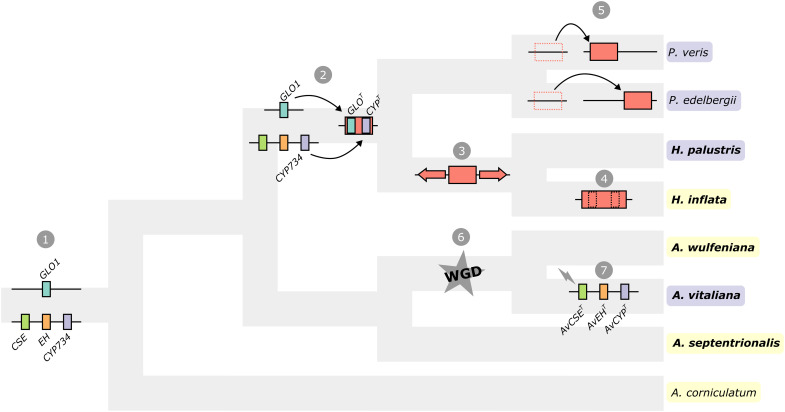
Simplified model of *S*-locus evolution in Primulaceae. Phylogeny of the species investigated in the present study summarizing the key events in the evolution of the *S*-locus in Primulaceae (distylous species, purple; nondistylous species, yellow). The ancestral genomic configuration (1) involved three colocalized (*CSE*, *EH*, and *CYP734*) and one noncolocalized gene (*GLO1*). Subsequently, duplications of *GLO1* and *CYP734* in the common ancestor of *Primula* and *Hottonia* (2) gave origin to *GLO^T^* and *CYP^T^*, which were then translocated to the same region, forming the core genes of the hemizygous *S*-locus (red block). In *Hottonia*, the *S*-locus then expanded, incorporating other heterozygous genes (3), while in *H. inflata*, the loss of *CYP^T^* and *GLO^T^* led to the loss of distyly (4). The genomic location of the *S*-locus differs among *P. veris*, *P. edelbergii*, and *Hottonia* species, implying at least two translocation events to different genomic locations (5). Conversely, in *Androsace*, *CYP734* was duplicated along with *CSE* and *EH* in a WGD that occurred in the common ancestor of *A. wulfeniana* and *A. vitaliana* (6) but only in the latter species these genes neofunctionalized (here symbolized by a bolt), acquiring a role in controlling distyly (7). For clarity, only the most salient evolutionary events are shown, omitting these details as differences in hemizygosity and TE accumulation.

In conclusion, by investigating the convergent evolution of the *S*-locus controlling distyly in Primulaceae, our study illustrates how evolution operates as an exploration of genomic possibilities, evidenced by the diverse architectures and genes underpinning distyly across the examined species (Fig. 6). However, this flexibility is tempered by molecular and evolutionary constraints. The results presented here align with Jacob’s concept of evolutionary tinkering (*65*), which suggests that evolution operates by repurposing existing components, and support Ohno’s hypothesis (*66*) highlighting the importance of gene duplications in the evolution of novel phenotypes. Within this theoretical framework, the repeated co-option of *CYP^T^* in controlling distyly underscores how evolutionary innovation is often context dependent and constrained by the available genetic toolkit, leading to convergent outcomes through the modification of preexisting elements. Together, our study not only illuminates the mechanisms underlying supergene evolution but also contributes to a broader understanding of how genomic innovation and constraint interact in convergent evolution.

## MATERIALS AND METHODS

### Material collection

The pin and thrum plants used to generate the *A. vitaliana* subsp. *lepontina* reference genomes were collected by the Gibidumsee lake of Visperterminen in Canton Wallis (coordinates 46.2577, 7.9396), and voucher specimens were deposited at the Zürich University Herbarium (accession number: Z-000227551). An additional 24 accessions (comprising 12 pins and 12 thrums) were collected from the Visperterminen and Zermatt areas in Canton Wallis (accession numbers: Z-000227549, Z-000227550, and Z-000227552) and 14 accessions (7 pins and 7 thrums) sampled directly from herbarium specimens of the Zürich University Herbarium and Real Jardín Botánico Madrid Herbarium collected in the French Alps (pin and thrum: Z-000195747), Pyrenees (pin: MA 320297 and thrum: MA 320297), Cantabrian (pin: MA 493665 and thrum: MA 493665), Spanish Central System (pin: MA 532234 and thrum: MA 560365), Nevada Mountains (pin: MA 888351 and thrum: MA 889193), Montes Aquilanos (pin: MA 280017 and thrum: MA 280016), the Apennines (pin: MA 698758), and Dolomites (thrum: MA 353134) for whole-genome resequencing (WGS) with Illumina (see below). For RNA-seq, we collected leaves and flowers in RNAlater (Thermo Fisher Scientific) from the Canton Wallis population.

The plant used to generate the *H. palustris* reference genome assembly was collected in the Burgwies pond in Zurich (coordinates 47.3569, 8.5747) and documented on iNaturalist (www.inaturalist.org/observations/41758573). Additional 28 accessions (comprising 14 pins and 14 thrums) were collected in Switzerland, France and Germany for WGS with Illumina (see below).

The *H. inflata* individual used to generate the reference genome assembly was collected from Carbondale, IL, USA (specimen deposited at the Marie-Victorin Herbarium, Lacroix-Carignan 1467, MT) and shipped to Zürich for HiFi and RNA-seq and to Arima Genomics (CA, USA) for Hi-C sequencing. For RNA-seq, we used leaves, buds, and flowers that were collected in RNAlater (Thermo Fisher Scientific) or fresh material.

### DNA extraction and sequencing

To generate the genome assembly of each species, DNA was extracted from fresh leaves using a modified cetyltrimethylammonium bromide (CTAB) protocol, specific for high–molecular weight DNA isolation (*67*). DNA sequencing was then performed with Oxford Nanopore Technologies (ONT) and Illumina platforms for *Androsace* species and PacBio HiFi for *Hottonia* species.

For *Androsace* species, ONT libraries were prepared using the SQK-LSK108 kit and sequenced on MinION and PromethION R9 flow cells for 48 to 72 hours to achieve a minimum of 60× coverage. Basecalling was done using the methylation-aware (MinION) and high-accuracy (PromethION) models in Guppy v.3.3.3. In addition, ~110× of Illumina 150-bp paired-end (PE) reads (300-bp insert size) was generated on a NovaSeq 6000 for the *Androsace* species. PacBio HiFi sequencing was done using the Sequencer PacBio II and PacBio IIe for *H. palustris* and *H. inflata*, respectively, at the Functional Genomics Center Zürich (FGCZ), Switzerland. For WGS data generation, DNA was extracted from dried samples or herbarium specimens (for *A. vitaliana*) using a modified CTAB protocol (*61*). TrueSeq Illumina libraries were generated and sequenced on a NovaSeq platform at the FGCZ (150-bp PE reads).

Chromatin-conformation capture methods were used to aid genome scaffolding. Specifically, for *Androsace* genomes, Hi-C libraries were generated using a previously published protocol (*68*) and sequenced on an Illumina NovaSeq 6000; for *H. palustris,* Omni-C libraries were prepared using the Omni-C library preparation kit (Dovetail, USA), loaded for PE sequencing on an Illumina NovaSeq 6000 system, and run in XP mode using a NovaSeq 6000 SP Reagent Kits v1.5 (300 cycles). We set a single index running mode to 6:151:151:0 cycles. For *H. inflata*, Hi-C libraries were prepared using the Arima library preparation kit (Arima Genomics, USA) and sequenced on an Illumina NovaSeq. Statistics on short- and long-read sequencing data were obtained with SeqKit (*69*) v2.9.0 and NanoStat (*70*) v1.1.2, respectively.

### RNA extraction and sequencing

For *H. palustris*, we extracted RNA from leaves, buds (young and old), and flowers using the Spectrum Plant Total RNA Kit (Sigma-Aldrich). TrueSeq Stranded mRNA libraries were generated for each sample and sequenced on a NovaSeq 6000 at the FGCZ (Switzerland) to generate 20 million 150-bp PE reads per sample.

RNA was isolated from vegetative (leaves and stem) and reproductive (flowers and flower buds) tissue of *A. vitaliana* pins and thrums, vegetative tissue of *A. wulfeniana*, as well as vegetative and reproductive tissue of *A. septentrionalis* with the Spectrum Plant Total RNA Kit (Sigma-Aldrich), and RNA integrity was checked on a TapeStation 6000 (Agilent Technologies). TruSeq Stranded mRNA libraries were prepared and sequenced on an Illumina NovaSeq 6000 to generate 100 million 150-bp PE reads per sample.

### Genome profiling

We estimated genome sizes using both a *k*-mer–based genome profiling and flow cytometry to assess whether our genome assembly sizes were close to the expected size. Genome profiling (i.e., estimate of genome size, repeat content, heterozygosity, and haplotype length) was performed via *k*-mer analysis on Illumina reads for *Androsace* and PacBio HiFi reads for *Hottonia*. First, *k*-mers (31-mers for *Androsace* and 21-mers for *Hottonia*) were counted with Jellyfish count (*71*) v2.2.10 (-C, -m 21). Then, we used Jellyfish histo to generate a suitable input file for the online version of GenomeScope (*72*) (qb.cshl.edu/genomescope) with the following parameters: for *Androsace*, *k*-mer length = 31, read length = 150, and max *k*-mer coverage = 10,000; for *Hottonia*, *k*-mer length = 21, read length = 10,000, and max *k*-mer coverage = 10,000.

To verify the results obtained with this *k*-mer–based approach, we also estimated genome sizes of *A. vitaliana*, *A. wulfeniana*, *A. septentrionalis*, and *H. palustris* with flow cytometry using one to four individuals per species (table S1), following a previously published protocol (*73*). Briefly, fresh leaf material of each sample was cochopped (*74*) with a reference of known genome size in Otto I buffer; the suspension was filtered, digested with ribonuclease, mixed with Otto II buffer, and stained with propidium iodide in the dark at 4°C for 1 to 24 hours. We used several broadly used genome-size references (*75*–*77*) (*Raphanus sativus* cv. “Saxa” 1C = 0.555 pg, *Solanum lycopersicum* cv. “Stupicke polni tyckove rane” 1C = 0.98 pg, or *Solanum pseudocapsicum* 1C = 1.295 pg). At least 7000 nuclei were analyzed on a Cytoflex S (Beckman Coulter) or a CyFlow ML (Partec; green laser 100 mW, 532 nm, Cobolt Samba, Cobolt AB) flow cytometer. Only nuclei peaks with coefficients of variation below 5% were analyzed.

### Genome assembly

*Androsace* assemblies were generated using both Illumina and Nanopore reads and the hybrid approach in MaSuRCA (*78*) v3.4.1 with the longest 35× raw ONT reads and all the 110× Illumina reads, polished with one round of POLCA (*79*), followed by one round of Pilon (*80*) v.1.23, and by mapping the trimmed Illumina reads with BWA-MEM (*81*) v.0.7.17. Organellar scaffolds were identified with tBLASTn (*82*) (BLAST v2.8.1; -evalue 1e-25, -max_target_seqs 1) using the chloroplast proteome of *P. veris* (GenBank accession: KX639823) and mitochondrial proteome of *Camellia sinensis* (GenBank accession: NC_043914.1) as queries, and contigs with >12 significant hits to plastid or mitochondrial genes were excluded from the main assembly. Allelic contigs (haplotigs) were identified and removed with Purge_dups (*83*) v1.2.3 using default settings except for a similarity threshold of 94%, a minimum fraction of 70%, and manual coverage cutoffs determined from the coverage histogram. After removing contaminant, organellar, and allelic contigs, the remaining contigs were scaffolded with Hi-C short reads with Juicer (*84*) v.1.5.7, 3d-dna (*85*) v180922, and HiC-Hiker (*86*) v.1.0.0, followed by manual adjustments in juicer v1.1 [Juicebox Assembly Tools (JBAT)] (*87*). Last, gaps in the assembly were closed with TGS-GapCloser (*88*) v.1.1.1 using uncorrected nanopore reads, polished with one round of Racon (*89*) v.1.4.3 and two rounds of Pilon (*80*) v.1.23.

For *A. vitaliana*, the pin haplotype assembly was obtained by sequencing a pin individual (homozygous for the *s*-haplotype), while the thrum assembly was obtained by sequencing a thrum individual. Because thrums are *S*/*s* heterozygotes, we expected the *S*- and *s*-haplotypes to be assembled separately. During the Hi-C scaffolding phase of the thrum genome assembly, an unscaffolded contig (contig “2035”) was identified as containing the *S*-alleles, based on the *k*-mer, *F*_ST_, and morph-biased heterozygosity analyses (see the “*S*-locus identification” section). Hi-C contact maps further confirmed that this contig was positioned in the same genomic region where the *s*-alleles had been scaffolded on chromosome 5 (fig. S42). Given that the *S*-alleles are thrum-specific, the contig containing them was manually placed in the corresponding position on chromosome 5, replacing the *s*-alleles also found in the pin genome assembly. This adjustment ensured that the reference *A. vitaliana* thrum genome assembly contained the *S*-alleles in its chromosome-scale scaffolds. The junction between the *S*-allele contig and adjoining contigs was subsequently gap-filled and polished to ensure assembly continuity. The *k*-mer, *F*_ST_, and morph-biased heterozygosity results presented here are based on this final reference genome. Chromosome numbers were arbitrarily assigned in the *Androsace* assemblies; homologous chromosome pairs in the assemblies of polyploid *Androsace* species were assigned consecutive numbers.

The *H. palustris* genome assembly was generated by combining PacBio HiFi long reads and Omni-C data in HiFiasm (*90*) v0.16.1. Both datasets were generated from a single thrum individual, and the assembly process produced a haplotype-phased genome. Each haplotype was then scaffolded using Hi-C reads in YaHS (*91*) v1.2. Chromosome numbering in *H. palustris* follows decreasing chromosome size, with chromosome 1 representing the largest. The *H. inflata* assembly was generated using PacBio HiFi long reads in HiFiasm (*90*) v0.16.1 and scaffolded using Hi-C reads in YaHS (*91*) v1.2. Before scaffolding, each assembly was screened for contaminant sequences using BlobTools (*92*) v1.1.1 in combination with the UniProt database (*93*) (The UniProt Consortium 2017), removing few (<1% of assembly length) contigs. A Hi-C contact map was then generated for each assembly and visualized in JBAT (*87*), allowing for the manual curation of misassemblies. Chromosome numbering in *H. inflata* is based on homology with *H. palustris*: Chromosomes 1 and 2 are homologous to parts of *H. palustris* chromosome 1, while chromosomes 3 to 11 of *H. inflata* have a 1:1 correspondence with *H. palustris* chromosomes 2 to 10.

The genome assembly of each species was searched for centromeric repeats using the *CentroMiner* tool of quarTeT (*94*) v1.2.0 (-n 70, -m 2000, -r 10). The candidate centromeric regions identified by quarTeT were then discarded if they overlapped with telomeric regions. A self-identity heatmap was generated with ModDotPlot (*95*) for each candidate centromeric region; if the heatmap of a candidate region did not show any large tandem repeat array, then this region was excluded. Telomeric repeats were identified in each genome independently with quarTeT TeloExplorer (*94*) v1.2.0, which searched for the TTTAGGG monomer repeated in tandem at least 50 times (-c plant, -m 50). The completeness of the assemblies was assessed with BUSCO (*96*) v.5.6.1 (-m genome), using the 2326 single-copy orthologs from the eudicot database (eudicots_odb10; creation date: 8 January 2024), while basic statistics on the assemblies were obtained with Quast (*97*) v5.0.2.

### TE annotation

Repetitive elements were identified in all assemblies using EDTA (*98*) (v1.8.3 and v1.9.4 for *Androsace* and *Hottonia* genome assemblies, respectively), which combines structure- and homology-based approaches for de novo TE identification. Structural discovery of TEs was achieved using LTRharvest (*99*) and LTR_retriever (*100*) for long terminal repeat (LTR) retrotransposons, TIR-Learner (*101*) for terminal inverted repeat (TIR) transposons, and HelitronScanner (*102*) for helitrons, generating a refined, nonredundant TE library for each genome assembly. Additional repetitive sequences were identified using RECON (*103*) v1.08 and RepeatScout (*104*) v1.06 through RepeatModeler (*105*) v2.0, resulting in a curated TE library specific to each assembly. The de novo TE libraries produced by EDTA were then used to annotate the respective assemblies using RepeatMasker (*106*) v4.0.9.

### Gene annotation

Gene annotation was conducted individually for each assembly by using a combination of ab initio and evidence-based methods and supported using both protein datasets and RNA-seq data from both vegetative and reproductive tissues (table S7). RNA-seq evidence comprised *A. vitaliana* (40 samples: 10 thrum flowers, 10 pin flowers, 10 thrum leaves, and 10 pin leaves), *A. septentrionalis* (2 samples: 1 flower and 1 leaf), *A. wulfeniana* (1 leaf), *H. inflata* (6 samples: 3 flowers and 3 inflorescence stems), and *H. palustris* (8 samples: 4 pin and 4 thrum individuals; each contributing 1 young floral bud, 1 old floral bud, 1 full-blooming flower, and 1 leaf). For *Androsace*, GeneMark (*107*) v.4 and AUGUSTUS (*108*) v.3.3.3 were trained for de novo gene prediction. To achieve this, RNA-seq reads were mapped to the assemblies [soft-masked with the *maskfasta* function of BEDtools (*109*) v2.28.0 (-soft)] using HISAT2 (*110*, *111*) v2.1.0 (--phred33, --very-sensitive, --max-intronlen 50000), and ab initio gene predictors were trained using BRAKER (*112*) v.2.1.5 in “etp mode” (development version). The MAKER (*113*) pipeline was then used to integrate the ab initio gene predictions, along with evidence from SwissProt Viridiplantae protein sequences and a transcriptome assembly generated for each species with Trinity (*114*) v2.11.0 to improve gene annotations. In the first round of MAKER, genes were predicted with five sources of evidence, while soft-masking the genome with the species-specific TE library: (i) the Trinity transcriptome assembly, (ii) SwissProt proteins, (iii) the gff3 obtained with BRAKER, (iv) the GeneMark HMM file, and (v) trained Augustus gene modes. The evidence alignments were turned into “hints,” and MAKER v3.01.03 was run iteratively two additional times using the transcriptomes as evidence. The resulting set of predicted genes were annotated with Pfam domains (*115*) using InterProScan (*116*), and the models were filtered selecting them if they had an annotation edit distance of <1 and/or PFAM domain. For *Hottonia*, GeneMark (*107*) v.4 and AUGUSTUS (*108*) v.3.3.3 were trained for de novo gene prediction using RNA-seq data mapped onto the assemblies [soft-masked with RepeatMasker (*106*)] using HISAT2 (*110*, *111*) v2.1.0 (--dta, --max-intronlen 100000), and ab initio gene predictors were trained using BRAKER (*117*) v3.0.1. In addition, a set of protein sequences obtained by merging the OrthoDB protein dataset for Viridiplantae (odb10; www.orthodb.org) with a high-quality *P. veris* protein set [see (*30*) for details on how the protein dataset was obtained] was used as input for homology-based annotation in BRAKER.

The completeness of the gene annotations was assessed with BUSCO (*96*) v.5.6.1 (-m proteins), using the 2326 single-copy orthologs from the eudicot database (eudicots_odb10; creation date: 8 January 2024). Functional annotation was performed on proteomes with eggNOG-mapper (*118*) v2.1.12 using the eggNOG database (*119*) v5.

### Estimating linkage disequilibrium

To estimate linkage disequilibrium in the regions containing the *S*-locus in *A. vitaliana* and *H. palustris*, we used LDBlockShow (*120*) v1.37 (-MAF 0.2, -Miss 0.9, -SeleVar 2) on the same VCF files used for the population genetic analyses (see above), restricting the analysis to the genomic regions containing the *S*-loci (*A. vitaliana*, chromosome 5: 800,000 to 1,200,000 bp; *H. palustris*, chromosome 9: 31,762,381 to 54,532,255 bp). For *A. vitaliana*, we performed the analysis on the Wallis population VCF without thinning and additionally on the herbarium dataset after applying a thinning step with VCFTools (*121*) v0.1.17 to exclude sites located closer than 100 bp to each other (--thin 100). For *H. palustris*, we applied a 2-kb thinning step (--thin 2000) to exclude sites located closer than 2 kb to each other.

### *S*-locus identification

We identified the *S*-loci of *A. vitaliana* and *H. palustris* using three different approaches. First, we identified morph-specific a *k*-mers and mapped them on the genome assemblies. Second, we used a population genomic approach to searching for regions of high differentiation between individuals of different floral morphs. Both these approaches were adopted using WGS data from 28 individuals for *H. palustris*, while for *A. vitaliana*, these analyses were run separately on both the Wallis and herbarium datasets (see above). In each dataset, pins and thrums were equally represented. Third, we mapped the *P. veris S*-genes on the genome assemblies of *H. palustris* and *A. vitaliana* to test whether homologs of these genes were contained in the newly identified *S*-loci.

#### *Identification of morph-specific* k*-mers*

To identify the *S*-locus in *H. palustris* and *A. vitaliana*, we used a *k*-mer–based approach to detecting morph-specific *k*-mers. This method was chosen because it does not rely on prior knowledge of morph genotypes and is sensitive to both single-nucleotide polymorphisms (SNPs) and structural variation. First, 31-mers were counted in each sample using Jellyfish count (*71*) v2.2.10. The resulting *k*-mers were filtered to retain only those with coverage between 2 and 240, thereby excluding *k*-mers likely arising from sequencing errors (low coverage) or repetitive regions (high coverage). In addition, *k*-mers were retained only if they occurred in at least two samples. Last, a *k*-mer was defined as morph-specific if it was present in *n* − 2 samples of the corresponding morph (where 𝑛 is the total number of samples for that morph) and in none of the samples of the other morph. Morph-specific *k*-mers identified in *H. palustris* and *A. vitaliana* were then mapped on the thrum haplotype assembly of the respective species with BWA-MEM (*81*) v0.7.17. The resulting BAM files were sorted with SAMtools (*122*) v1.9-63, and coverage was calculated in 5-kb windows with Mosdepth (*123*) (--by 5000, --no-per-base).

#### Population genomics analyses

To complement the approach based on morph-specific *k*-mers aimed at identifying the *S*-locus of *A. vitaliana* and *H. palustris* (see above), we searched for genomic regions characterized by high divergence between pin and thrum samples and by a higher heterozygosity in thrums compared to pins. For both species, reads were aligned to the chromosome-scale scaffolds of the thrum-specific haplotype with BWA-MEM (*81*) v0.7.17. The resulting BAM files were sorted with SAMtools (*122*) v1.9-63, and duplicate reads were removed with the Picard MarkDuplicates v2.18.14 (http://broadinstitute.github.io/picard/) (REMOVE_DUPLICATES = true, ASSUME_SORTED = true, VALIDATION_STRINGENCY = SILENT). Variant calling was performed with BCFtools mpileup and call (*124*) v1.8. The resulting VCF files were filtered with VCFtools (*121*) v0.1.17 to keep only sites that represented biallelic SNPs, with a minimum quality of 30, present in at least 25% of samples (--remove-indels, --max-missing 0.25, --minQ 30, --max-alleles 2), and with a sequencing coverage between 5 and 70 for *H. palustris* (--min-meanDP 5, --max-meanDP 70, --min-meanDP 5, --max-meanDP 70) and between 2 and 70 for *A. vitaliana* (--min-meanDP 2, --max-meanDP 70, --min-meanDP 2, --max-meanDP 70). Fixation index (*F*_ST_) was calculated between pins and thrums in 5-kb nonoverlapping windows using *popgenWindows.py* (https://github.com/simonhmartin/genomics_general), allowing for a minimum of 100 sites per window (-w 5000, -m 100, --writeFailedWindows). To compare heterozygosity between pins and thrums, we first estimated heterozygosity in 5-kb nonoverlapping windows for each individual using, allowing for a minimum of 100 sites per window (-w 5000, -m 100, --writeFailedWindows, --analysis indHet). Second, we calculated the mean heterozygosity for pins and for thrums for each window. Last, we subtracted the average heterozygosity in pins from the average heterozygosity in thrums for each window.

#### *Mapping of* Primula S*-genes*

To determine whether the *S*-loci of *H. palustris* and *A. vitaliana* evolved independently from the *P. veris S*-locus, we investigated whether these newly identified *S*-loci contained homologs of the *P. veris S*-genes. We therefore mapped the amino acid sequences of the *P. veris S*-genes (*27*) on the *H. palustris* and *A. vitaliana* proteomes using BLASTp (*82*) (-evalue 1e-5).

To confirm that no misassemblies were present in the newly identified *S*-loci, we mapped the *S*-genes of *H. palustris* (*n* = 115; *S*-alleles) and *A. vitaliana* (*n* = 3; *S*-alleles) to the respective draft assemblies (i.e., before Hi-C scaffolding) using BLASTn (*82*) (-evalue 1e-5). In both cases, all *S*-genes mapped to a single contig: contig “h2tg000008l” of *H. palustris* (60.76 Mb) and contig “2035” of *A. vitaliana* (140 kb). For the latter, see also the “Genome assembly” section.

The final coordinates of the *S*-loci are as follows: In *A. vitaliana*, the *s*-haplotype is located at aviP_sc1: 15,984,083 to 16,018,688 bp, and the *S*-haplotype is located at aviT_sc5: 984,089 to 1,039,657 bp; in *H. palustris*, the *s*-haplotype is located at Hpal_hap1_9: 36,917,167 to 53,577,764 bp, and the *S*-haplotype is located at Hpal_hap2_9: 36,762,381 to 49,532,255 bp; in *H. inflata*, the region syntenic to the *S*-locus is located at Hinf010: 26,847,990 to 37,146,126 bp.

### Identification of haplotype-specific genes in *Hottonia*

To determine whether a gene in the *H. palustris S*-locus was specific to the *S*- or *s*-haplotype or present in both haplotypes, we examined the sequencing coverage across the *S*-locus (normalized to the mean coverage of chromosome 9) across 28 individuals (14 pins and 14 thrums). Because *S*-haplotype–specific genes are expected to be hemizygous in thrums and absent from pins, whereas *s*-specific genes should be hemizygous in thrums and present in both haplotypes in pins, we classified genes as (i) *S*-haplotype specific if they were annotated only on the *S*-haplotype and had a normalized coverage of 0 to 0.2 in pins and 0.3 to 0.7 in thrums; (ii) *s*-haplotype–specific if they were annotated only on the *s*-haplotype and had a normalized coverage of 0.8 to 1.2 in pins and 0.3 to 0.7 in thrums. Using these criteria, 87 genes were shared between haplotypes, 22 were consistently *S*-haplotype specific, and 25 were consistently *s*-haplotype specific. In addition, six genes on the *S*-haplotype and eight on the *s*-haplotype appeared to be haplotype specific only in some samples.

To clarify whether *HiKFB* and *HiPUM* were present in both haplotypes of *H. inflata*, we estimated the sequencing coverage of the *H. inflata* genomic region syntenic to the *H. palustris S*-locus: First, we aligned the *H. inflata* PacBio HiFi reads on the *H. inflata* assembly using minimap v2.24 (*125*) and sorted the output with SAMtools *sort* v1.9-63. Then, a VCF file was created using BCFtools mpileup and call (*124*) v1.8 and filtered using VCFtools (*121*) to keep biallelic sites with quality above 30 and depth above 10 that were not contained in repetitive regions (--minQ 30, --min-meanDP 10, --max-alleles 2, --exclude-bed). The coding DNA sequence (CDS) of each gene were then annotated in the filtered VCF using BCFtools. The annotated VCF was imported in R v4.3.3 (www.R-project.org/), and the presite coverage was extracted using vcfR read.vcfR and extract.gt (*126*). Mean per-gene coverage was calculated using the “aggregate” function in R v4.3.3.

### Differential gene expression analyses

To quantify gene expression in *A. vitaliana*, we used 36 RNA-seq samples comprising leaves and flowers from nine thrum and nine pin individuals and a gene set created by concatenating the CDS of the pin and thrum haplotypes, containing a total of 102,726 CDS. Reads were first trimmed with Trimmomatic (*127*) v0.38, with the parameters recommended by Trinity (*114*) (ILLUMINACLIP: 2:30:10, LEADING: 5, TRAILING: 5, SLIDINGWINDOW: 4:5, MINLEN: 25). The CDS file was indexed with Salmon index (*128*) v1.4.0. Salmon quant was then used to quantify gene expression (--gcBias --validateMappings). Read counts obtained with Salmon for leaf and flower samples were imported separately into R v4.3.3 (www.R-project.org/) using tximport (*129*), and a DESeqDataSet was created with the “DESeqDataSetFromTximport” function of the DESeq2 (*130*) v1.42.1 R/Bioconductor (*131*) package. Read counts were normalized using the default median of ratios method (*130*) and plotted for the *s*- and *S*-alleles of *AvCSE*, *AvEH*, and *AvCYP*. The same workflow was applied to *H. palustris* (four pin samples: three floral and one leaf; four thrum samples: three floral and one leaf) using the CDS derived from the thrum assembly and to *H. inflata* (three floral and three vegetative samples).

### Synteny analyses

Chromosome-scale assemblies from eight Primulaceae species were used for synteny analyses; these included the five species whose genomes were assembled in this study, *P. veris*, *P. edelbergii*, and *A. corniculatum* (*27*, *30*, *132*). The latter species was selected as an outgroup because it is the only Primulaceae species with a chromosome-scale genome assembly (genome size = 903.07 Mb; N50 = 37.74 Mb; L50 = 11) that lies outside the clade comprising *Primula*, *Hottonia*, and *Androsace* and therefore represents the closest available lineage representing the ancestral nondistylous state. Syntenic genes were identified within each species and between each species pair using the MCScan tool of the JCVI toolkit (*133*, *134*) v1.3.6, following the GitHub manual [github.com/tanghaibao/jcvi/wiki/MCscan-(Python-version)]. First, homologous genes were identified with jcvi.compara.catalog ortholog (--min_size=5, --dist=20, --no_strip_names), and the resulting anchor files were used to generate whole-genome dot plots with *jcvi.graphics.dotplot*. Then, simplified anchor files were generated with jcvi.compara.synteny screen (--minspan=30, --simple) and used to create whole-genome macrosynteny plots with *jcvi.graphics.karyotype*. Last, we generated a list of syntenic genes with jcvi.compara.synteny mcscan (--iter=1), which was used to (i) generate microsynteny plots, (ii) calculate *d*_S_ between syntenic genes within and between species to infer WGDs in *Androsace*, and (iii) estimate *d*_S_ between the *S*- and *s*-haplotype of *H. palustris* and *A. vitaliana*.

### Identification of WGDs in *Androsace*

To identify WGDs in *Androsace* species, we estimated *d*_S_ between syntenic genes within and between *A. vitaliana* (thrum haplotype; 2*n* = 4*x* = 40), *A. wulfeniana* (2*n* = 4*x* = 40), and *A. septentrionalis* (2*n* = 2*x* = 20). For each estimate, syntenic genes were identified using MCScanX as outlined above. Then, *d*_S_ values were estimated using ParaAT (*135*) v2.0, which uses MUSCLE (*136*) v3.8.31 to align sequences and KaKs_Calculator (*137*) v2.0 to calculate *d*_S_. The resulting *d*_S_ distributions were then plotted in R v4.3.3 using the ggplot2 package. In addition, we estimated the syntenic depth within and between the abovementioned *Androsace* genomes using MCScanX jcvi.compara.synteny depth.

### Testing for TE enrichment in *S*-loci

To test whether the observed difference in TE content between the *S*- and *s*-haplotypes of *A. vitaliana* and *H. palustris* was significantly higher than the genomic background, we implemented the following procedure. For *A. vitaliana*, we identified all three-gene syntenic windows between the pin and thrum haploid assemblies, estimated TE abundance within each window, and calculated the ratio of TE abundance for each “thrum window” over its syntenic “pin window.” A null distribution was generated from the 32,872 ratios obtained, and a statistical test was performed by converting both the observed *S*-locus ratio and all null ratios to their absolute deviation from 1 and computing an empirical two-sided *P* value as the proportion of null deviations greater than or equal to the observed deviation in R v4.3.3 (www.R-project.org/). For *H. palustris*, the same procedure was applied using 115-gene windows, reflecting the size of its *S*-haplotype, and the null distribution was made using the 19,187 ratios obtained.

### Analyses on *S*-locus expansion in *H. palustris*

To test whether the large size of the *S*-locus in *H. palustris* was explained by an expansion of recombination suppression in *H. palustris* or the *S*-locus was originally larger and “shrank” in *Primula*, we estimated *d*_S_ between syntenic orthologs of *H. palustris–P. veris* (*n* = 15,964), *H. palustris–H. inflata* (*n* = 18,830), and between the *S*- and *s*-haplotype of *H. palustris* (*n* = 88); orthologous gene pairs and allele pairs were identified with MCScanX as outlined above, and *d*_S_ values were estimated using ParaAT (*135*) v2.0. To search for evolutionary strata in the *S*-locus of *H. palustris*, we plotted the *d*_S_ values obtained between the *S*- and *s*-alleles for the 87 *S*-genes present in both haplotypes, ordering genes by their position on the *s*-haplotype.

### Construction of gene families for phylogenetic analysis

Before performing phylogenetic analysis, we clustered genes into gene families (orthogroups) using OrthoFinder (*138*) v2.3.11 run with default parameters on 22 proteomes. These proteomes included seven proteomes presented here [*A. vitaliana* (pin haplotype), *A. vitaliana* (thrum haplotype), *A. septentrionalis*, *A. wulfeniana*, *H. inflata*, *H. palustris* (pin haplotype), and *H. palustris* (thrum haplotype)]; proteomes from 10 Ericales species, representing 7 of the 22 Ericales families (*139*), selected for having chromosome-scale genome assemblies [*Actinidia chinensis* (*140*), *A. corniculatum* (*132*), *C. sinensis* (*141*), *Dyospiros oleifera* (*142*), *Gilia yorkii* (*143*), *P. edelbergii* (*30*), *P. veris* (*27*), *Rhododendron henanense* (*144*), *Vaccinium darrowii* (*145*), and *Vitellaria paradoxa* (*146*)]; proteomes from five additional species, selected for having high-quality genome assemblies and gene annotations and being widespread across the angiosperm phylogeny [*Amborella trichopoda* (*147*), *Arabidopsis thaliana* (*148*), *S. lycopersicum* (*149*), *Oryza sativa* (*150*), and *Vitis vinifera* (*151*)]. The final dataset contained 733,259 total proteins, 674,132 (91.9%) of which were assigned to 40,194 orthogroups (table S20).

The *S*-genes were contained in the following orthogroups: OG0006534 (*PvCCM*), containing 32 genes; OG0003769 (*PvGLO*), containing 43 genes; OG0000663 (*PvCYP*), containing 92 genes; OG0001331 (*PvPUM*), containing 70 genes; OG0000291 (*PvKFB*), containing 127 genes; OG0008416 (*AvCSE*), containing 28 genes; and OG0005520 (*AvEH*), containing 35 genes. To avoid that some *S*-gene-containing orthogroups contained too few genes, thus hindering the phylogenetic analysis, we created “extended orthogroups” by aligning the amino acid sequences of *P. veris* and *A. vitaliana S*-genes against all orthogroup sequences with BLASTp (*147*) (-evalue 1e-25, -max_target_seqs 3) and concatenating the orthogroups having at least one match. The following orthogroups were concatenated: OG0002583, OG0003881, OG0005521, OG0007367, OG0008416, and OG0011811 for *AvCSE* (212 genes); OG0000010, OG0000663, OG0003314, OG0005243, and OG0013733 for *PvCYP* (619 genes); OG0005129, OG0005520, OG0008234, OG0017032, OG0035311, and OG0037733 for *PvPUM* (110 genes); OG0002710, OG0003769, OG0010238, and OG0011028 for *PvGLO* (143 genes); and OG0001331, OG0002862,OG0009601, and OG0014179 for *PvEH* (160 genes). No additional orthogroups were found to contain homologs of *PvCCM* and *PvKFB*, other than OG0006534 and OG0000291, respectively. The sequences contained in these extended orthogroups were then used to generate gene phylogenies (see below).

### Phylogenetic analysis

The *S*-gene homologs obtained from genomes and contained in the extended orthogroups were then used to infer phylogenetic relationships within each gene family. The *PvCCM* homologs obtained from genomes were aligned using MAFFT (*152*) v7.450 Auto algorithm. A preliminary phylogeny was estimated with FastTree (*153*) v2.1.11 using a GTR + G (four categories) model. This analysis revealed no clear evidence of subdivision into several gene lineages, so all the sequences were aligned with OMM_MACSE (*154*, *155*) v12.01 for use in phylogenetic and dating analyses.

*AvCSE* is part of the larger class I carboxylesterase gene family, which also contains tannase and acetate esterase genes (*156*). One sequence of each of the three subfamilies was obtained from GenBank to serve as references (*CSE* from *Arabidopsis*, tannase from *Camellia*, and acetate esterase from *Solanum*). These reference sequences were aligned alongside *AvCSE* homologs obtained from genomes, using MAFFT v7.450 Auto algorithm. A preliminary phylogeny was estimated with FastTree v2.1.11 using a GTR + G (four categories) model. This analysis revealed that *CSE*, tannase, and acetate esterase formed three separate lineages, in addition to six other lineages of class I carboxylesterase genes. We kept only the sequences of the *CSE* lineage, which included the *Androsace S*-locus sequence, while all other sequences were discarded.

Additional mRNA sequences from the *CYP72* clan (*CYP72*, *CYP709*, *CYP735*, *CYP734*, *CYP715*, *CYP721*, *CYP714*, and *CYP749* families), the *CYP86* clan (*CYP704*, *CYP94*, *CYP86*, and *CYP96* families), and the *CYP87* clan (*CYP87* family) of *A. thaliana* and *O. sativa* were obtained from GenBank or European Molecular Biology Laboratory (EMBL), using the previously published guide trees and classification (*157*, *158*). These reference sequences were aligned alongside *CYP* homologs obtained from genomes, using MAFFT v7.450 Auto algorithm. A preliminary phylogeny was estimated with FastTree v2.1.11 using a GTR + G (four categories) model. Then, a subset of *CYP* homologs was selected to include only genes in the *CYP734* family, discarding all other sequences, except for reference sequences obtained directly from GenBank and EMBL, which were kept as outgroups. The reduced dataset was realigned with OMM_MACSE v12.01 for use in phylogenetic and dating analyses.

The *AvEH* homologs obtained from genomes were aligned using MAFFT v7.450 Auto algorithm. A preliminary phylogeny was estimated with FastTree v2.1.11 using a GTR + G (four categories) model. This analysis revealed a division into three distinct gene lineages, each encompassing sequences from all analyzed species, consistent with the presence of three separate *EH* gene subfamilies. Sequences from the two lineages that did not include the *Androsace S*-locus sequence were excluded. The retained sequences were realigned with OMM_MACSE v12.01 for use in phylogenetic and dating analyses.

Additional reference sequences of *GLO*/*PI* and *AP3*/*TM6*/*DEF* genes of *A. thaliana* and *S. lycopersicum* were obtained from GenBank. These reference sequences were aligned alongside *GLO* homologs obtained from genomes, using MAFFT v7.450 Auto algorithm. A preliminary phylogeny was estimated with FastTree v2.1.11 using a GTR + G (four categories) model. Then, a subset of homologs was selected to include only *GLO*/*PI* and *AP3*/*TM6*/*DEF* genes, discarding all other sequences. The reduced dataset was realigned with OMM_MACSE v12.01 for use in phylogenetic and dating analyses.

The *PvKFB* homologs obtained from genomes were aligned using MAFFT v7.450 Auto algorithm. A preliminary phylogeny was estimated with FastTree v2.1.11 using a GTR + G (four categories) model. The phylogeny revealed an intricate history of lineage-specific duplications, as previously shown (*159*). However, the clade containing *S*-locus sequences of *Hottonia* and *Primula* contained other Ericales sequences placed according to known interspecific relationships. This clade was sister to another clade containing a similar set of Ericales species, suggesting an ancient duplication event shared across Ericales. These two sister clades were selected for further analysis, discarding all other sequences.

Additional mRNA sequences representing the diversity of plant *PUM*/*PUF* genes were obtained from GenBank, using a previously published guide tree and classification (*160*). These reference sequences were aligned alongside *PvPUM* homologs obtained from genomes, using MAFFT v7.450 Auto algorithm. A preliminary *PUM* phylogeny was estimated with FastTree v2.1.11 using a GTR + G (four categories) model. This analysis showed *S*-locus sequences to be nested within one of two sister subclades of *PUM* Clade II as previously identified (*160*), this clade showing evidence of a deep duplication shared across all flowering plants. Only the Clade II subclade including the *S*-locus sequences was retained for further analysis, discarding all other sequences.

Protein-coding gene alignments were generated using OMM_MACSE v12.01, which ensures frame-preserving alignments while accounting for insertions, deletions, and sequencing errors. Each gene was aligned independently, using the standard genetic code (code 1) and disabling pre- and postfiltering to ensure that all sequences and nucleotides were retained in the alignments. The dataset was partitioned by codon and the best models, and partitioning scheme for each gene alignment was selected by Bayesian Information Criterion (BIC) in PartitionFinder (*161*) v2.1.1.

Nonultrametric phylogenetic trees were estimated with Bayesian inference in MrBayes (*162*) v.3.2.7a using the best models and partitions to avoid the constraints of molecular clock models. Each gene alignment was analyzed with two independent Markov chain Monte Carlo (MCMC) runs of 10 million generations, sampling every 2000 iterations, with convergence assessed in Tracer (*163*) v.1.7.2, discarding 20% of each run as burnin. The resulting posterior trees were then used as input for AleRax (*164*) to infer reconciled gene trees on the species tree without introducing biases from clock model assumptions at this stage.

For divergence time dating, separate BEAST (*165*) v2.7.7 analyses were done on each gene family using the optimal partitioning schemes and models selected by PartitionFinder, while keeping clock and tree models linked within each gene family. We used a Yule tree prior, with a log-normal prior on birth rate with an SD of 1.175 (to create a 95% highest probability density of about two orders of magnitude around mean) and a mean calculated according to the formula lambda = ln(*n*/2)/*t*, where *n* is the number of sequences in the alignment and *t* is the estimated root age. A root age of 139 Ma was used for angiosperms according to (*166*), and *n* was estimated by counting the number of sequences in the largest subclade of each gene family phylogeny that contained a single copy of *Amborella*. Thus, an average birth rate prior was set to 0.0199 for *CCM*, 0.0268 for *CFB*, 0.0190 for *CSE*, 0.0270 for *CYP*, 0.0225 for *EH*, 0.0233 for *GLO*, 0.0327 for *KFB*, and 0.0246 for *PUM*. An optimized relaxed clock model was implemented with a log-normal prior on the rate parameter (ORCucldMean), with a mean of 0.00615 substitution/Myr as previously calculated in *P. veris* (*26*) and an SD of 0.23 (giving a 95% highest probability density on the mean of 0.00382 to 0.00940 substitutions/Myr) based on the rate variation in other plants, as previously reported (*167*).

Uniform fossil calibrations were assigned on each gene family phylogeny whenever the phylogenetic relationships estimated by AleRax allowed unambiguous determination that a splitting event was due to speciation, rather than gene duplication within one species. The MRCA constraint in BEAST analyses was enforced as monophyletic only if it received ≥95% support in the AleRax gene phylogeny reconciliation analysis. The minimum bound of age calibrations corresponded to the age of the fossil, and the maximum was set to 140 Ma, which is the maximum bound for angiosperms estimated in (*166*) using the bracketing method described in (*168*). Stem dates were used instead of crown dates for the minimum calibration of Primulaceae and *Primula* because the species available in our analyses were nested within Primulaceae and *Primula*, and calibrating the crown of the clade recovered in our analyses would thus have overestimated its age. The fossil calibrations we used were (i) crown age of angiosperms: 136 to 140 Ma [angiosperm pollen fossil cited in (*166*)]; (ii) stem age of eudicotyledons: 125 to 140 Ma [tricolpate pollen fossil cited in (*166*)]; (iii) crown age of Ericales: 89 to 140 Ma [flower fossil cited in (*166*)]; (iv) stem age of Primulaceae: 66 to 140 Ma [flower fossil cited in (*169*)]; and (v) stem age of *Primula*: 16 to 140 Ma [seed fossil cited in (*170*)]. Two separate BEAST dating analyses were run of 50 million generations for each gene, using coupled MCMC with three heated chains. Results were checked in Tracer v.1.7.2 to ensure convergence of the chains and effective sample sizes of >200 for each parameter after discarding 20% of each run as burnin. Maximum clade credibility trees were constructed from the resulting trace files with LogCombiner.
