# Supplementary material for: Distinct genomic architectures but the same gene underlie the convergent evolution of a plant supergene
Source: Sci Adv. 2026 Jun 10;12(24):eaec1996. doi: 10.1126/sciadv.aec1996 (PMC13251838; doi:10.1126/sciadv.aec1996)
Supplement: Supplementary file 1 — Figs. S1 to S42 Legends for tables S1 to S21 [file sciadv.aec1996_sm.pdf]

Science Advances
AAAS

Supplementary Materials for

Distinct genomic architectures but the same gene underlie the convergent evolution of a plant supergene

Giacomo Potente *et al.*

Corresponding author: Giacomo Potente, giacomo.potente@systbot.uzh.ch;
Narjes Yousefi, narjes.yousefi2@uzh.ch; Étienne Léveillé-Bourret, etienne.leveille-bourret@umontreal.ca;
Elena Conti, elena.conti@systbot.uzh.ch

Sci. Adv. **12**, eaec1996 (2026)
DOI: 10.1126/sciadv.aec1996

The PDF file includes:

Figs. S1 to S42
Legends for tables S1 to S21

Other Supplementary Material for this manuscript includes the following:

Tables S1 to S21

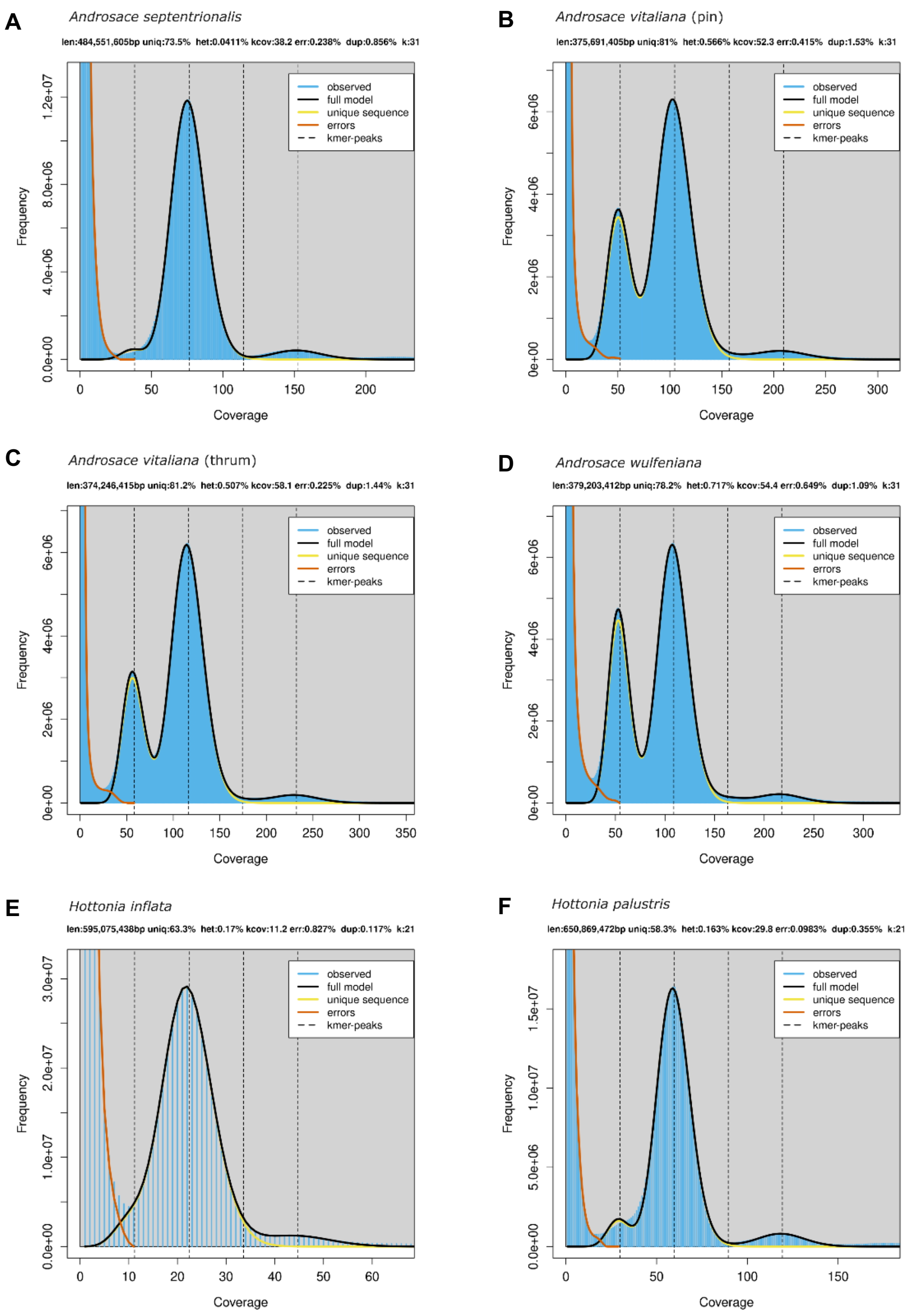

Fig. S1: Genome profiles obtained with GenomeScope.
K-mer density plots were obtained with GenomeScope using Illumina data and 31-mers for *A. septentrionalis* (**A**), *A. vitaliana* (pin and thrum haplotypes; **B** and **C**, respectively), and *A. wulfeniana* (**D**), and using PacBio HiFi data and 21-mers for *H. inflata* (**E**) and *H. palustris* (**F**).

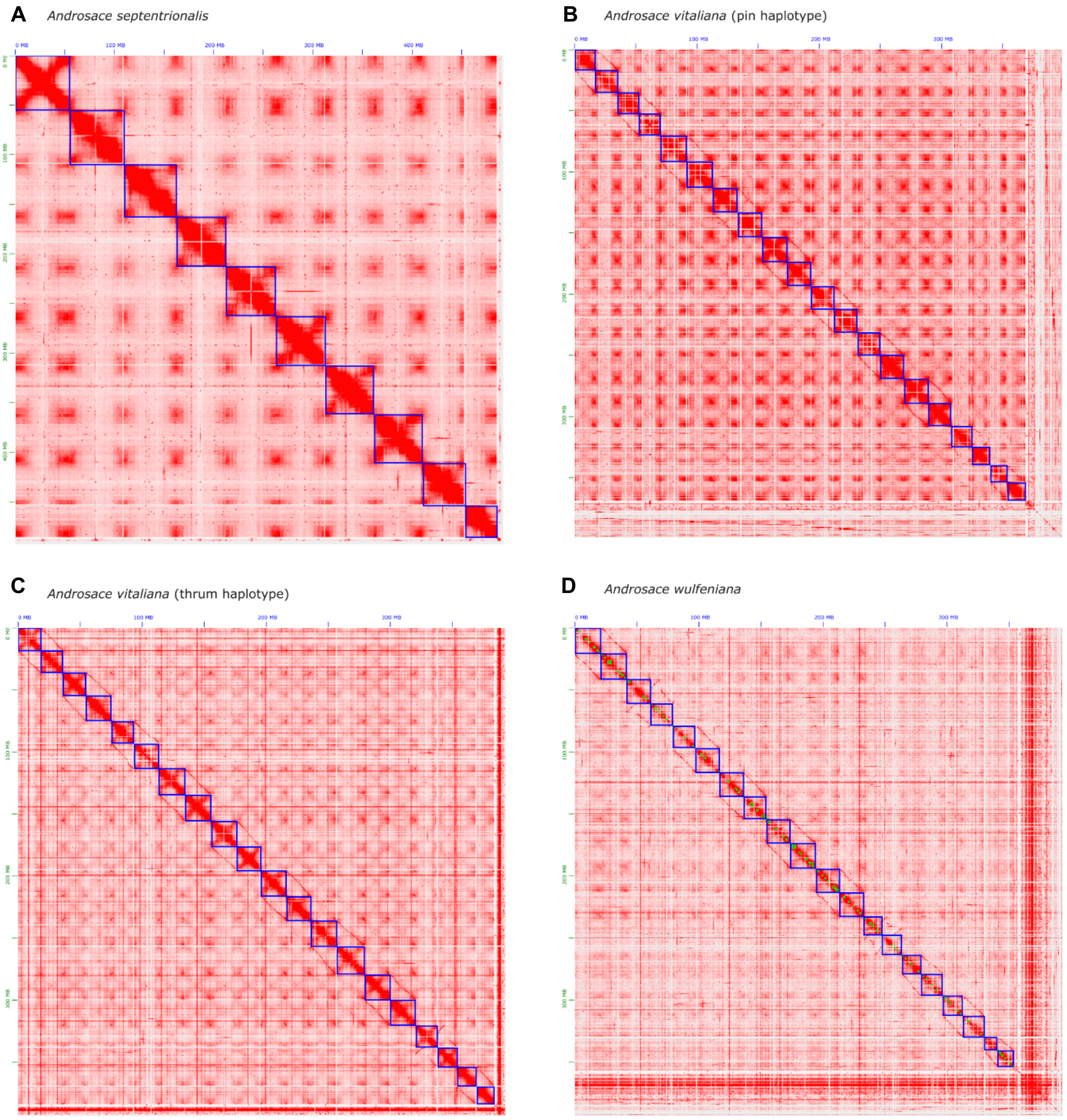
A
Androsace septentrionalis
B
Androsace vitaliana (pin haplotype)
C
Androsace vitaliana (thrum haplotype)
D
Androsace wulfeniana

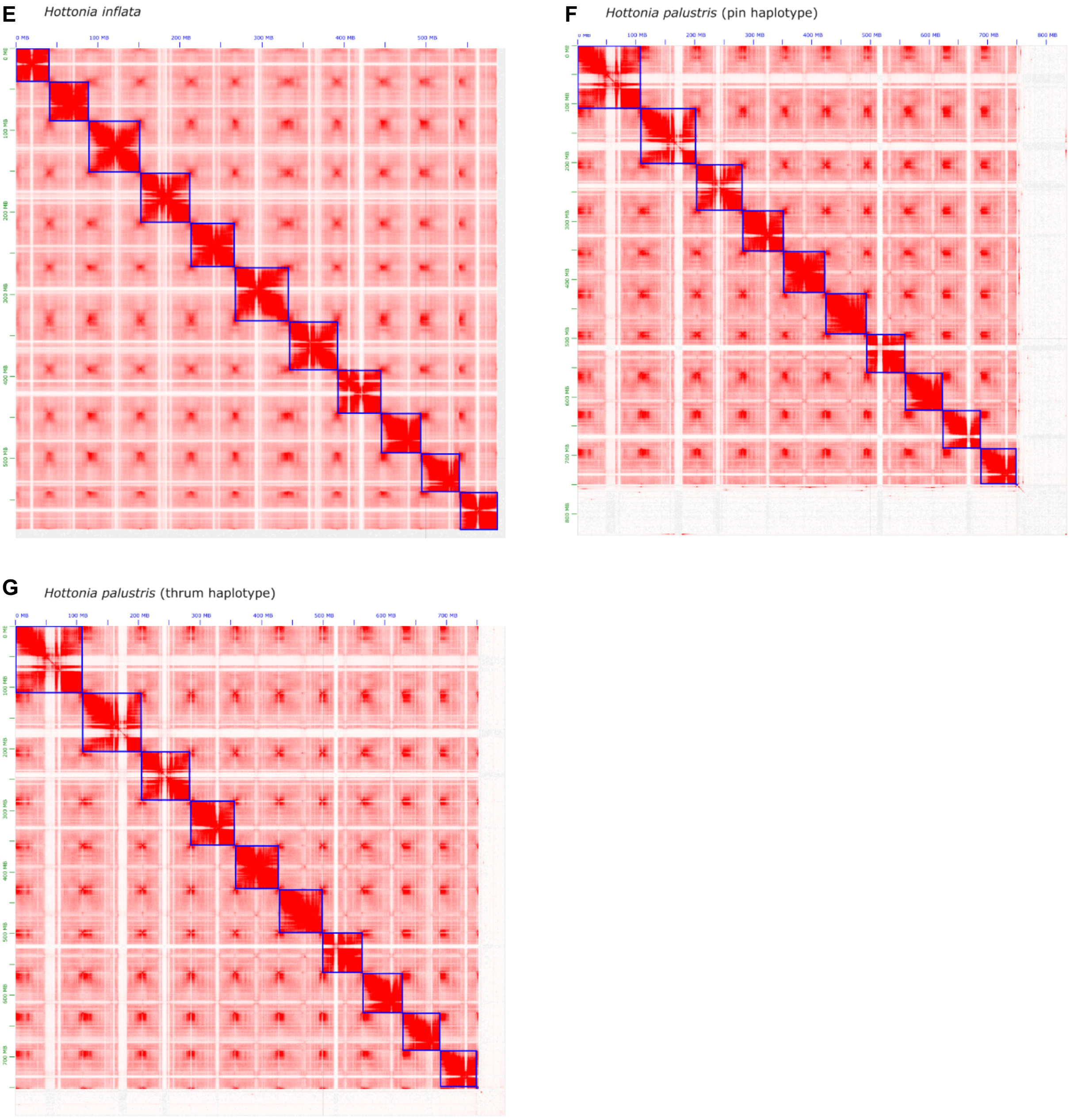

Fig. S2: Hi-C contact maps for the seven genome assemblies presented here.
Contact maps of Hi-C libraries for *A. septentrionalis* (**A**), *A. vitaliana* (pin haplotype; **B**), *A. vitaliana* (thrum haplotype; **C**), *A. wulfeniana* (**D**), *H. inflata* (**E**), *H. palustris* (pin haplotype; **F**), and *H. palustris* (thrum haplotype; **G**). Darker colors indicate higher density of chromatin contacts; chromosome-scale scaffolds are delimited by blue boxes.

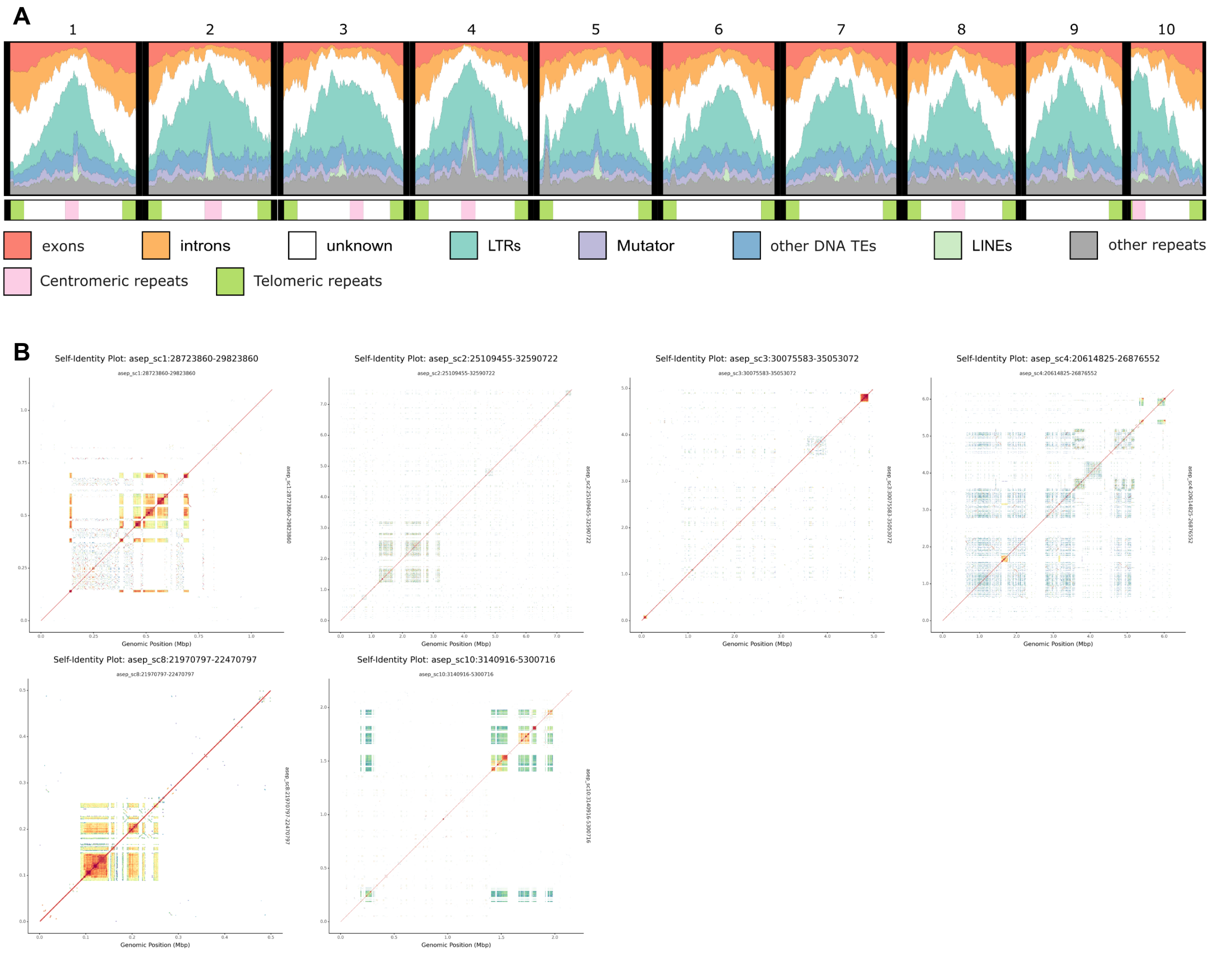

Fig. S3: Overview of the *Androsace septentrionalis* genome assembly.

A. (Top) Distribution of repeat and gene density across the *A. septentrionalis* genome assembly, calculated in sliding windows (2-Mb width, 100-kb steps); the category “other repeats” includes both TEs and tandem repeats. (Bottom) Putative centromeric (pink) and telomeric (green) regions, identified by QuarTet; to ease visualization the minimum size of each plotted block is 6 Mb. Centromeres are characterized by an enrichment of LINEs (light green). **B.** Self-identity heatmap of the centromeric regions identified by QuarTeT (chromosomes 1, 2, 3, 4, 8, 10), generated with ModDotPlot; colors indicate percent sequence identity, following the ModDotPlot default gradient from cool to warm hues (low to high identity).

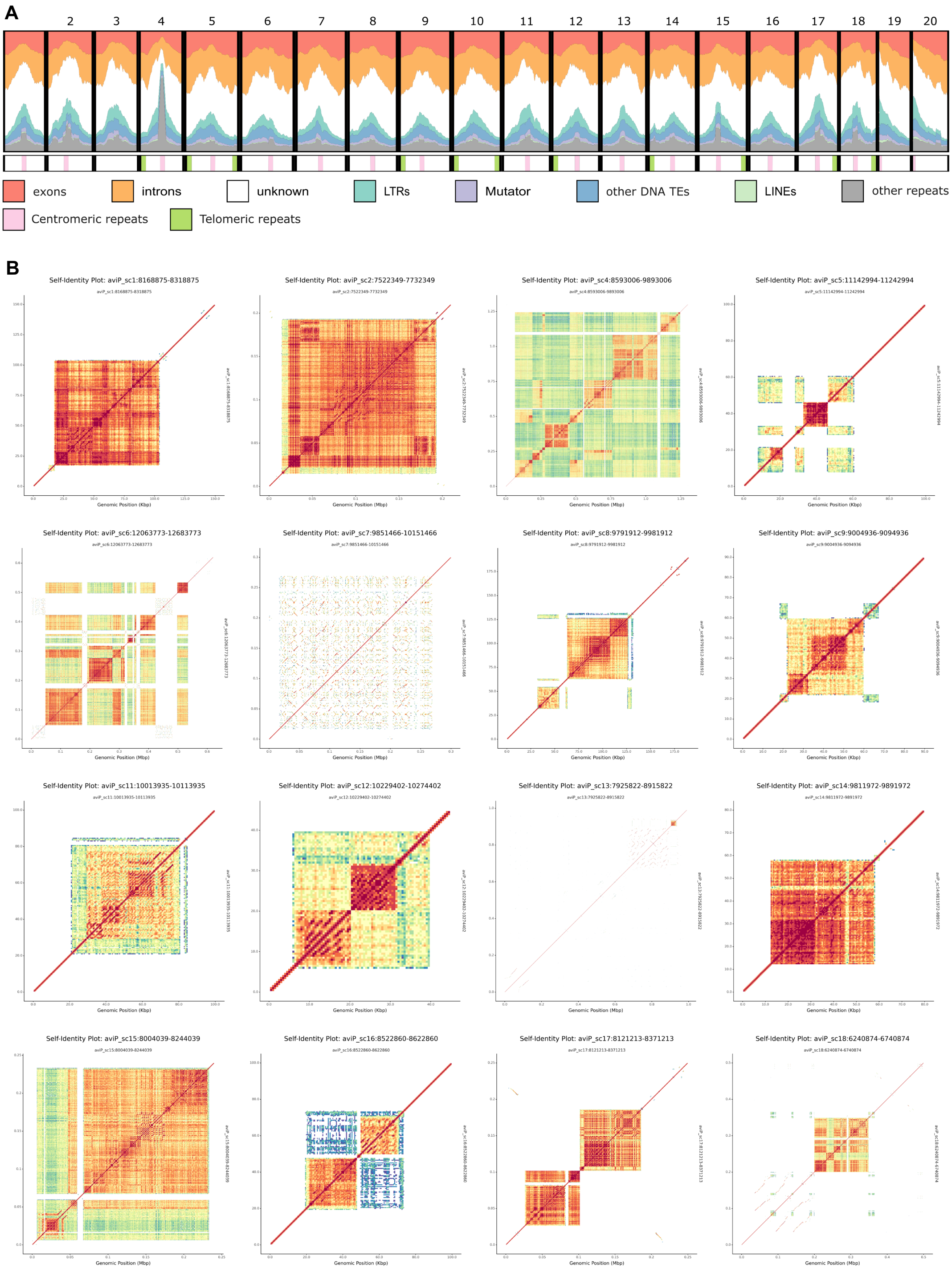
A
2
3
4
5
6
7
8
9
10
11
12
13
14
15
16
17
18
19
20
exons
introns
unknown
LTRs
Mutator
other DNA TEs
LINEs
other repeats
Centromeric repeats
Telomeric repeats
B
Self-Identity Plot: aviP_sc1:8168875-8318875
Self-Identity Plot: aviP_sc2:7522349-7732349
Self-Identity Plot: aviP_sc4:8593006-9893006
Self-Identity Plot: aviP_sc5:11142994-11242994
Self-Identity Plot: aviP_sc6:12063773-12683773
Self-Identity Plot: aviP_sc7:9851466-10151466
Self-Identity Plot: aviP_sc8:9791912-9981912
Self-Identity Plot: aviP_sc9:9004936-9094936
Self-Identity Plot: aviP_sc11:10013935-10113935
Self-Identity Plot: aviP_sc12:10229402-10274402
Self-Identity Plot: aviP_sc13:7925822-8915822
Self-Identity Plot: aviP_sc14:9811972-9891972
Self-Identity Plot: aviP_sc15:8004039-8244039
Self-Identity Plot: aviP_sc16:8522860-8622860
Self-Identity Plot: aviP_sc17:8121213-8371213
Self-Identity Plot: aviP_sc18:6240874-6740874
Genomic Position (Kbp)
Genomic Position (Mbp)

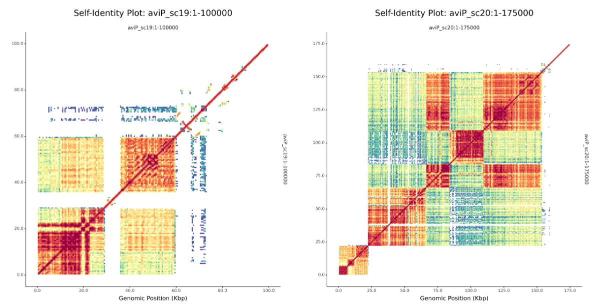

Fig. S4: Overview of the *Androsace vitaliana* genome assembly (pin haplotype).
A. (Top) Distribution of repeat and gene density across the *A. vitaliana* genome assembly (pin haplotype), calculated in sliding windows (2-Mb width, 100-kb steps); the category "other repeats" includes both TEs and tandem repeats. (Bottom) Putative centromeric (pink) and telomeric (green) regions, identified by QuarTet; to ease visualization the minimum size of each plotted block is 2 Mb. Centromeres are characterized by an enrichment of "other repeats" (dark grey). **B.** Self-identity heatmap of the centromeric regions identified by QuarTeT (all chromosomes except 3 and 10), generated with ModDotPlot; colors indicate percent sequence identity, following the ModDotPlot default gradient from cool to warm hues (low to high identity).

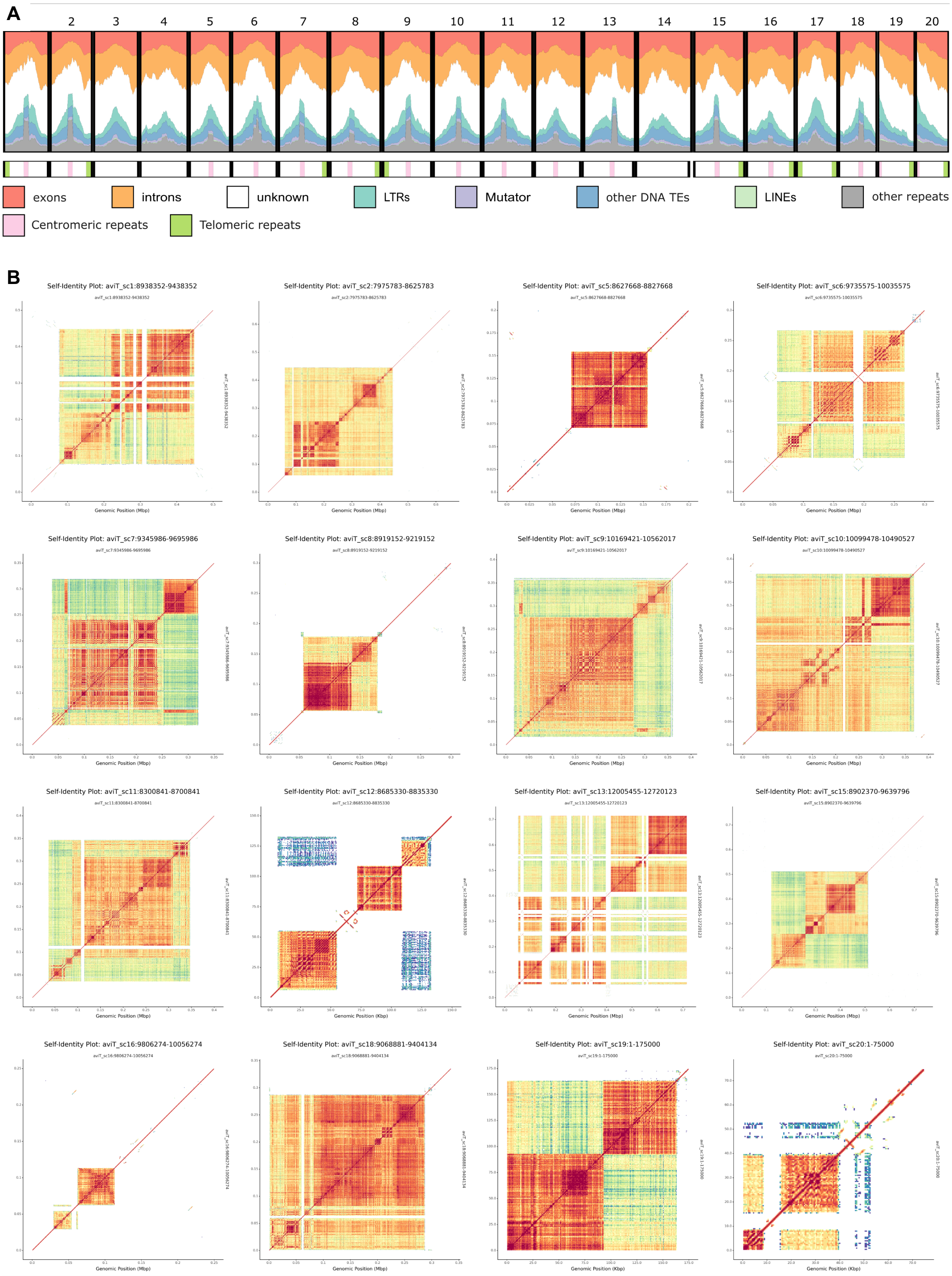

Fig. S5: Overview of the *Androsace vitaliana* genome assembly (thrum haplotype).
A. (Top) Distribution of repeat and gene density across the *A. vitaliana* genome assembly (pin haplotype), calculated in sliding windows (2-Mb width, 100-kb steps); the category "other repeats" includes both TEs and tandem repeats. (Bottom) Putative centromeric (pink) and

telomeric (green) regions, identified by QuarTet; to ease visualization the minimum size of each plotted block is 2 Mb. Centromeres are characterized by an enrichment of “other repeats” (dark grey). **B.** Self-identity heatmap of the centromeric regions identified by QuarTeT (all chromosomes except 3, 4, 14, and 17), generated with ModDotPlot; colors indicate percent sequence identity, following the ModDotPlot default gradient from cool to warm hues (low to high identity).

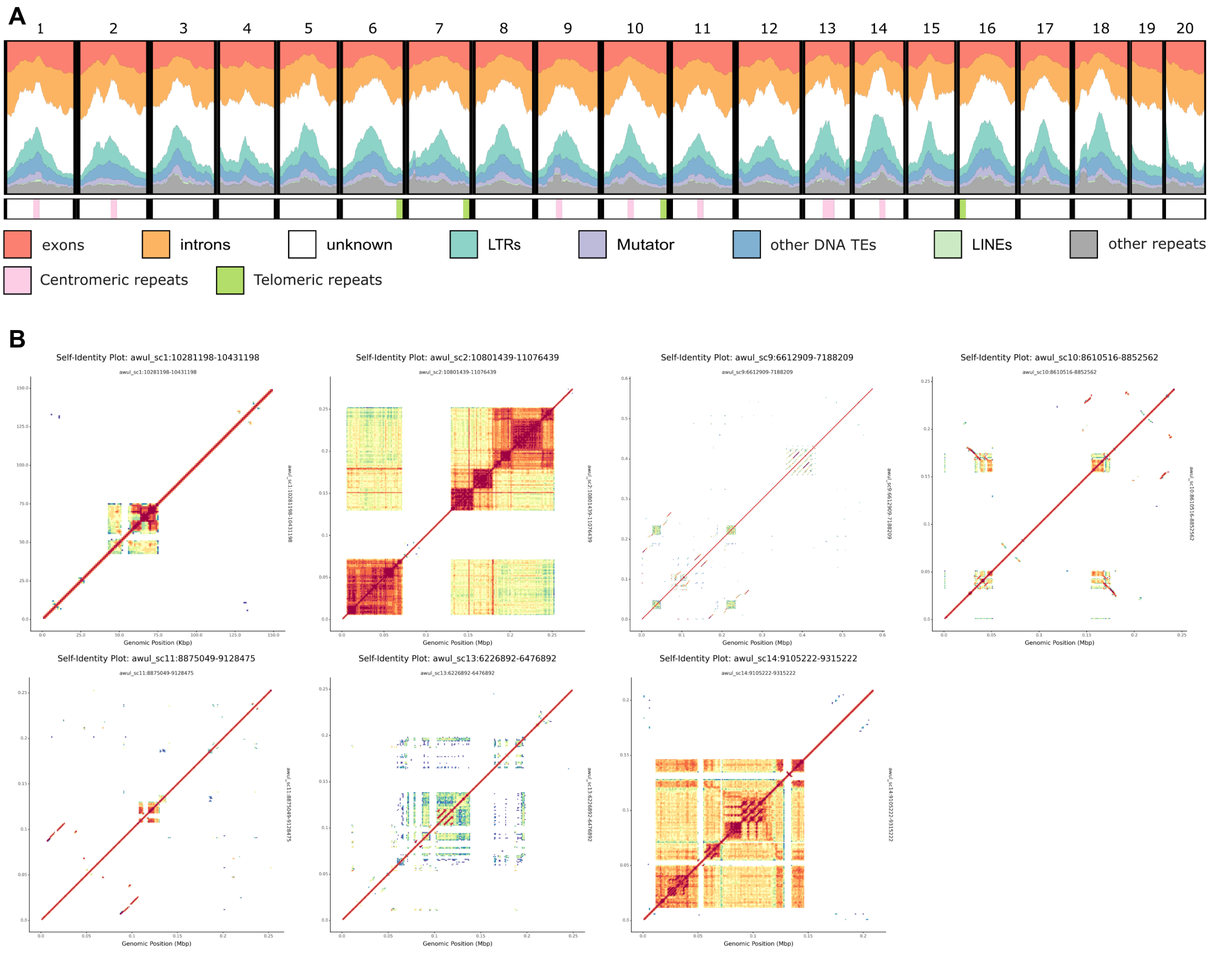

Fig. S6: Overview of the *Androsace wulfeniana* genome assembly.
A. (Top) Distribution of repeat and gene density across the *A. wulfeniana* genome assembly, calculated in sliding windows (2-Mb width, 100-kb steps); the category "other repeats" includes both TEs and tandem repeats. (Bottom) Putative centromeric (pink) and telomeric (green) regions, identified by QuarTet; to ease visualization the minimum size of each plotted block is 2 Mb. Centromeres are characterized by an enrichment of repeats. **B.** Self-identity heatmap of the centromeric regions identified by QuarTeT (chromosomes 1, 2, 9, 10, 11, 13, and 14), generated with ModDotPlot; colors indicate percent sequence identity, following the ModDotPlot default gradient from cool to warm hues (low to high identity).

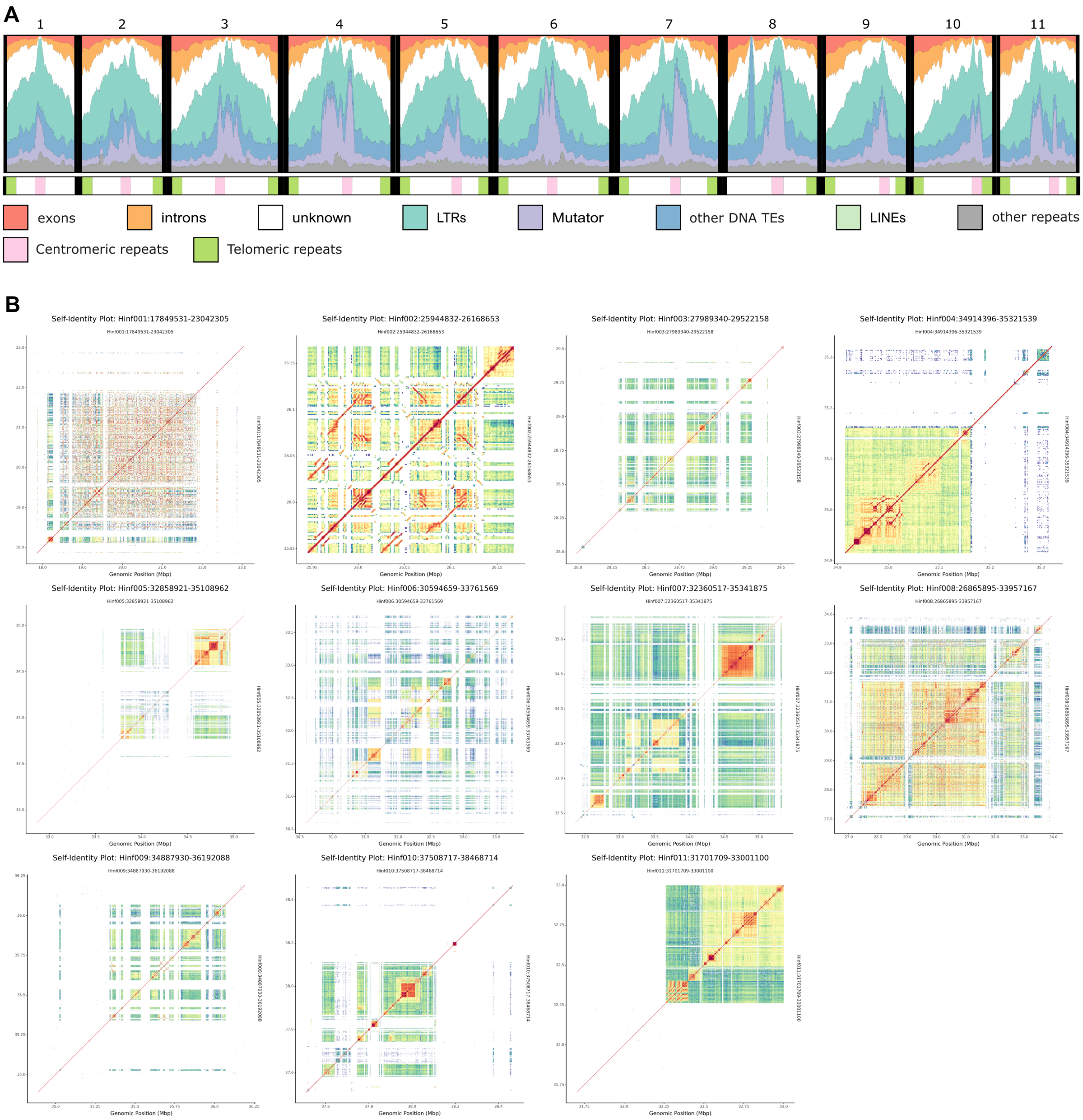

Fig. S7: Overview of the *Hottonia inflata* genome assembly.
A. (Top) Distribution of repeat and gene density across the *H. inflata* genome assembly, calculated in sliding windows (2-Mb width, 100-kb steps); the category "other repeats" includes both TEs and tandem repeats. (Bottom) Putative centromeric (pink) and telomeric (green) regions, identified by QuarTet; to ease visualization the minimum size of each plotted block is 6 Mb. Centromeres are characterized by an enrichment of Mutator TEs (purple). **B.** Self-identity heatmap of the centromeric regions identified by QuarTeT (all chromosomes), generated with ModDotPlot; colors indicate percent sequence identity, following the ModDotPlot default gradient from cool to warm hues (low to high identity).

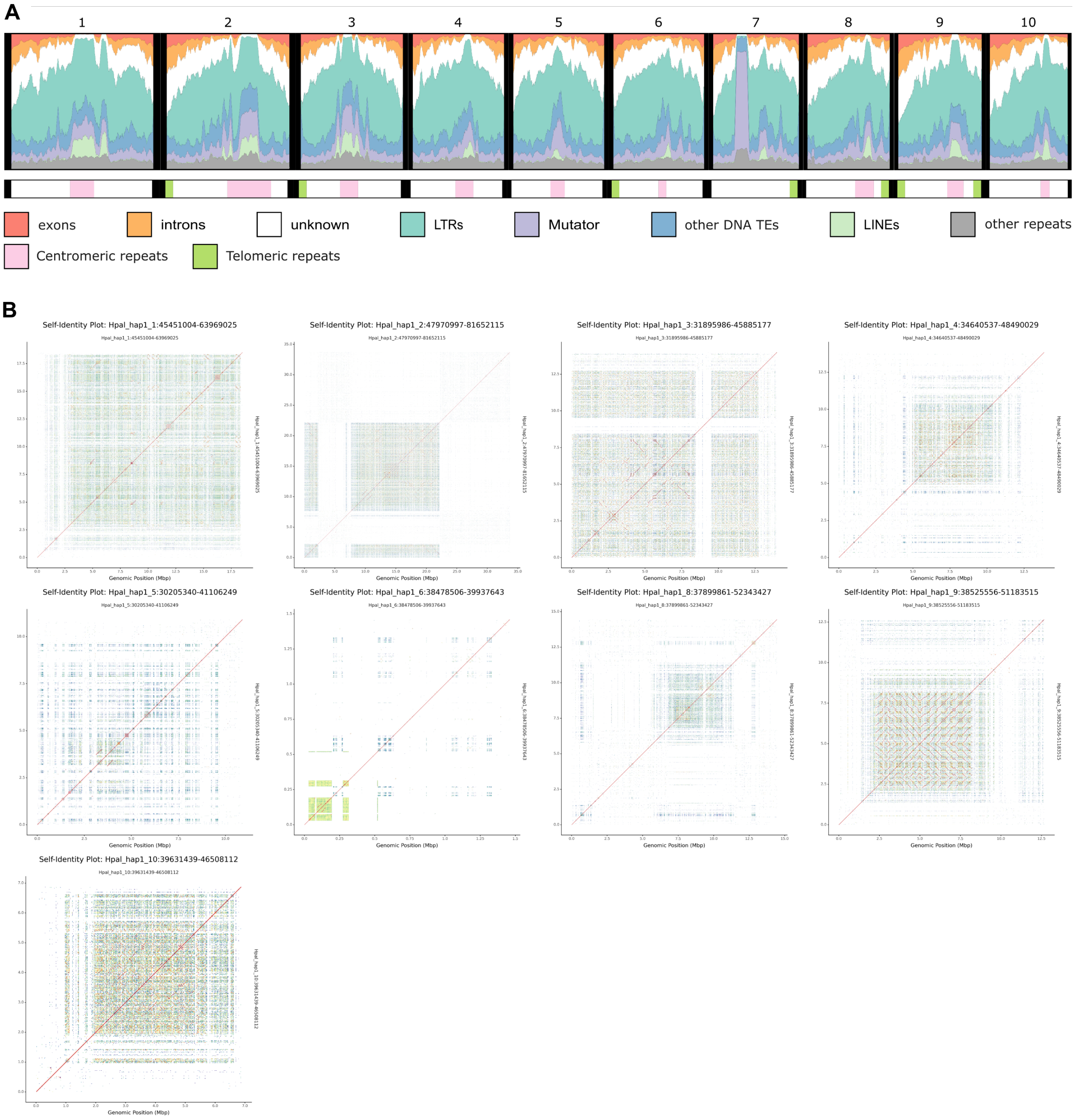

Fig. S8: Overview of the *Hottonia palustris* genome assembly (pin haplotype).
A. (Top) Distribution of repeat and gene density across the *H. palustris* genome assembly (pin haplotype), calculated in sliding windows (2-Mb width, 100-kb steps); the category "other repeats" includes both TEs and tandem repeats. (Bottom) Putative centromeric (pink) and telomeric (green) regions, identified by QuarTet; to ease visualization the minimum size of each plotted block is 6 Mb. Centromeres are characterized by an enrichment of LINEs (light green). **B.** Self-identity heatmap of the centromeric regions identified by QuarTeT (all chromosomes except 7), generated with ModDotPlot; colors indicate percent sequence identity, following the ModDotPlot default gradient from cool to warm hues (low to high identity).

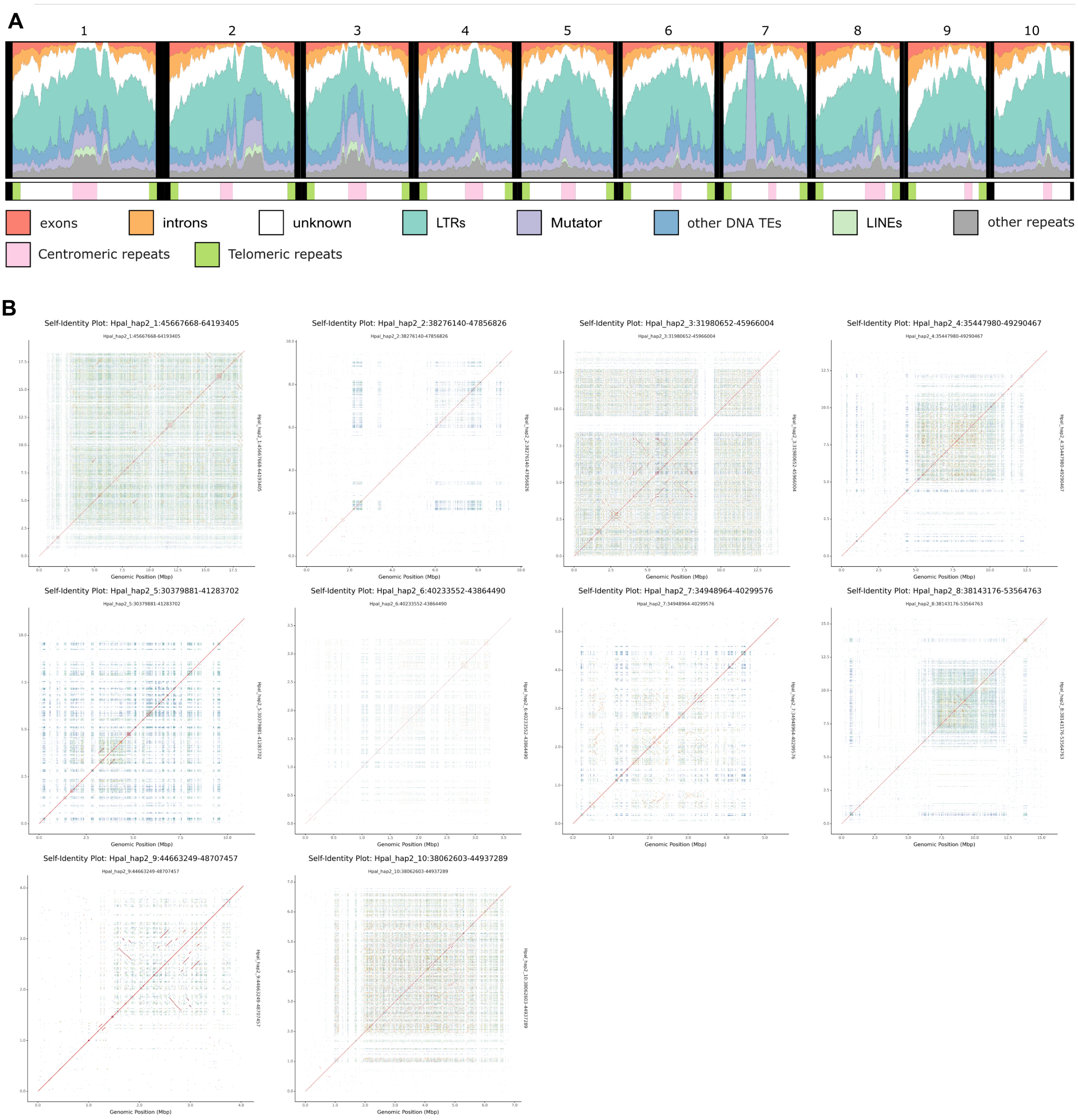

Fig. S9: Overview of the *Hottonia palustris* genome assembly (thrum haplotype).
A. (Top) Distribution of repeat and gene density across the *H. palustris* genome assembly (thrum haplotype), calculated in sliding windows (2-Mb width, 100-kb steps); the category "other repeats" includes both TEs and tandem repeats. (Bottom) Putative centromeric (pink) and telomeric (green) regions, identified by QuarTet; to ease visualization the minimum size of each plotted block is 6 Mb. Centromeres are characterized by an enrichment of LINEs (light green). **B.** Self-identity heatmap of the centromeric regions identified by QuarTeT (all chromosomes), generated with ModDotPlot; colors indicate percent sequence identity, following the ModDotPlot default gradient from cool to warm hues (low to high identity).

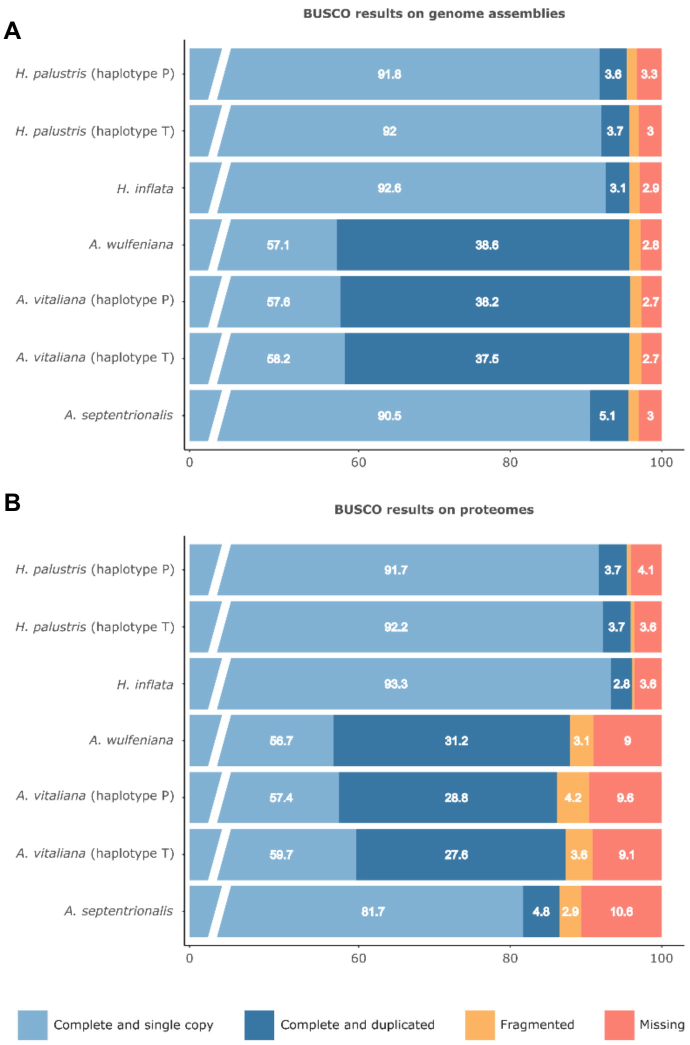

Fig. S10: Genome and proteome evaluation by BUSCO.

Completeness BUSCO scores for the genome assemblies (**A**), and respective proteomes (**B**) presented here, obtained using the eudicots_odb10 database, which contains 2,326 single-copy orthologs. Numbers in each bar represent the percentage of genes in each category. To ease visualization, percentages are not reported for fragmented genes that accounted for less than 2% of the total. The total amount of complete BUSCO genes for each species is given by the sum of "Complete and single copy" (light blue) and "Complete and duplicated" (dark blue).

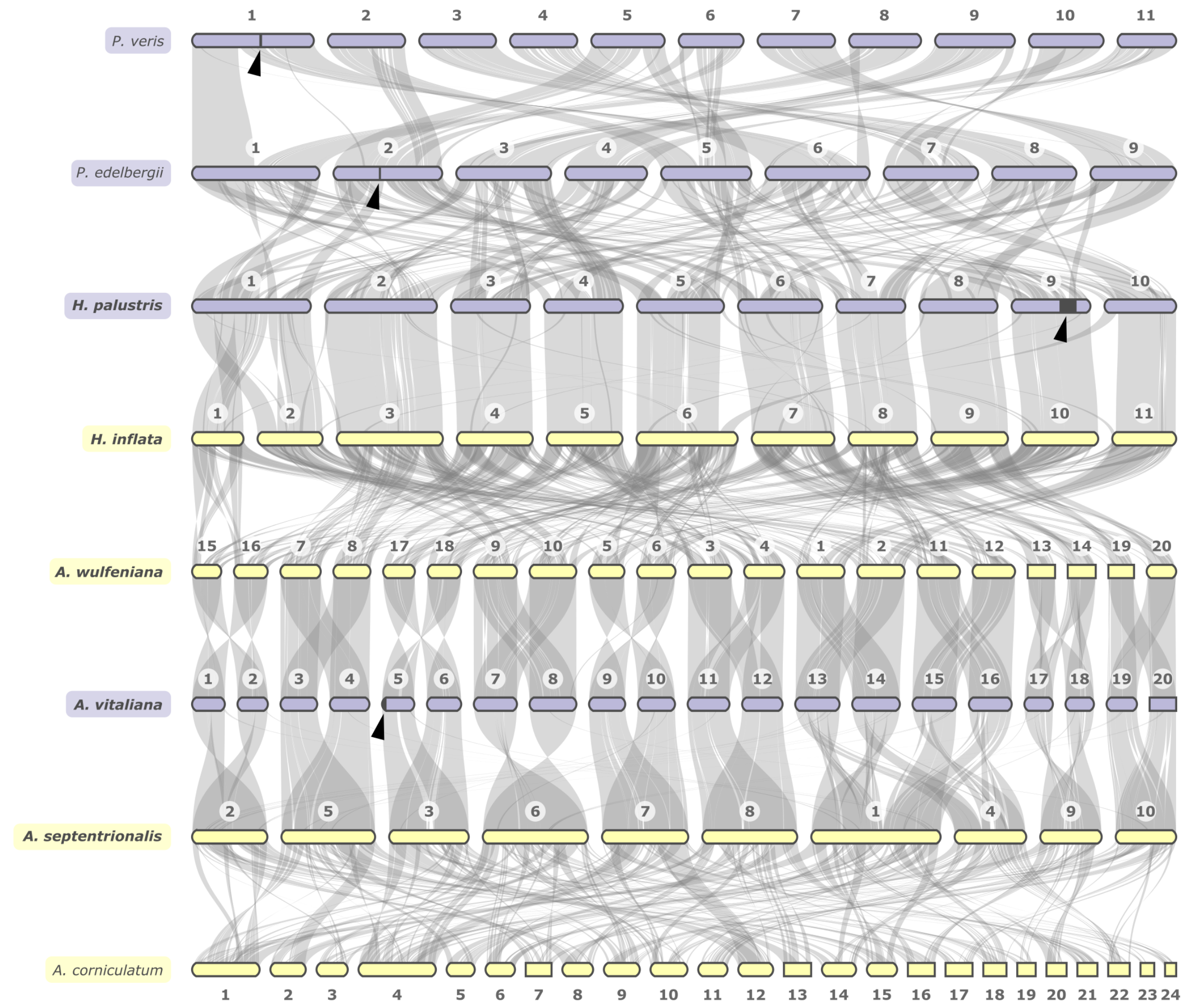

Fig. S11: Lack of synteny across Primulaceae.

Whole-genome synteny plots among Primulaceae. Distylous and non-distylous species are colored in purple and yellow, respectively. The *S*-loci are highlighted in the bars representing the chromosomes and marked by black triangles. Species whose genome assemblies were generated in this study are boldfaced.

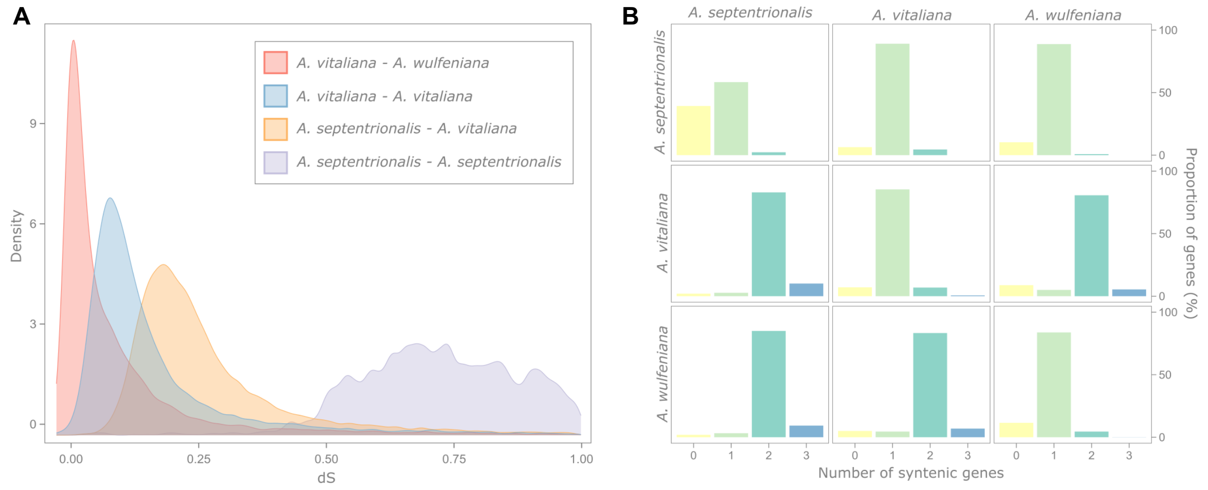

Fig. S12: Evidence for a WGD shared by *Androsace vitaliana* and *Androsace wulfeniana*. **A.** Distributions of synonymous divergence (d_S) calculated between syntenic orthologs of *A. vitaliana* - *A. wulfeniana* (n=13,078, calculated only for one subgenome; red curve), *A. septentrionalis* - *A. vitaliana* (n=16,414; orange curve), and between syntenic paralogs within *A. vitaliana* (n=28,899; blue curve) and *A. septentrionalis* (n=5,567; purple curve). The WGD observed in *A. vitaliana* (blue curve) is shared with *A. wulfeniana* (i.e. it occurred before the divergence between these two species, here represented by the red curve), but not with *A. septentrionalis*, as it is more recent than the divergence between *A. vitaliana* and *A. septentrionalis* (orange curve). *Androsace septentrionalis* only shows signatures of an ancient WGD, likely corresponding to the *Pv-α* WGD event previously identified in a study on *P. veris* (*27*). **B.** Bar plots indicating the number of syntenic genes present in each species comparison within *Androsace*. Each box indicates how many homologous syntenic genes in the genome of the species on the x-axis can be found in the genome of the species on the y-axis. Most genes of *A. septentrionalis* have two paralogs in both *A. vitaliana* and *A. wulfeniana*, indicating that a WGD occurred after the divergence between *A. septentrionalis* and the common ancestor of *A. vitaliana* and *A. wulfeniana*.

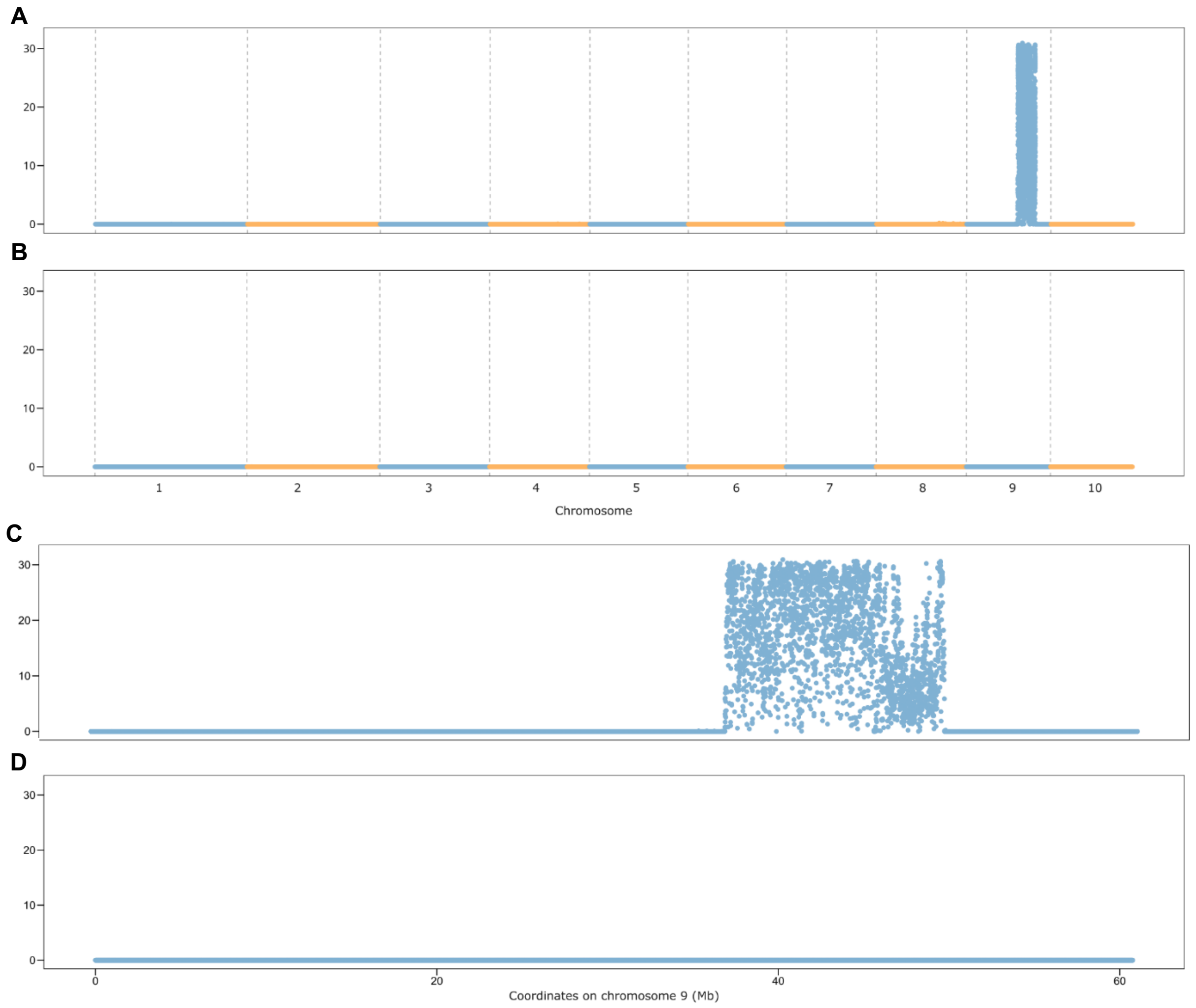

Fig. S13: Distribution of morph-specific *k*-mers in *Hottonia palustris.*
Morph-specific *k*-mers were identified from 28 samples (14 pins and 14 thrums) collected in Switzerland, France, and Germany. **A-B.** Percentage of sequence covered by thrum-specific (**A**) and pin-specific (**B**) *k*-mers (calculated in 5-kb windows) in the *H. plaustris* assembly (thrum). **C-D.** Zoom-in on chromosome 9, showing the distribution of thrum-specific (**C**) and pin-specific (**D**) *k*-mers. A region on chromosome 9 (36.76-49.53 Mb) is enriched in thrum-specific *k*-mers (**A** and **C**). Conversely, there is no region enriched in pin-specific *k*-mers (**B** and **D**).

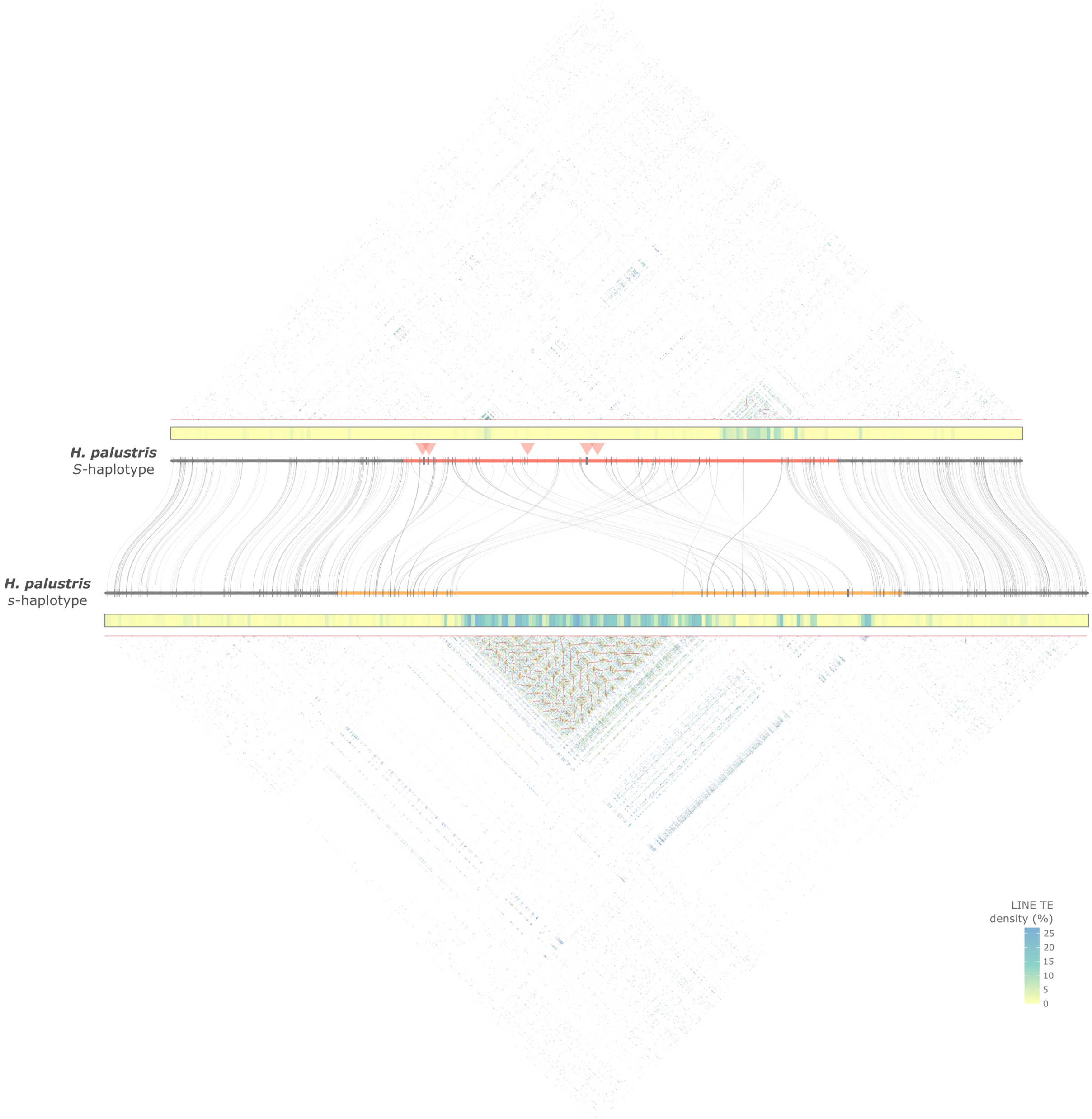

Fig. S14: Microsynteny and self-identity heatmap of the two *Hottonia palustris S*-locus haplotypes.

Microsynteny between the *S*- and *s*-haplotypes of *H. palustris*. The orthologs to *Primula S*-genes are indicated by red triangles. On top and bottom of the two haplotypes are density plots representing the proportion of sequence covered by LINE TEs (calculated in 100-kb windows), which are enriched in centromeric regions (see also **fig. S8** and **S9**). On top and bottom of the LINE density plots are self-identity heatmaps generated with ModDotPlot, showing higher-order tandem repeats corresponding to the putative LINE-rich centromeres.

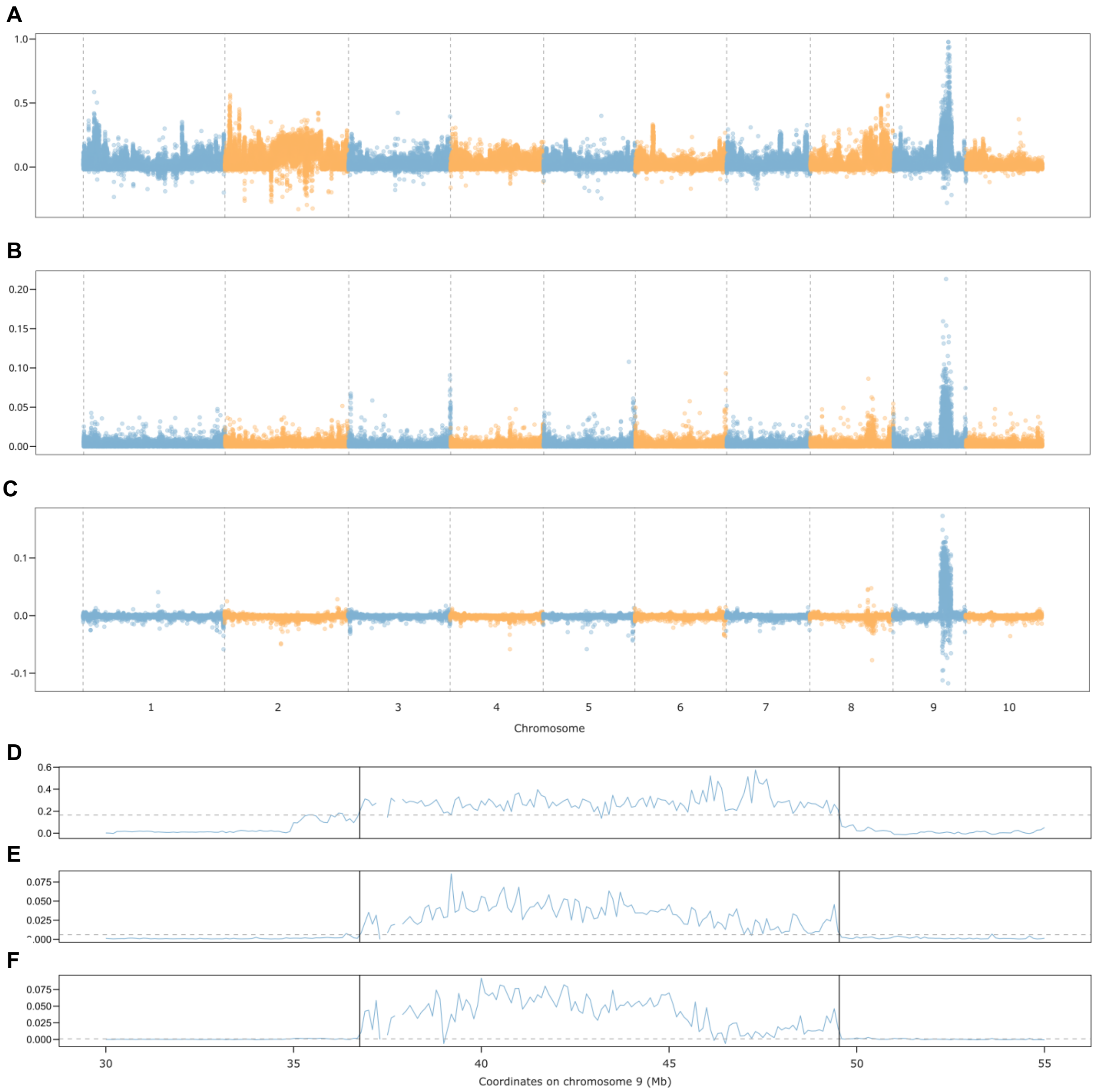

Fig. S15: Population genetic evidence for the localization of the *S*-locus in *Hottonia palustris*.

A-C. F_{ST} (**A**), D_{XY} (**B**), and morph-biased heterozygosity (**C**; defined as heterozygosity in thrums minus heterozygosity in pins), calculated in 5-kb windows across the *H. palustris* genome (thrum haplotype) using a population of 14 pins and 14 thrums. A peak is visible on chromosome 9 for each distribution. **D-F.** Zoom-in on the region of chromosome 9 (30-55 Mb), characterized by elevated F_{ST} (**D**), D_{XY} (**E**), and morph-biased heterozygosity (**F**); black vertical lines delimit the *S*-locus; a horizontal dashed line corresponds to the 95th percentile of each distribution, calculated on the whole genome.

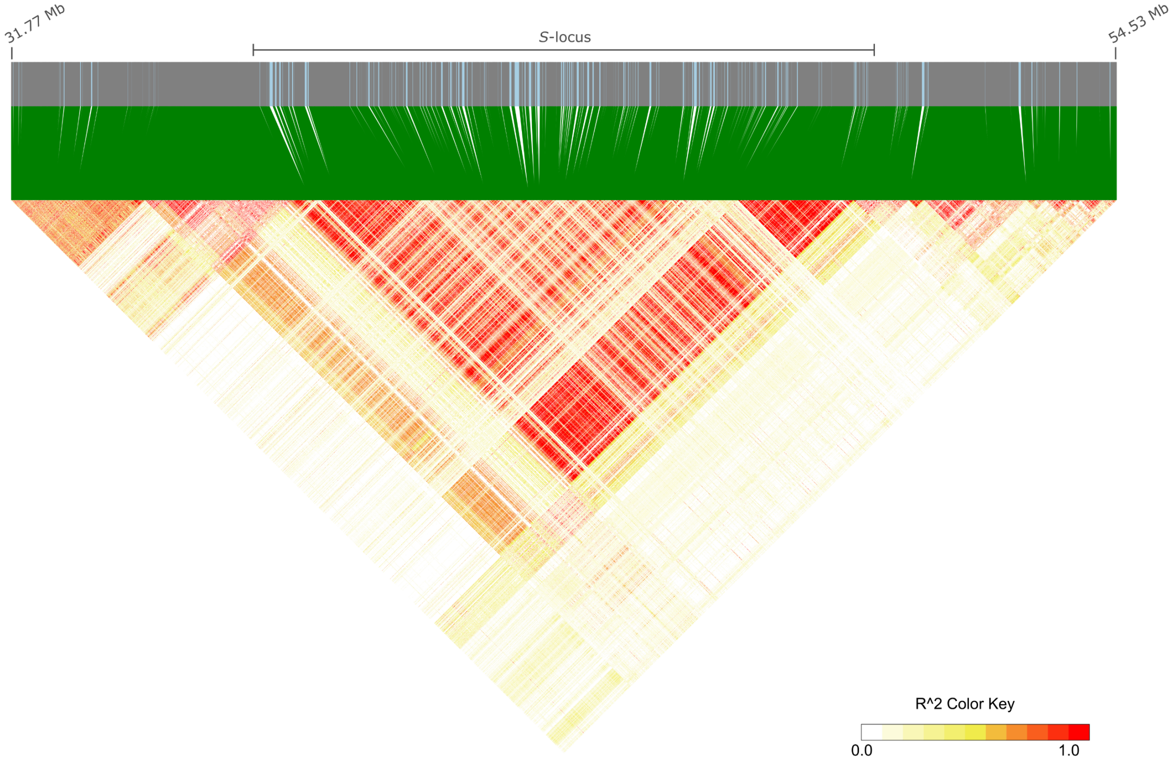

Fig. S16: Linkage disequilibrium heatmap for the *Hottonia palustris S*-locus.
Linkage disequilibrium (r^2) was estimated using data from 28 individuals on a 22.76-Mb region of *H. palustris* (thrum haplotype; chr9:31.768-54.531 Mb) spanning 4,294 SNPs across the *S*-locus and 5 Mb at each side of it. Heatmap cell colors represent r^2 values, with the corresponding scale shown in the legend.

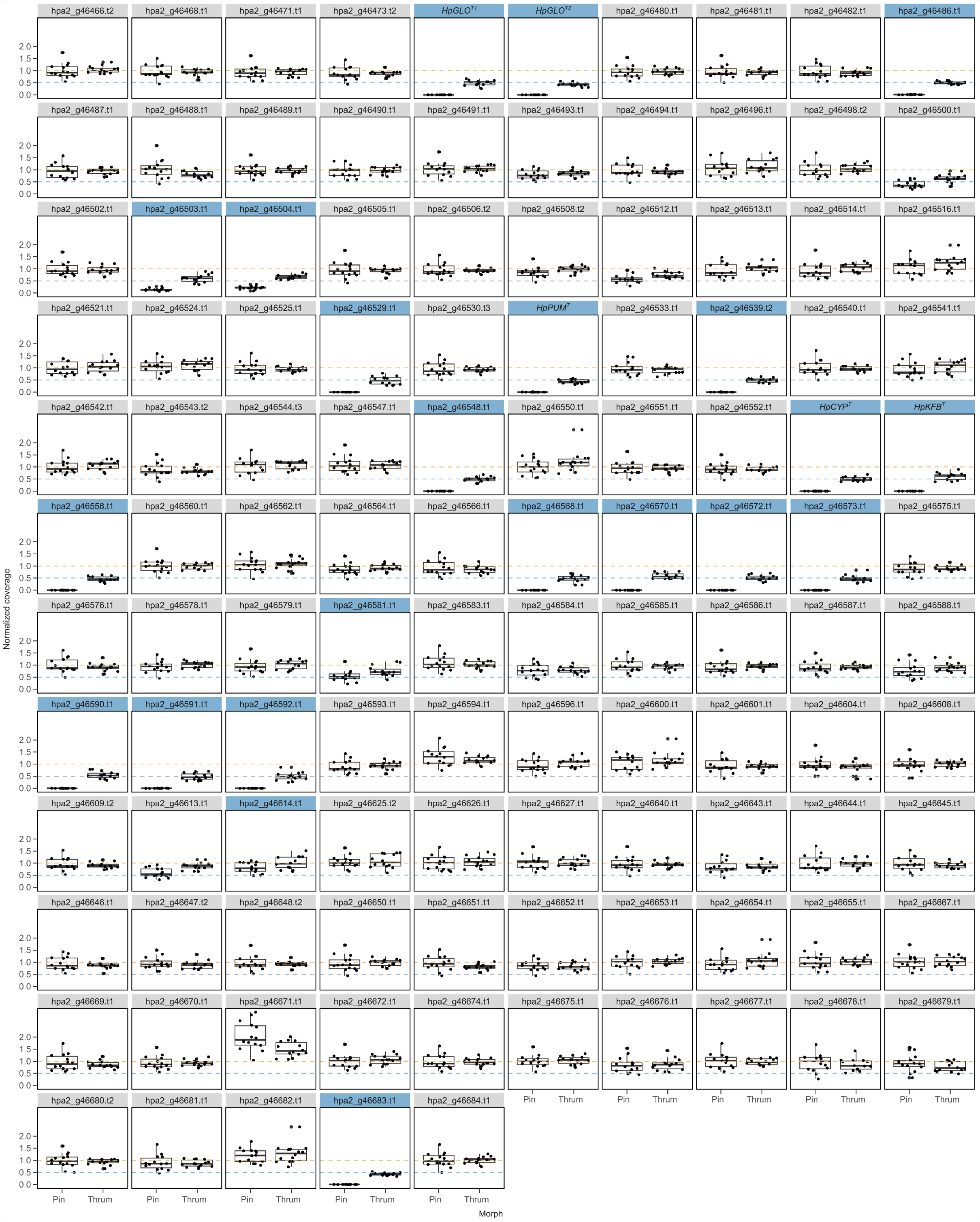

Fig. S17: Sequencing coverage of *Hottonia palustris S*-haplotype genes.

Box plots showing normalized sequencing coverage for 14 pins and 14 thrums across the 115 *S*-haplotype genes of *H. palustris*. The orange dashed line marks the mean normalized coverage across all genes on chromosome 9 (i.e., a value of 1). The blue dashed line indicates the expected normalized coverage for hemizygous genes (0.5). Genes specific to the *S*-haplotype are expected to have normalized coverage of 0.5 in thrums and zero coverage in pins (**table S16**). The 22 *S*-haplotype-specific genes are highlighted in blue, including the orthologs of *Primula S*-genes: $HpGLO^{T1}$ (hpa2_g46474.t1), $HpGLO^{T2}$ (hpa2_g46478.t1), $HpPUM^{T}$

(hpa2_g46531.t1), *HpCYP^T* (hpa2_g46555.t1), and *HpKFB^T* (hpa2_g46556.t1). Two genes (hpa2_g46581.t1 and hpa2_g46614.t1) show similar coverage in both morphs but are nevertheless *S*-haplotype-specific; each has a paralogous copy within the *S*-haplotype (hpa2_g46581.t1 is a duplicate of hpa2_g46575.t1; hpa2_g46614.t1 is a duplicate of hpa2_g46613.t1), resulting in inflated coverage due to cross-mapping.

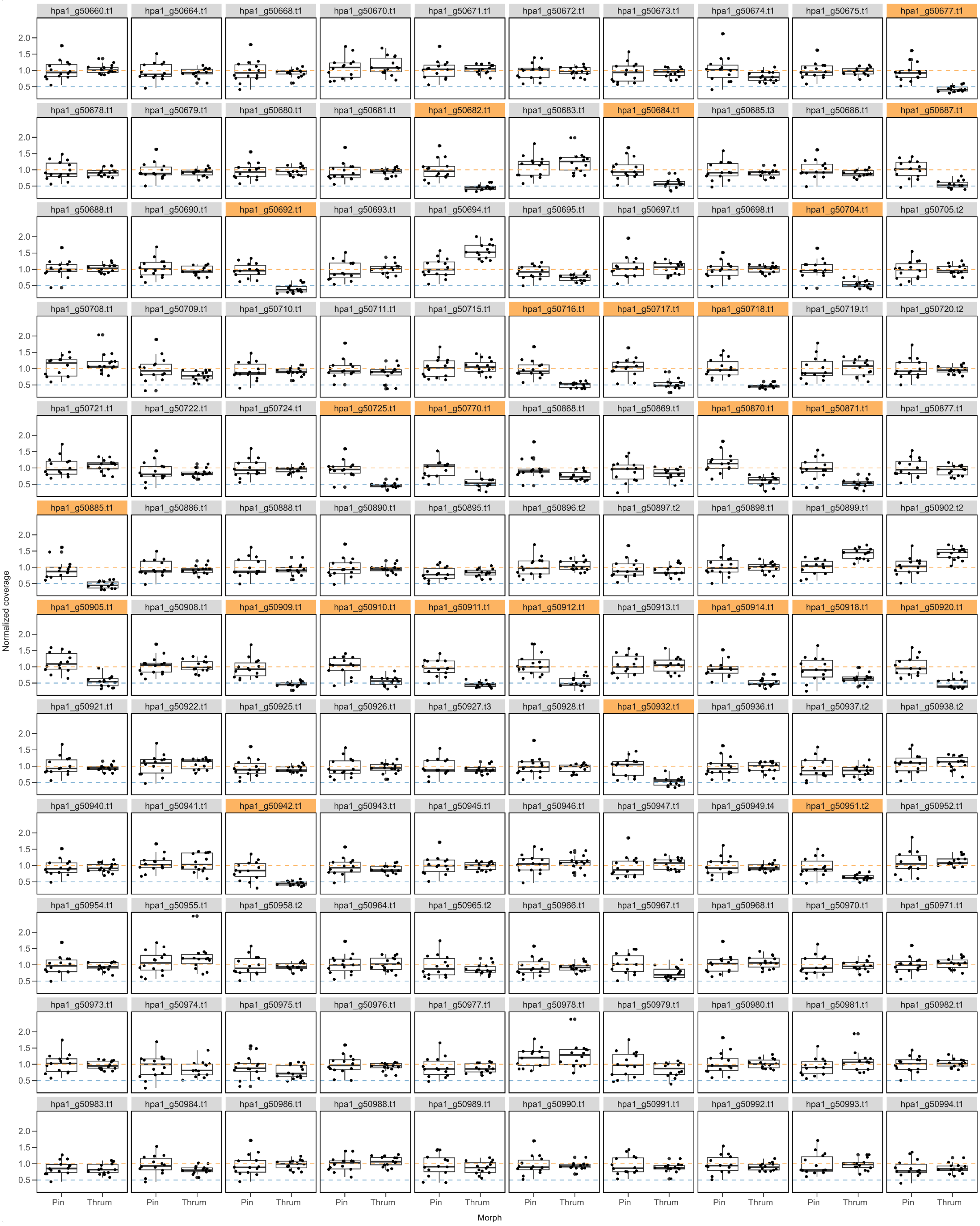

Fig. S18: Sequencing coverage of *Hottonia palustris s*-haplotype genes.
Box plots showing normalized sequencing coverage for 14 pins and 14 thrums across the 120 *s*-haplotype genes of *H. palustris*. The orange dashed line marks the mean normalized coverage across all genes on chromosome 9 (i.e., a value of 1). The blue dashed line indicates the expected normalized coverage for hemizygous genes (0.5). Genes specific to the *s*-haplotype are expected to have normalized coverage of 1 in pins and of 0.5 in thrums (**table S15**). The 25 *s*-haplotype-specific genes are highlighted in orange.

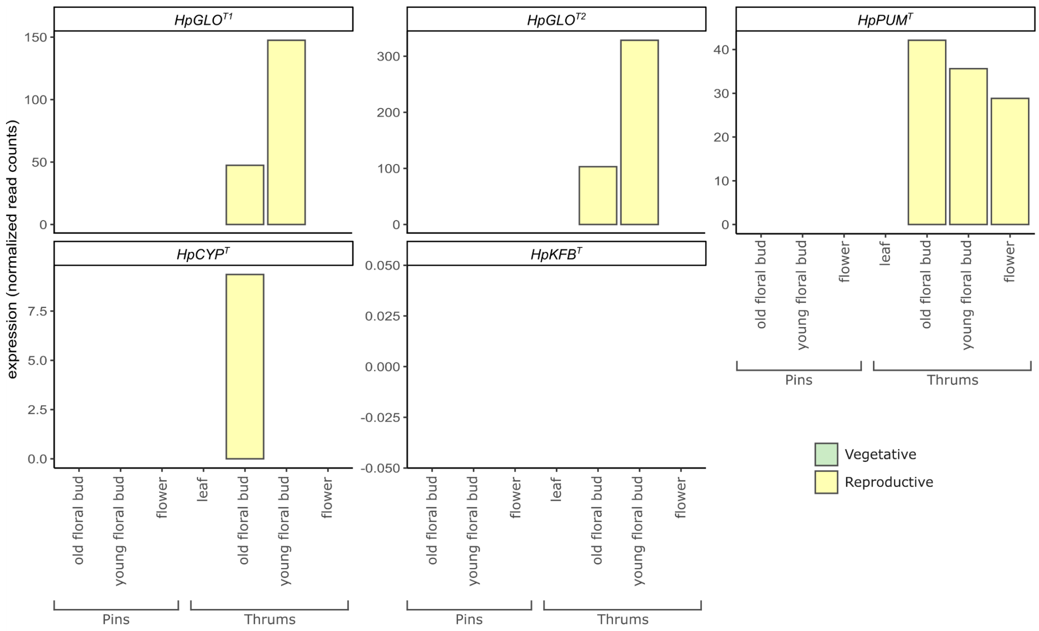

Fig. S19: Expression of the five *Hottonia palustris* orthologs of the *Primula S*-genes. Expression (as normalized read counts) of the five *H. palustris* orthologs of the *Primula S*-genes: apart from *HpKFB*^{T}, which is not expressed in any tissue, the other four *S*-genes are expressed only in floral samples of thrum individuals.

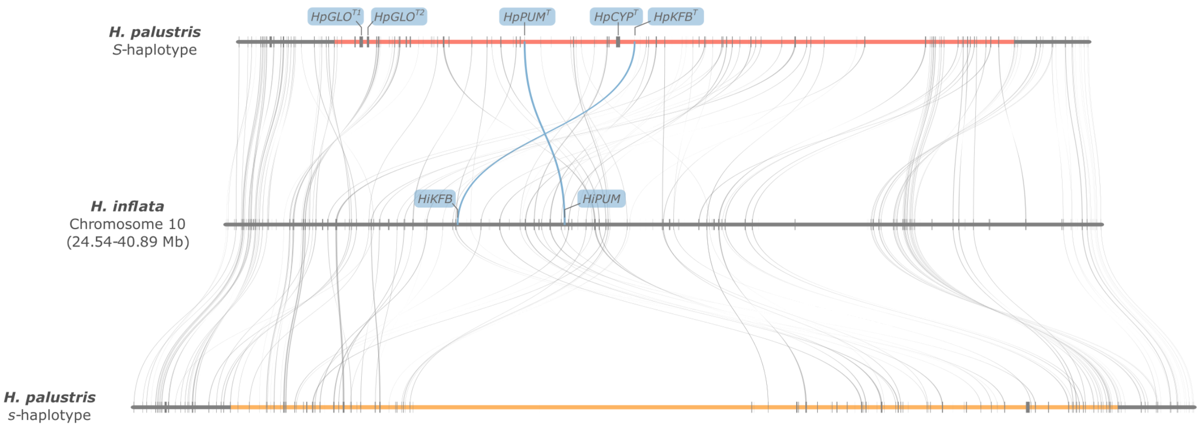

Fig. S20: The *Hottonia palustris S*-locus is rearranged compared to *Hottonia inflata*. Microsynteny plot between the *H. palustris S*- and *s*-haplotypes (top and bottom, respectively), and their syntenic region in *H. inflata* (center). The *H. palustris S*- and s-haplotypes are highlighted in red and orange, respectively; gene names are reported for orthologs of *Primula S*-genes. *Hottonia inflata* contains an *S*-locus lacking orthologs of GLO^{T} and CYP^{T}, indicating that this species represents a secondary loss of distyly. Furthermore, this plot shows that several rearrangements occurred in this genomic region since the divergence between *H. palustris* and *H. inflata*.

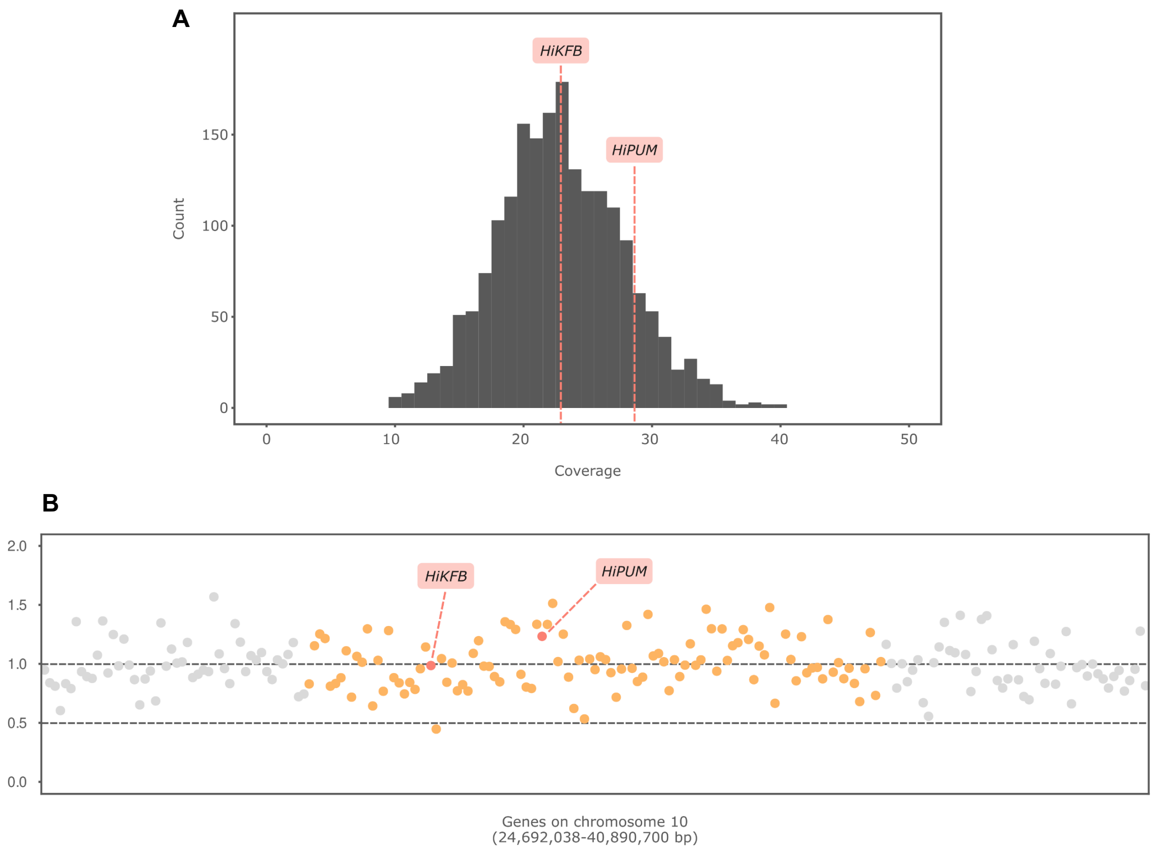

Fig. S21: The orthologs of *Primula S*-genes KFB^T and PUM^T are present in both haplotypes of *Hottonia inflata.*

A. Distribution of sequencing coverage across all genes on chromosome 10 of *H. inflata*, which contains the region syntenic to the *H. palustris S*-locus. The sequencing coverage of *HiKFB* (22.90x) and *HiPUM* (28.64x) is indicated by red vertical lines. Neither gene differs significantly from the mean coverage across all genes on chromosome 10 (23.14x; n=1,929; z-test, $P > 0.05$), indicating that the two genes are present in both haplotypes in the sequenced individual. **B.** Normalized sequencing coverage for all genes in the *H. inflata* genomic region syntenic to the *H. palustris S*-locus (orange), with *HiKFB* and *HiPUM* highlighted in red. For comparison, 50 flanking genes at each side of the region of interest are shown in grey.

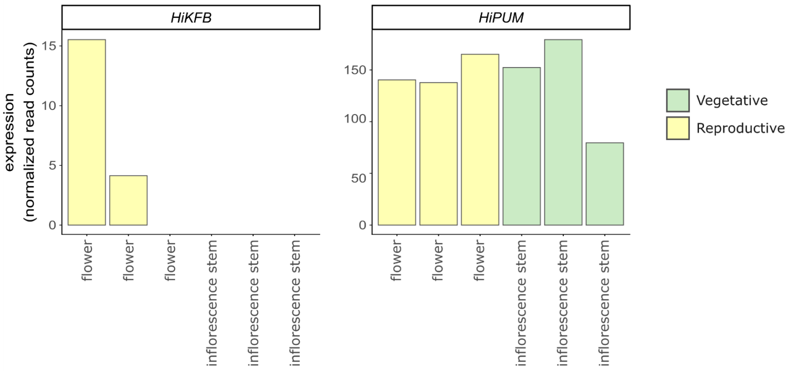

Fig. S22: Expression of the two *Hottonia inflata* orthologs of the *Primula S*-genes.
Expression (as normalized read counts) of the two *H. inflata* orthologs of the *Primula S*-genes: *HiKFB* is expressed at extremely low levels in the flower, while *HiPUM* is expressed both in reproductive (i.e. flower) and vegetative (i.e. inflorescence stem) tissues, reflecting previous observations in *Primula*.

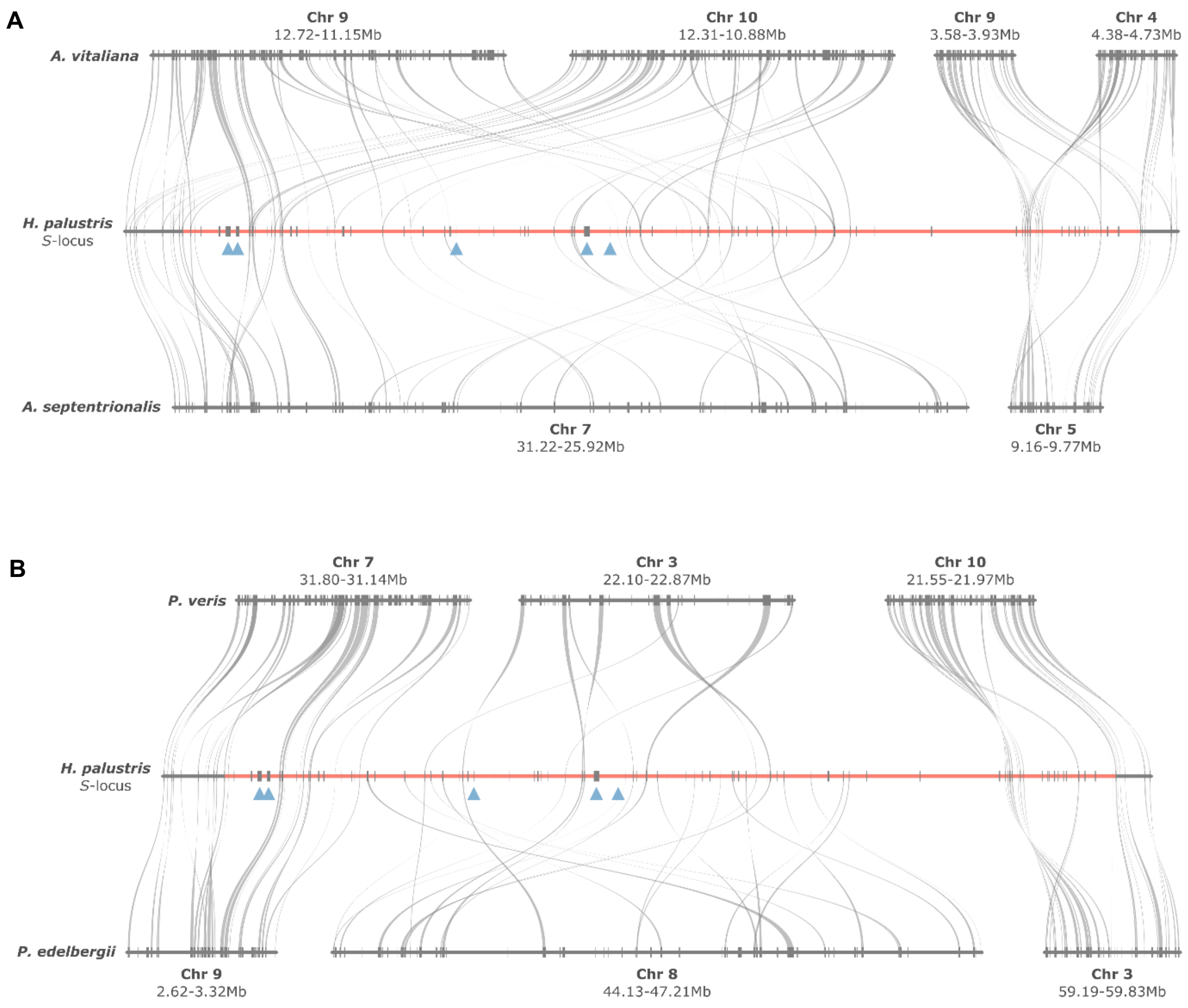

Fig. S23: The *Hottonia palustris S*-locus lies in a region not syntenic with *Androsace* nor *Primula*.

A. Microsynteny plot between the *H. palustris S*-locus (*S*-haplotype; center), *A. vitaliana* (thrum haplotype; top) and *A. septentrionalis* (bottom). **B.** Microsynteny plot between the *H. palustris S*-locus (*S*-haplotype; center), *P. veris* (top) and *P. edelbergii* (bottom). The region containing the *S*-locus in *H. palustris* (chromosome 9: 36.00-50.07 Mb) has the *S*-locus colored red and the five hemizygous orthologs of the *Primula S*-genes marked by blue triangles (left to right: $HpGLO^{T1}$, $HpGLO^{T2}$, $HpPUM^{T}$, $HpCYP^{T}$, and $HpKFB^{T}$); none of these five genes is contained in a region syntenic with any other Primulaceae. Instead, parts of the *H. palustris S*-locus are syntenic with multiple regions in different chromosomes in each comparison, indicating that the *S*-locus: i) translocated after the divergence between *Hottonia* and *P. veris*/*P. edelbergii* and ii) underwent several structural rearrangements in *H. palustris*, incorporating additional genes.

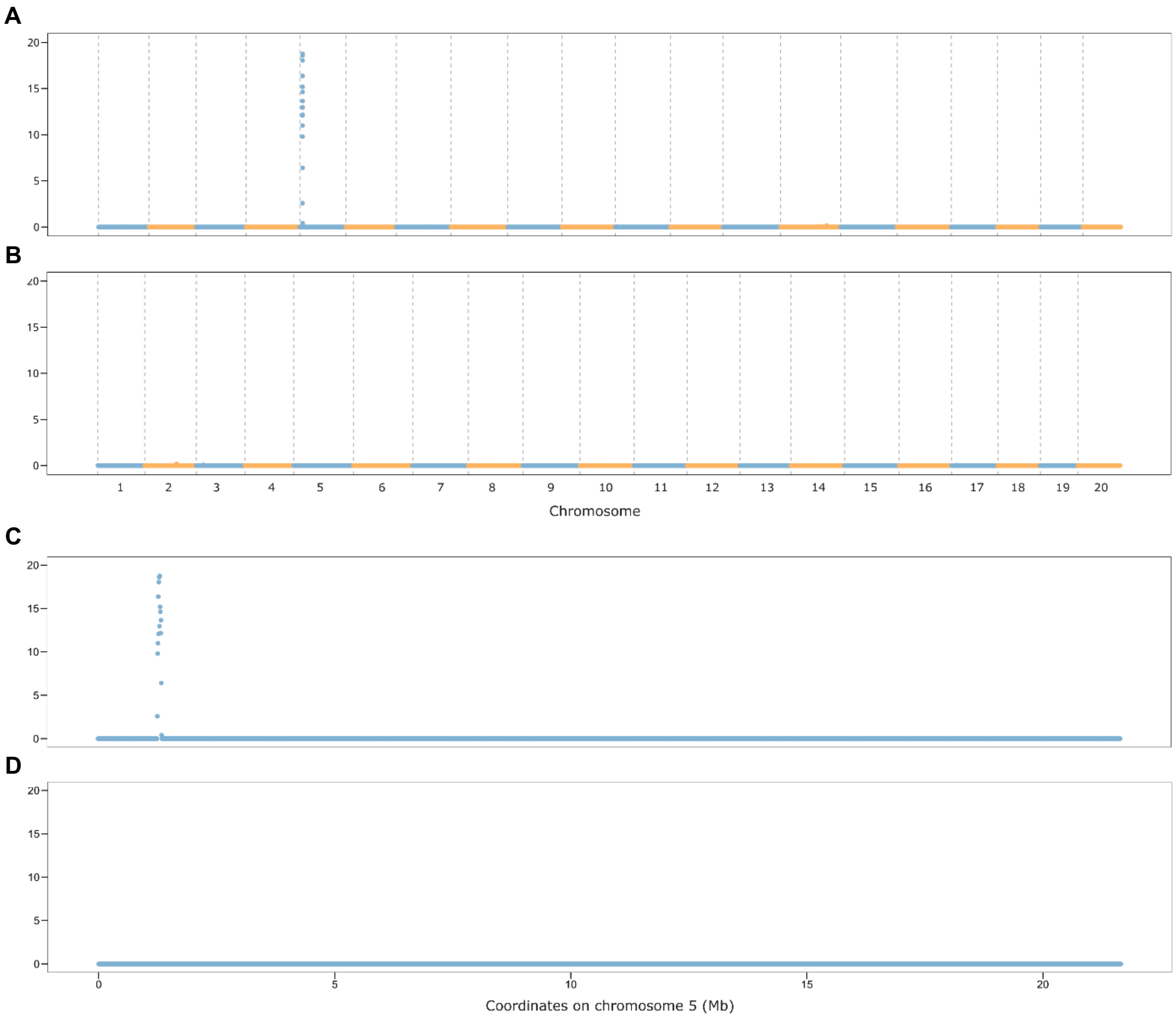

Fig. S24: Distribution of morph-specific *k*-mers in *Androsace vitaliana* (Wallis samples). Morph-specific *k*-mers were identified from 24 samples (12 pins and 12 thrums) of a population collected in the Wallis canton (Switzerland). **A-B.** Percentage of sequence covered by thrum-specific (**A**) and pin-specific (**B**) *k*-mers (calculated in 5-kb windows) in the *A. vitaliana* assembly (thrum). **C-D.** Zoom-in on chromosome 5, showing the distribution of thrum-specific (**C**) and pin-specific (**D**) *k*-mers. A region on chromosome 5 (950-1,100 kb) is enriched in thrum-specific *k*-mers (**A** and **C**). Conversely, there is no region enriched in pin-specific *k*-mers (**B** and **D**).

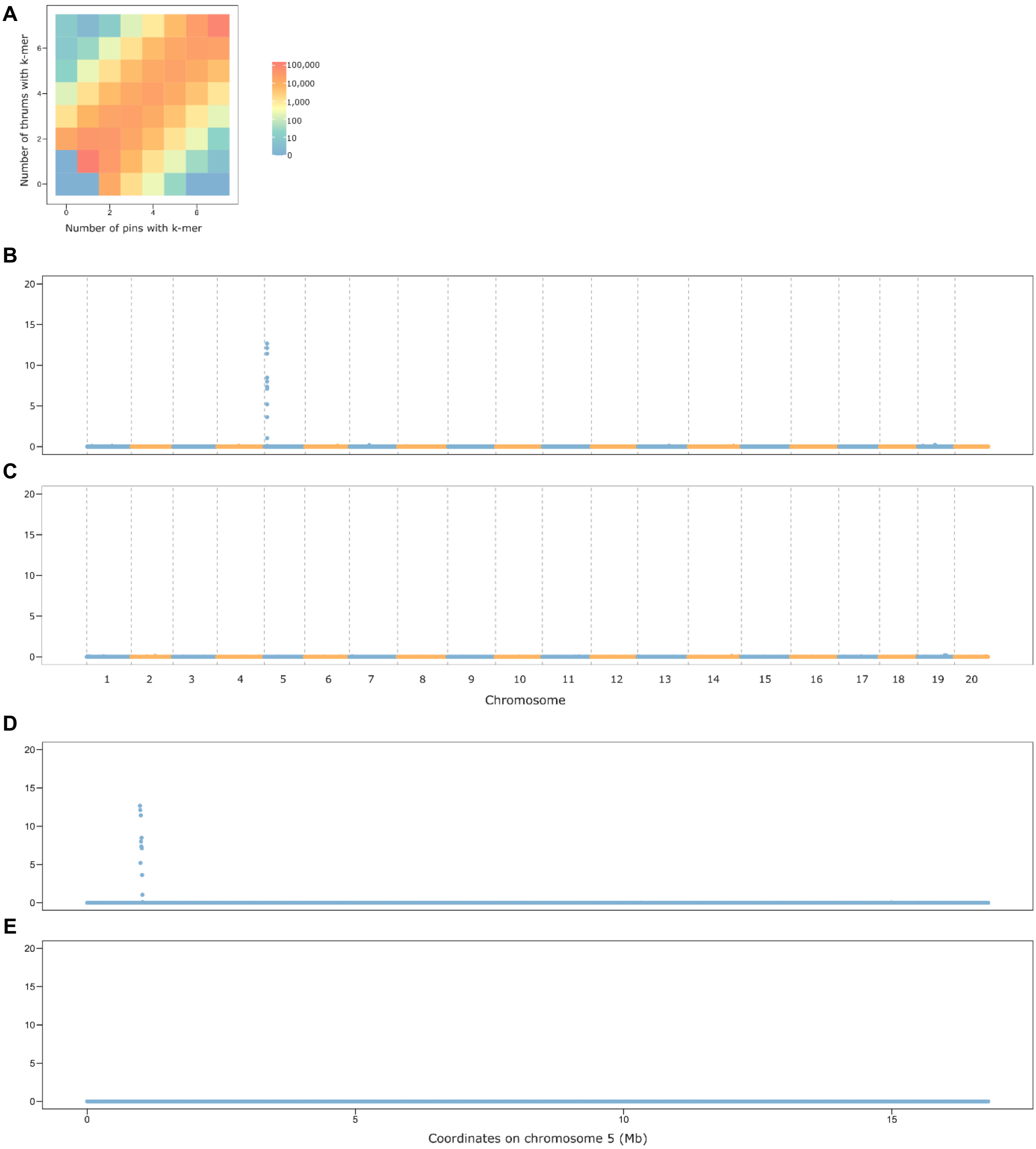

Fig. S25: Distribution of morph-specific *k*-mers in *Androsace vitaliana* (herbarium samples).

Morph-specific *k*-mers were identified from 14 samples (7 pins and 7 thrums) whose DNA was extracted from herbarium specimens. a-b. Percentage of sequence covered by thrum-specific (**A**) and pin-specific (**B**) *k*-mers (calculated in 5-kb windows) in the *A. vitaliana* assembly (thrum). **C-D.** Zoom-in on chromosome 5, showing the distribution of thrum-specific (**C**) and pin-specific (**D**) *k*-mers. A region on chromosome 5 (950-1,100 kb) is enriched in thrum-specific *k*-mers (**A** and **C**). Conversely, there is no region enriched in pin-specific *k*-mers (**B** and **D**).

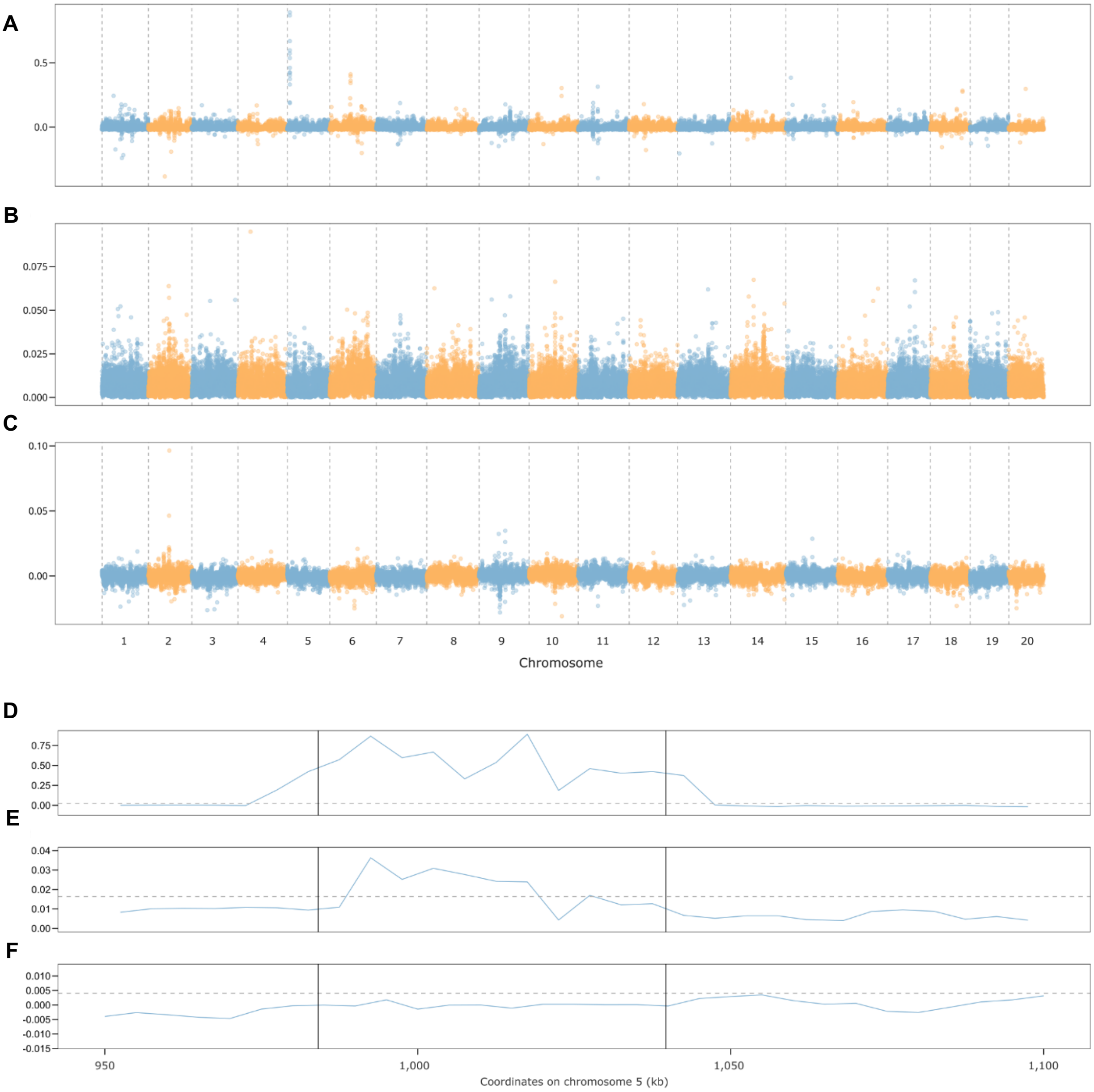

Fig. S26: Population genetic evidence for the localization of the *S*-locus in *Androsace vitaliana* (Wallis samples).

A-C. F_{ST} (**A**), D_{XY} (**B**), and morph-biased heterozygosity (**C**; defined as heterozygosity in thrums minus heterozygosity in pins), calculated in 5-kb windows across the *A. vitaliana* genome (thrum haplotype) using 24 samples (12 pins and 12 thrums) of a population collected in the Wallis canton (Switzerland). A peak is visible on chromosome 5 for the F_{ST} distribution. **D-F.** Zoom-in on the region of chromosome 5 (950-1,100 kb), characterized by elevated F_{ST} (**D**). Black vertical lines delimit the *S*-locus; a horizontal dashed line corresponds to the 95th percentile of each distribution, calculated on the whole genome. Values above the 95th percentile were detected in this region for F_{ST} (**D**) and D_{XY} (**E**), but not for the morph-biased heterozygosity (**F**). This is likely due to the small size of the *S*-locus in *A. vitaliana* (ca. 55 kb) and to its high TE content. Indeed, higher heterozygosity in thrums was observed when looking at single SNPs (Fig. 3 in the main text).

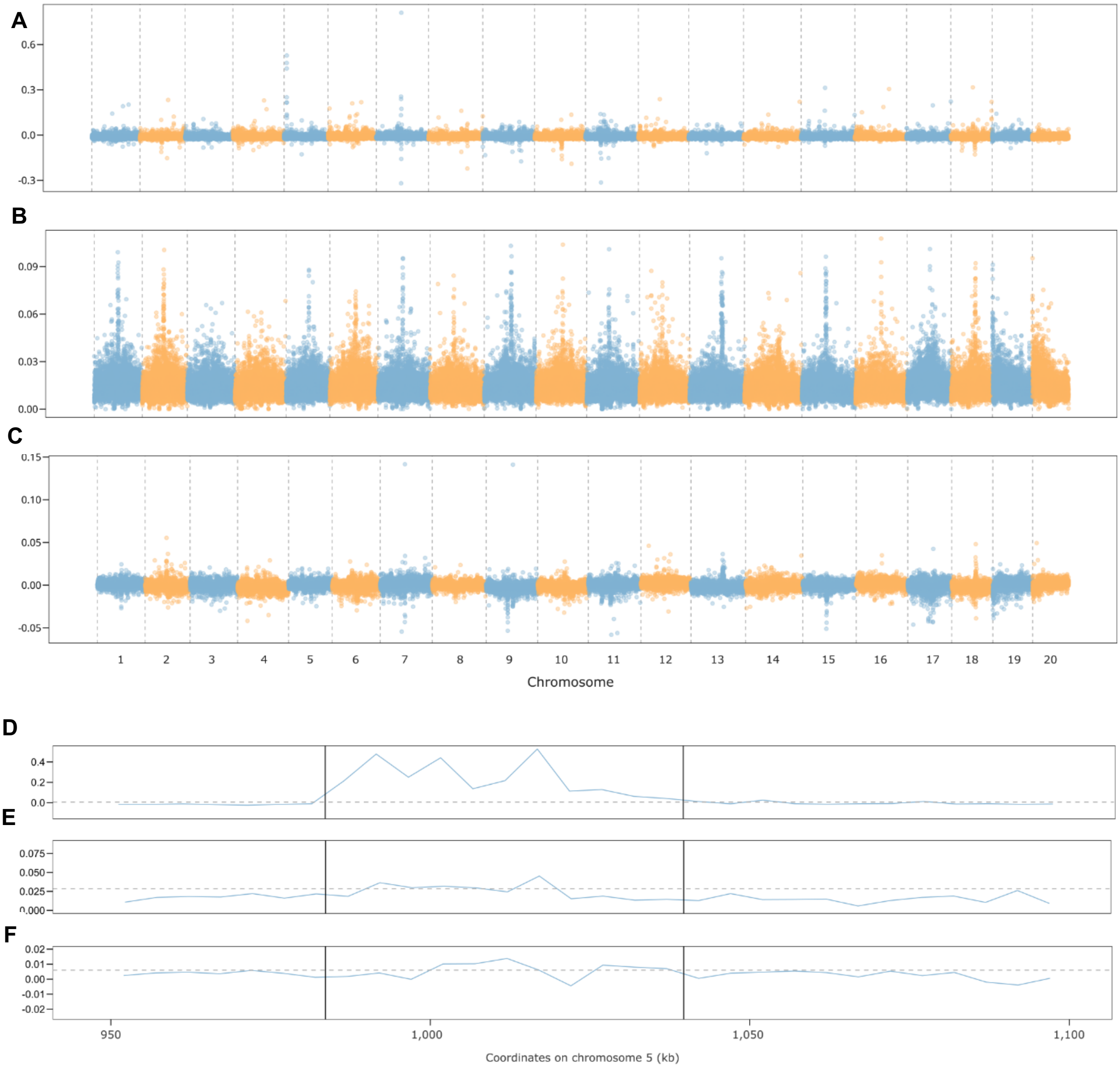

Fig. S27: Population genetic evidence for the localization of the *S*-locus in *Androsace vitaliana* (herbarium samples).

A-C. F_{ST} (**A**), D_{XY} (**B**), and morph-biased heterozygosity (**C**; defined as heterozygosity in thrums minus heterozygosity in pins), calculated in 5-kb windows across the *A. vitaliana* genome (thrum haplotype) using 14 samples (7 pins and 7 thrums) whose DNA was extracted from herbarium specimens. A peak is visible on chromosome 5 for the F_{ST} distribution. **D-F**. Zoom-in on the region of chromosome 5 (950-1,100 kb), characterized by elevated F_{ST} (**D**). Black vertical lines delimit the *S*-locus; a horizontal dashed line corresponds to the 95th percentile of each distribution, calculated on the whole genome. Values above the 95th percentile were detected in this region for F_{ST} (**D**) and D_{XY} (**E**), but not for the morph-biased heterozygosity (**F**). This is likely due to the small size of the *S*-locus in *A. vitaliana* (ca. 55 kb) and to its high TE content. Indeed, higher heterozygosity in thrums was observed when looking at single SNPs (Fig. 3 in the main text).

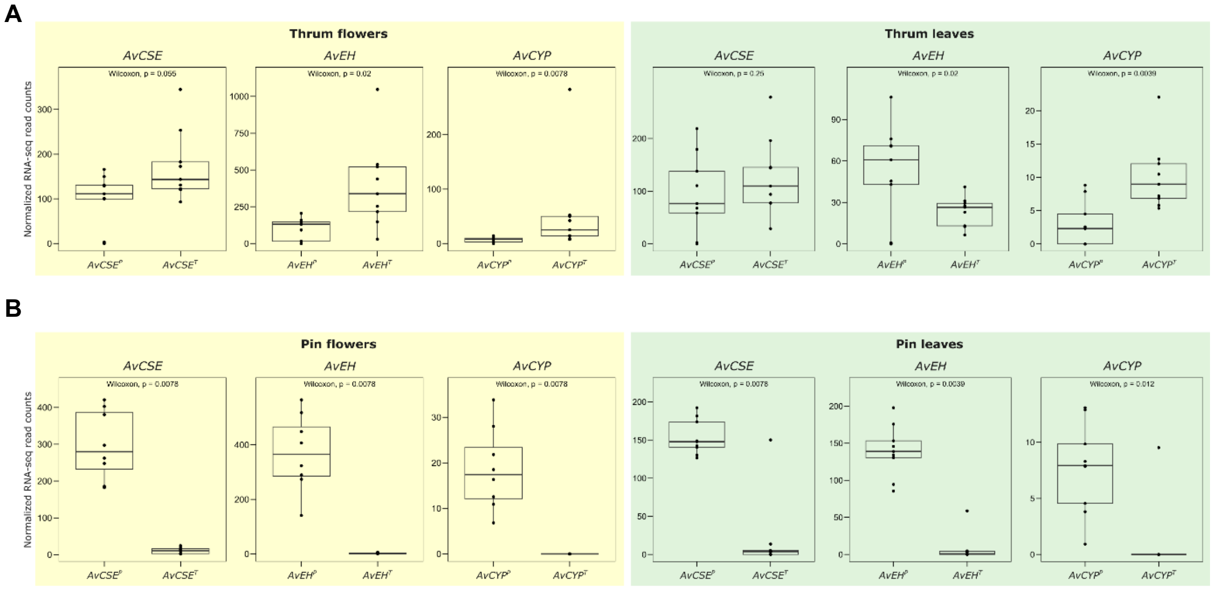

Fig. S28: *Androsace vitaliana S*-alleles are upregulated in flowers compared to *s*-alleles. Box plots comparing the expression of *s*- (left) and *S*- (right) alleles in flowers (yellow box) and leaves (green box). **A.** Results from nine thrum flower samples and nine thrum leaf samples. In flowers, $AvEH^T$ and $AvCYP^T$ were upregulated compared to $AvEH^P$ and $AvCYP^P$, while *AvCSE* alleles were expressed at the same level; in leaves, $AvEH^P$ was upregulated compared to $AvEH^T$, *AvCSE* alleles were expressed at the same level, and both *AvCYP* alleles were expressed at extremely low levels. **B.** Results from eight pin flower samples and nine pin samples. In pins, no expression was detected for *S*-alleles, as expected, since pins only carry the *s*-haplotype; this result proves that the difference in expression detected in thrums is not due to a bias in RNA-seq read mapping but rather represents a true biological difference.

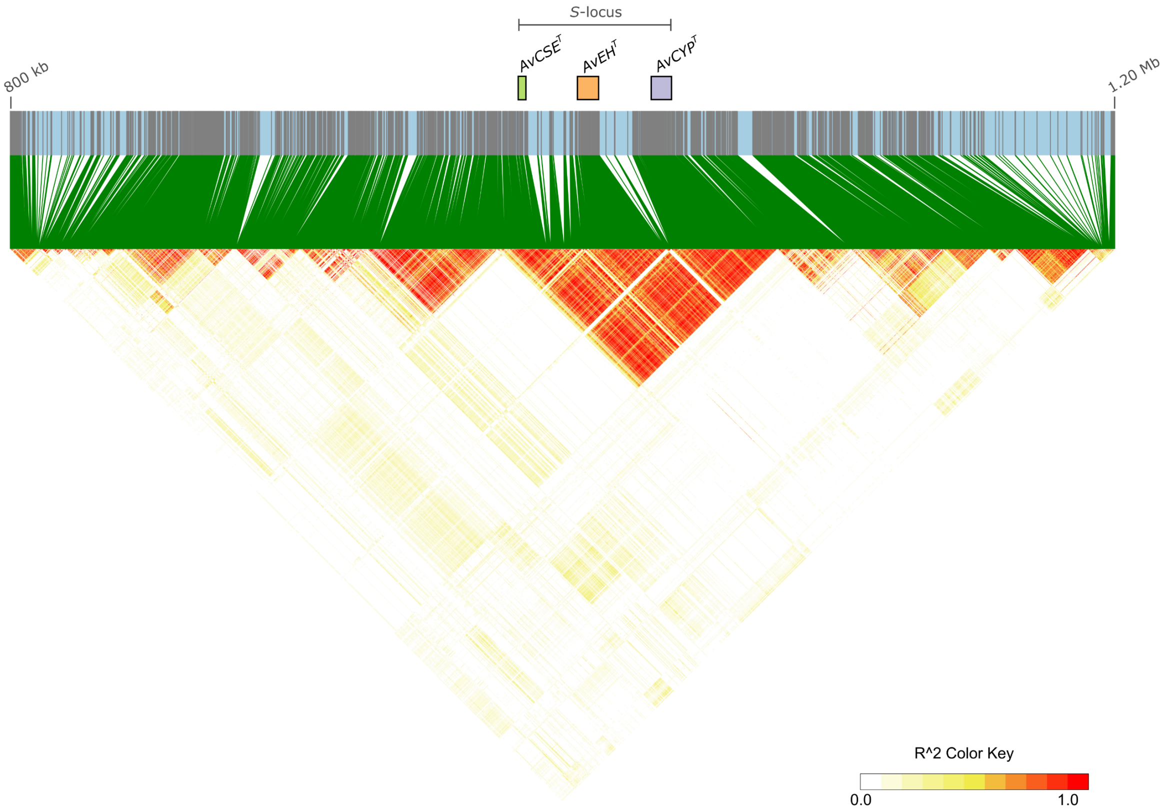

Fig. S29: Linkage disequilibrium heatmap for the *Androsace vitaliana S*-locus (Wallis samples).

Linkage disequilibrium (r^2) was estimated using data from 24 individuals (Wallis population) on a 400-kb region of *A. vitaliana* (thrum haplotype; chr5: chr5:800,000-1,200,000 bp) spanning 2,895 SNPs across the *S*-locus, 184 kb on its left side and 160 kb on its right side. Heatmap cell colors represent r^2 values, with the corresponding scale shown in the legend.

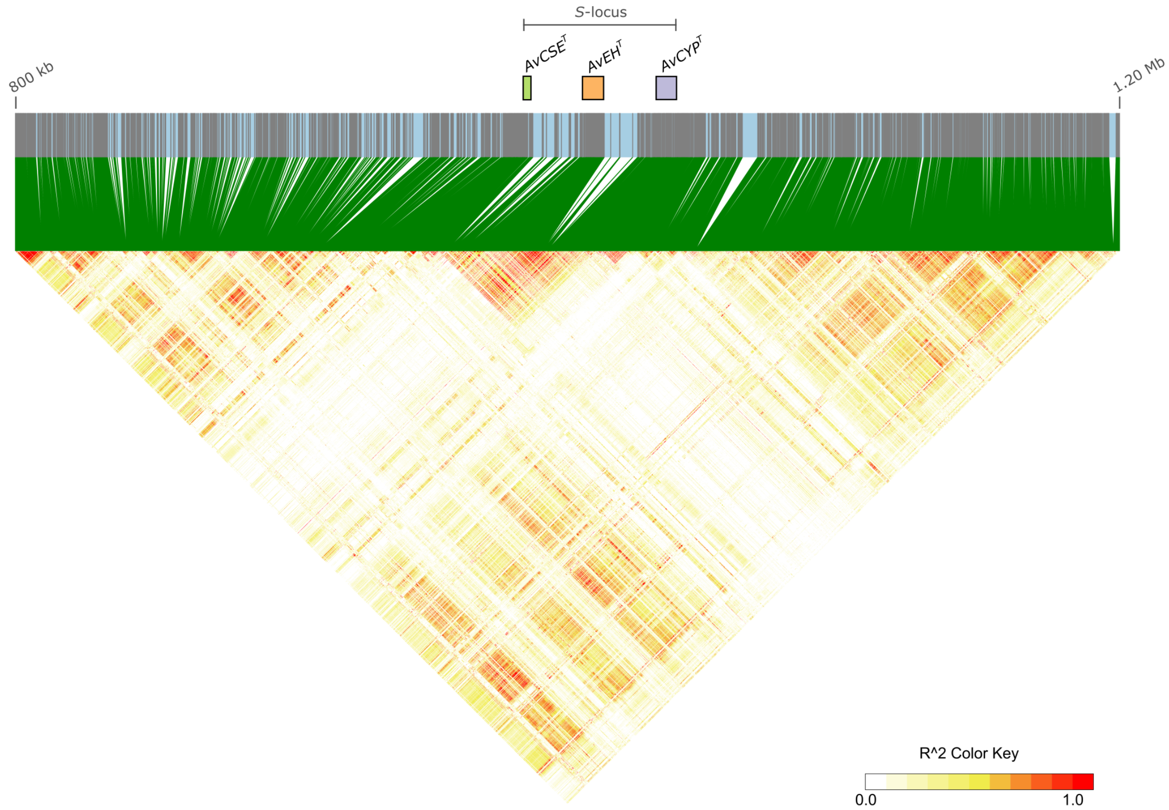

Fig. S30: Linkage disequilibrium heatmap for the *Androsace vitaliana S*-locus (herbarium samples).
Linkage disequilibrium (r^2) was estimated using data from 14 individuals (herbarium samples) on a 400-kb region of *A. vitaliana* (thrum haplotype; chr5:800,000-1,200,000 bp) spanning 3,470 SNPs across the *S*-locus, 184 kb on its left side and 160 kb on its right side. Heatmap cell colors represent r^2 values, with the corresponding scale shown in the legend.

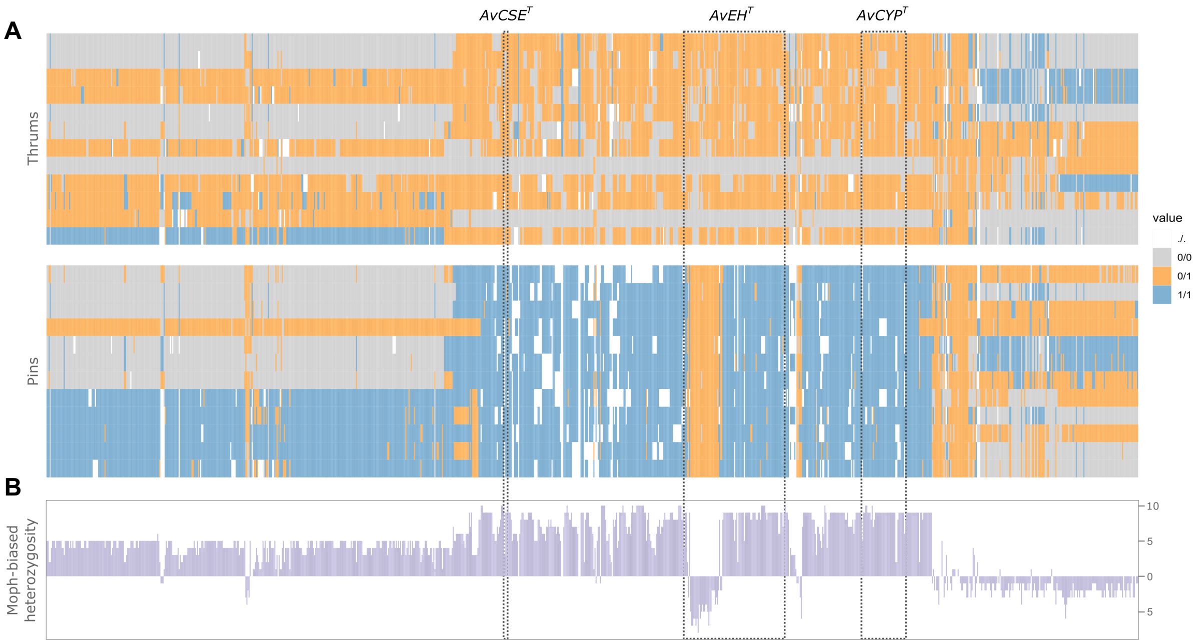

Fig. S31: Genotype variation across the *S*-locus region in *Androsace vitaliana* (Wallis samples).

A. Genotype tile plot for the 24 Wallis samples across 1,017 SNPs in chr5:950,000-1,100,000 bp(x-axis). Colors indicate genotype state (grey: reference homozygote; orange: heterozygote; blue: alternate homozygote; white: missing). SNPs falling within the three *S*-genes are enclosed by dashed boxes. **B.** Morph-biased heterozygosity, calculated for each SNP as the number of thrums that are heterozygous minus the number of pins that are heterozygous.

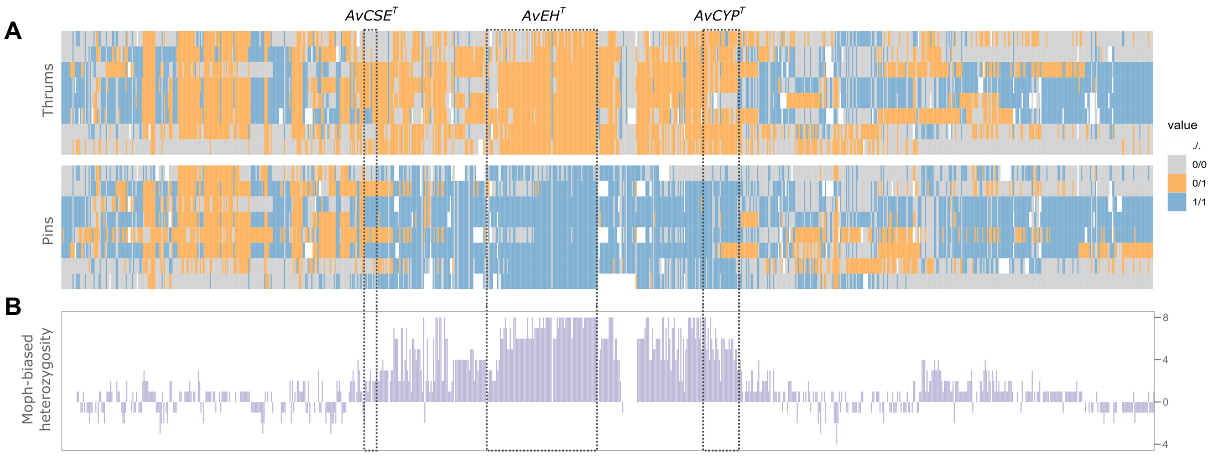

Fig. S32: Genotype variation across the *S*-locus region in *Androsace vitaliana* (herbarium samples).

A. Genotype tile plot for 16 individuals (14 herbarium samples plus the two individuals used for the genome assemblies; y-axis) across 882 SNPs in chr5:950,000-1,100,000 bp (x-axis). Colors indicate genotype state (grey: reference homozygote; orange: heterozygote; blue: alternate homozygote; white: missing). SNPs falling within the three *S*-genes are enclosed by dashed boxes. **B.** Morph-biased heterozygosity, calculated for each SNP as the number of thrums that are heterozygous minus the number of pins that are heterozygous.

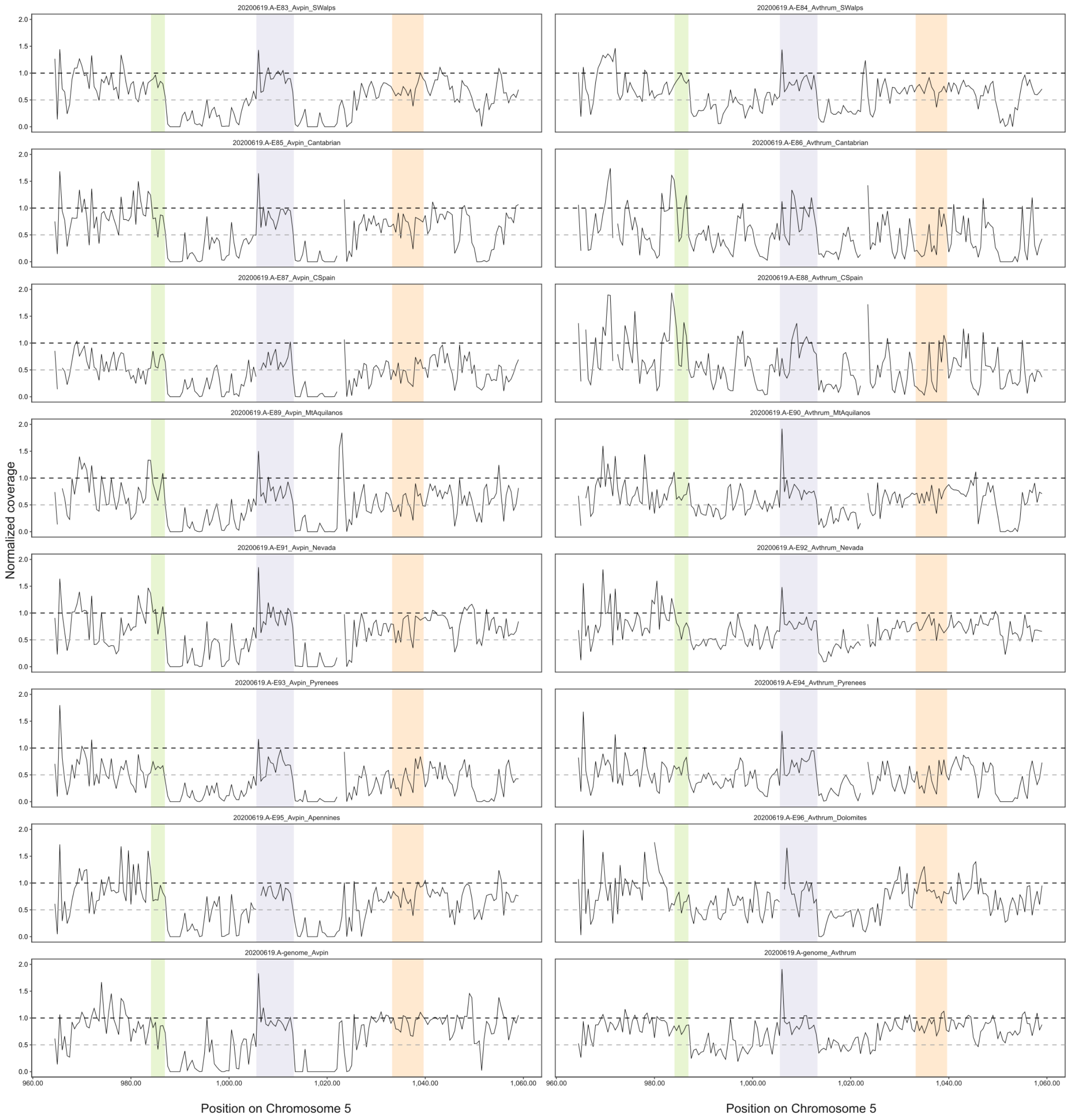

Fig. S33: Sequencing coverage of *Androsace vitaliana* pins and thrums across the *S*-locus (herbarium samples).

Normalized Illumina short-read coverage across the *S*-locus region (chr5:960,000-1,060,000 bp) for seven pin and seven thrum herbarium samples, plus the two individuals used for the genome assembly. Dashed horizontal lines indicate normalized coverage values of 1 (black) and 0.5 (grey). Colored boxes mark the positions of the three *S*-genes: $AvCSE^T$ (green), $AvEH^T$ (purple), and $AvCYP^T$ (orange). In all pin samples there are regions of zero coverage within the *S*-locus, while thrums are characterized by non-zero coverage across the whole *S*-locus.

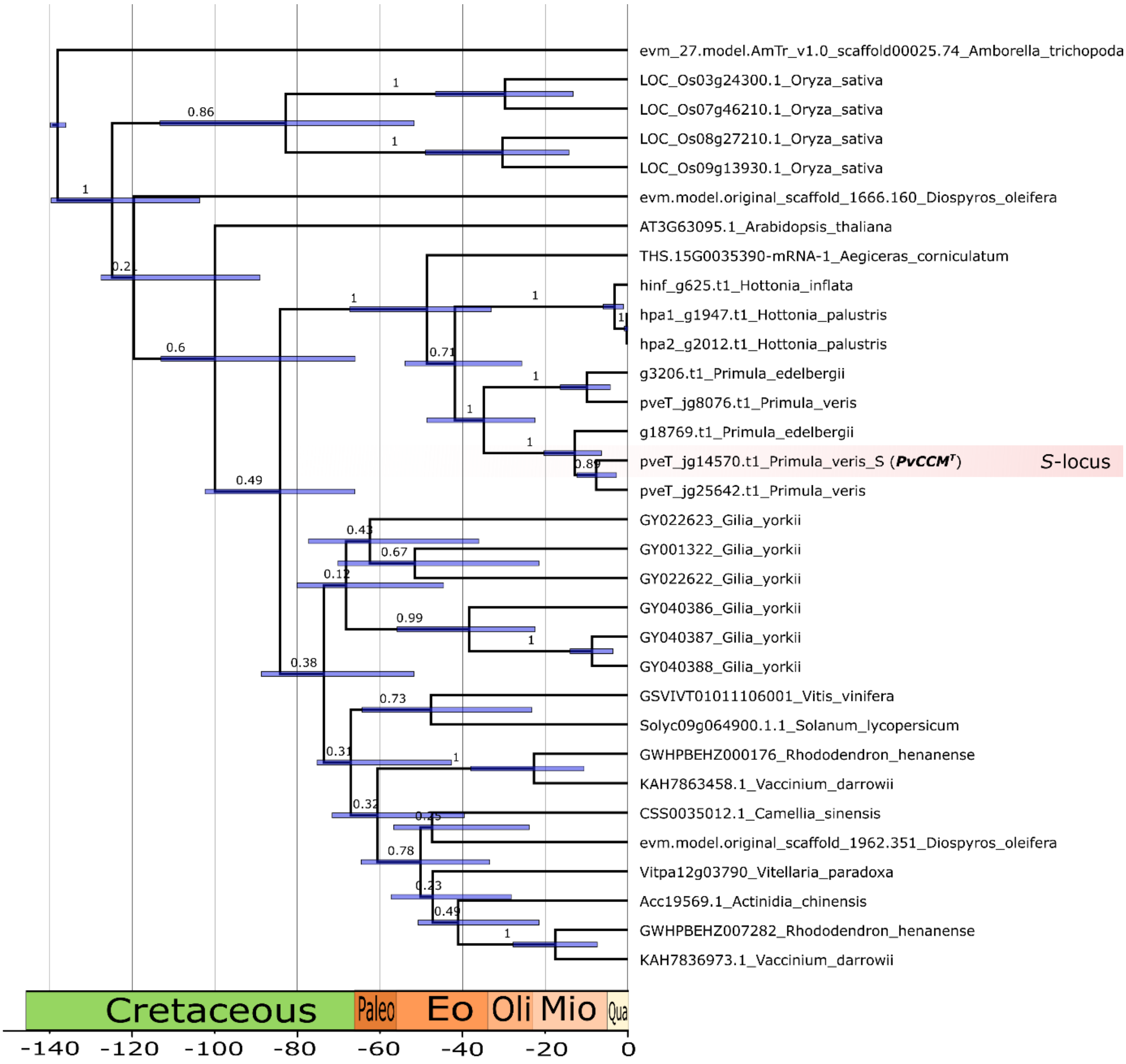

Fig. S34: Phylogeny of the *S*-gene *CCM*^{T} and close homologs.
Bayesian chronogram of *CCM*^{T} genes in selected genomes. Bottom scale bar indicates time before present in million years (My), with boxes indicating geological periods (pre-Cenozoic) or epochs (Cenozoic). Blue bars at nodes represent 95% Bayesian credibility intervals around age estimates. Branch labels represent posterior probabilities for the subtended clade.

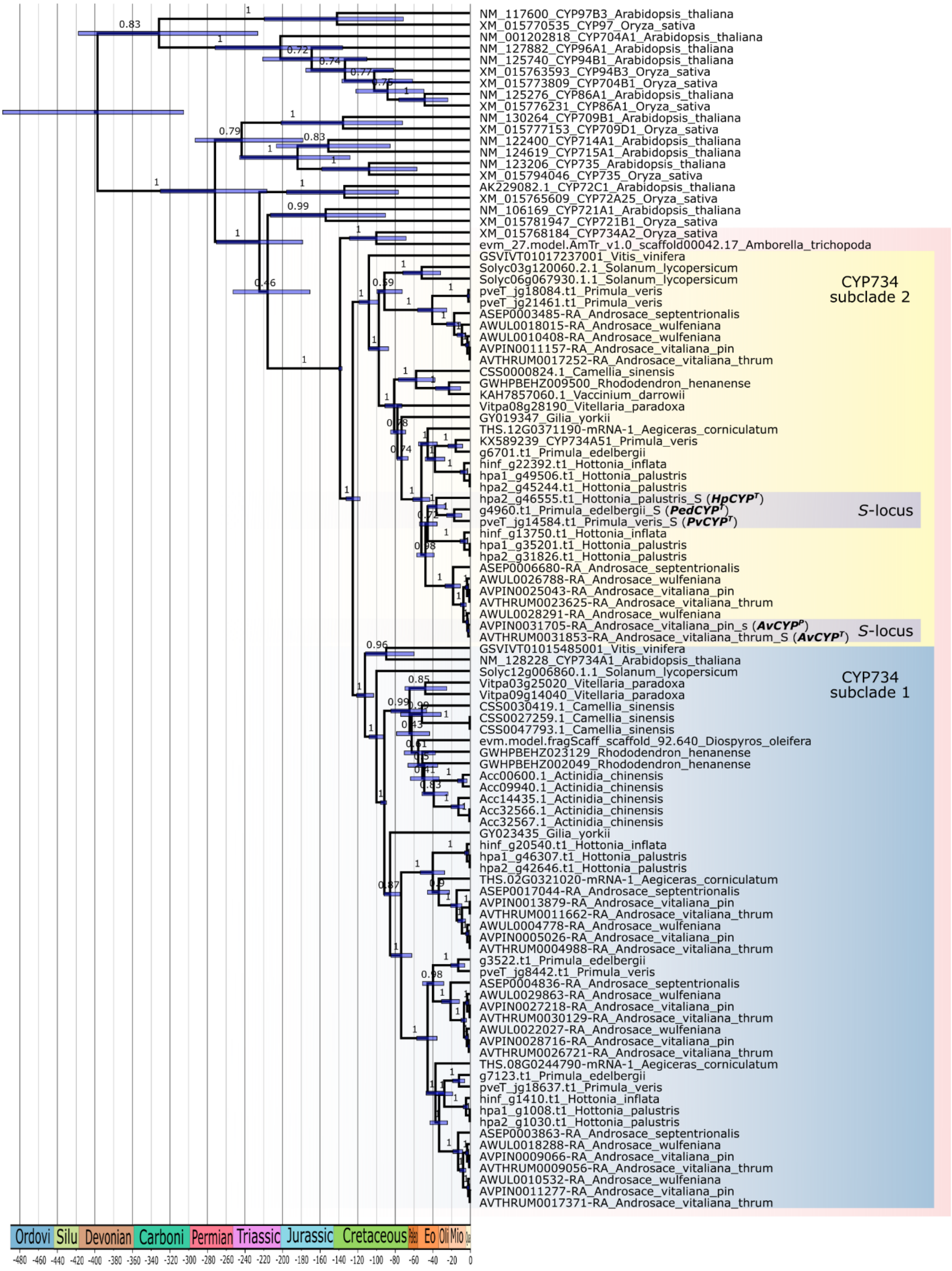

Fig. S35: Phylogeny of the *S*-gene CYP^T and close homologs.

Bayesian chronogram of CYP^T genes in selected genomes. Bottom scale bar indicates time before present in million years (My), with boxes indicating geological periods (pre-Cenozoic) or epochs (Cenozoic). Blue bars at nodes represent 95% Bayesian credibility intervals around age estimates. Branch labels represent posterior probabilities for the subtended clade.

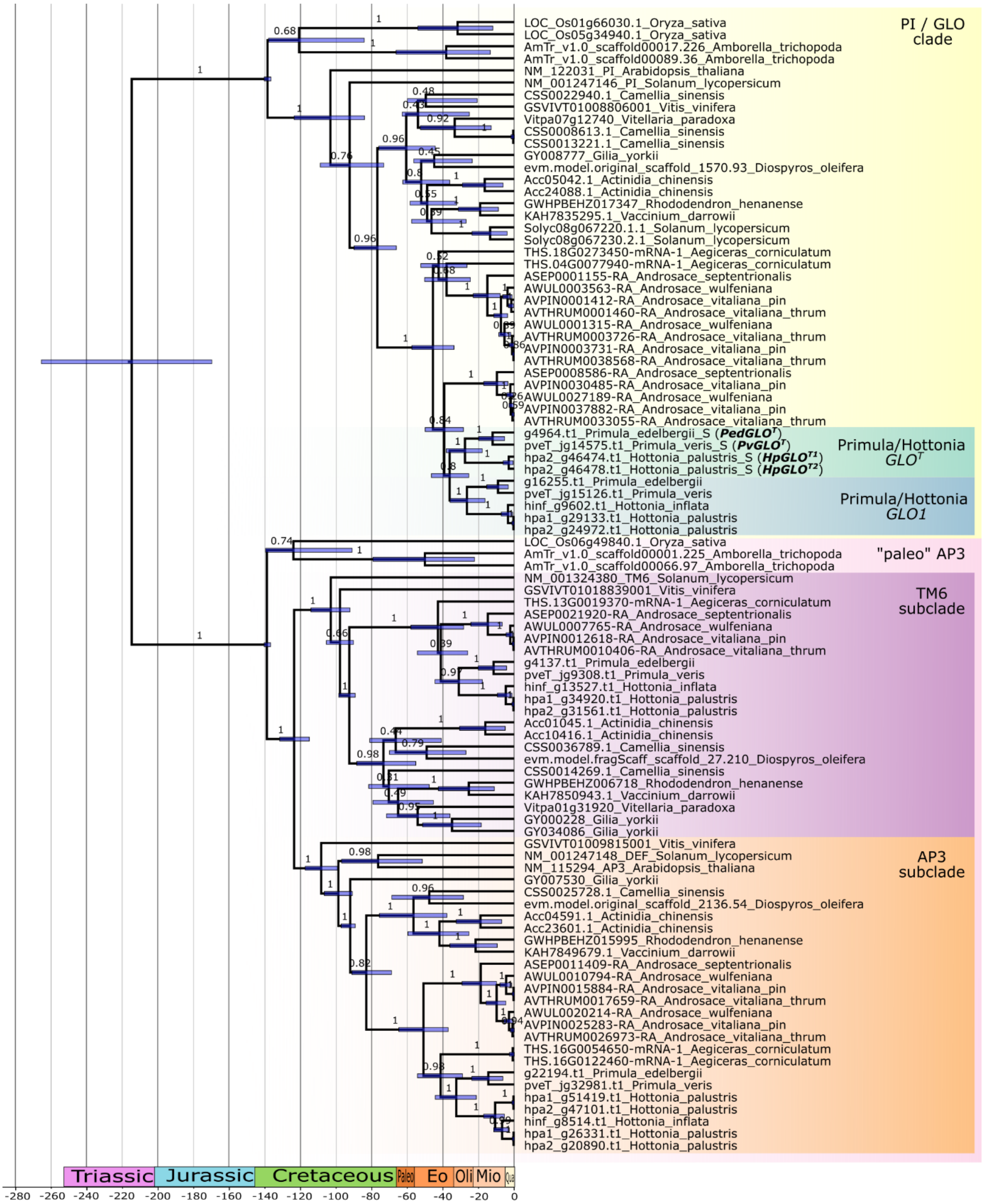

Fig. S36: Phylogeny of the *S*-gene *GLO*^{T} and close homologs.

Bayesian chronogram of *GLO*^{T} genes in selected genomes. Bottom scale bar indicates time before present in million years (My), with boxes indicating geological periods (pre-Cenozoic) or epochs (Cenozoic). Blue bars at nodes represent 95% Bayesian credibility intervals around age estimates. Branch labels represent posterior probabilities for the subtended clade.

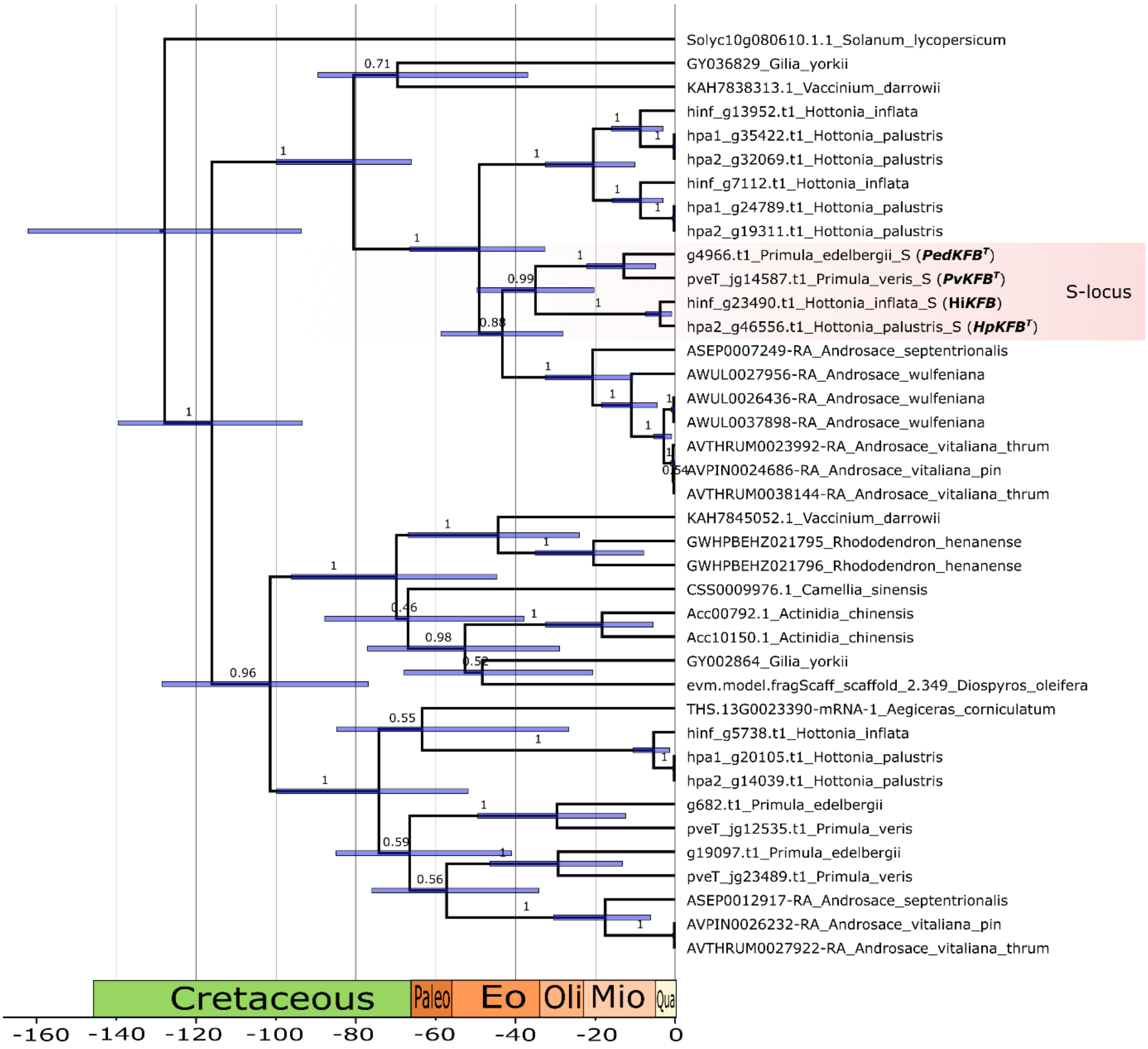

Fig. S37: Phylogeny of the *S*-gene *KFB*^{T} and close homologs.
Bayesian chronogram of *KFB*^{T} genes in selected genomes. Bottom scale bar indicates time before present in million years (My), with boxes indicating geological periods (pre-Cenozoic) or epochs (Cenozoic). Blue bars at nodes represent 95% Bayesian credibility intervals around age estimates. Branch labels represent posterior probabilities for the subtended clade.

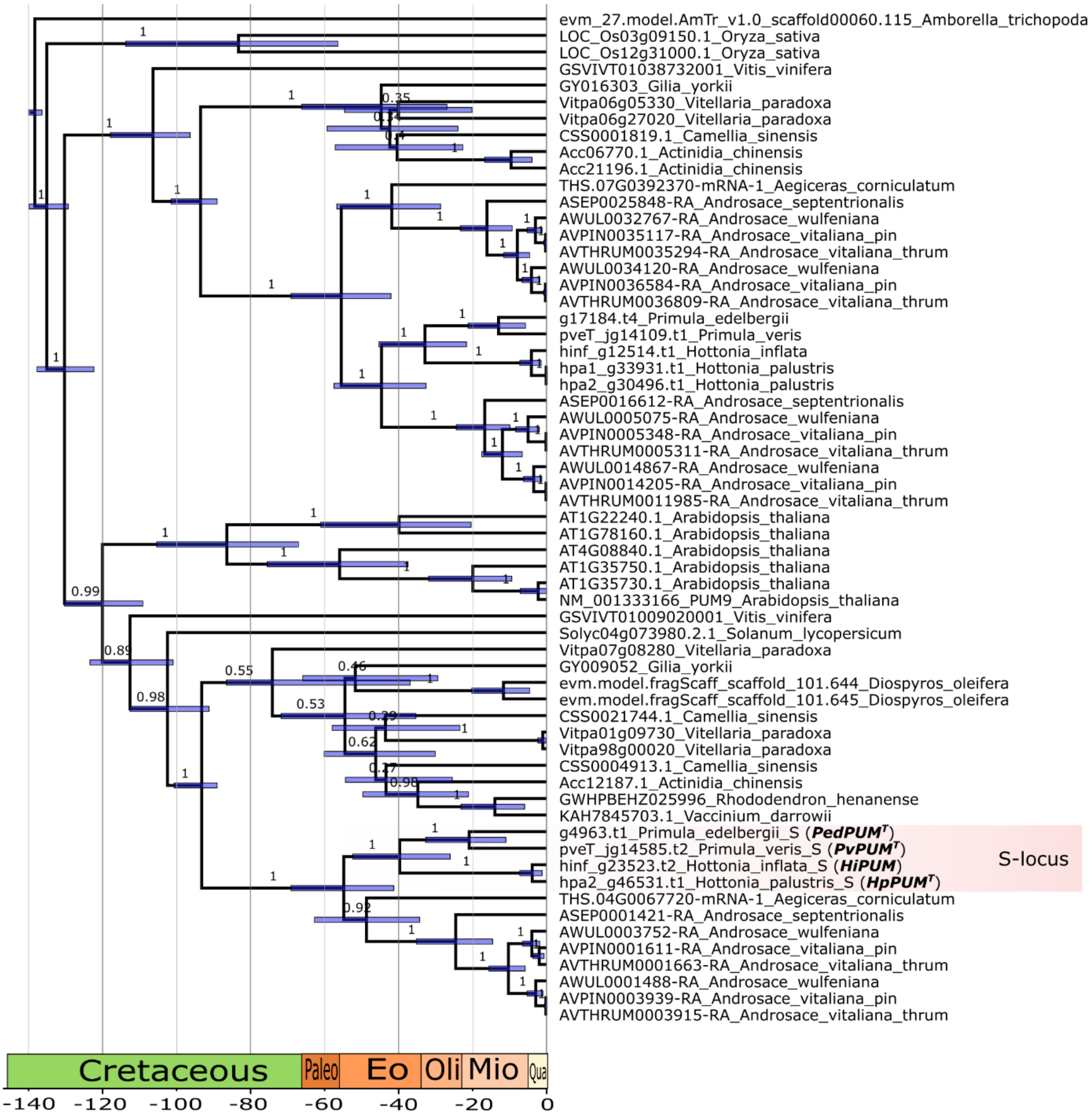

Fig. S38: Phylogeny of the *S*-gene *PUM*^{T} and close homologs.

Bayesian chronogram of *PUM*^{T} genes in selected genomes. Bottom scale bar indicates time before present in million years (My), with boxes indicating geological periods (pre-Cenozoic) or epochs (Cenozoic). Blue bars at nodes represent 95% Bayesian credibility intervals around age estimates. Branch labels represent posterior probabilities for the subtended clade.

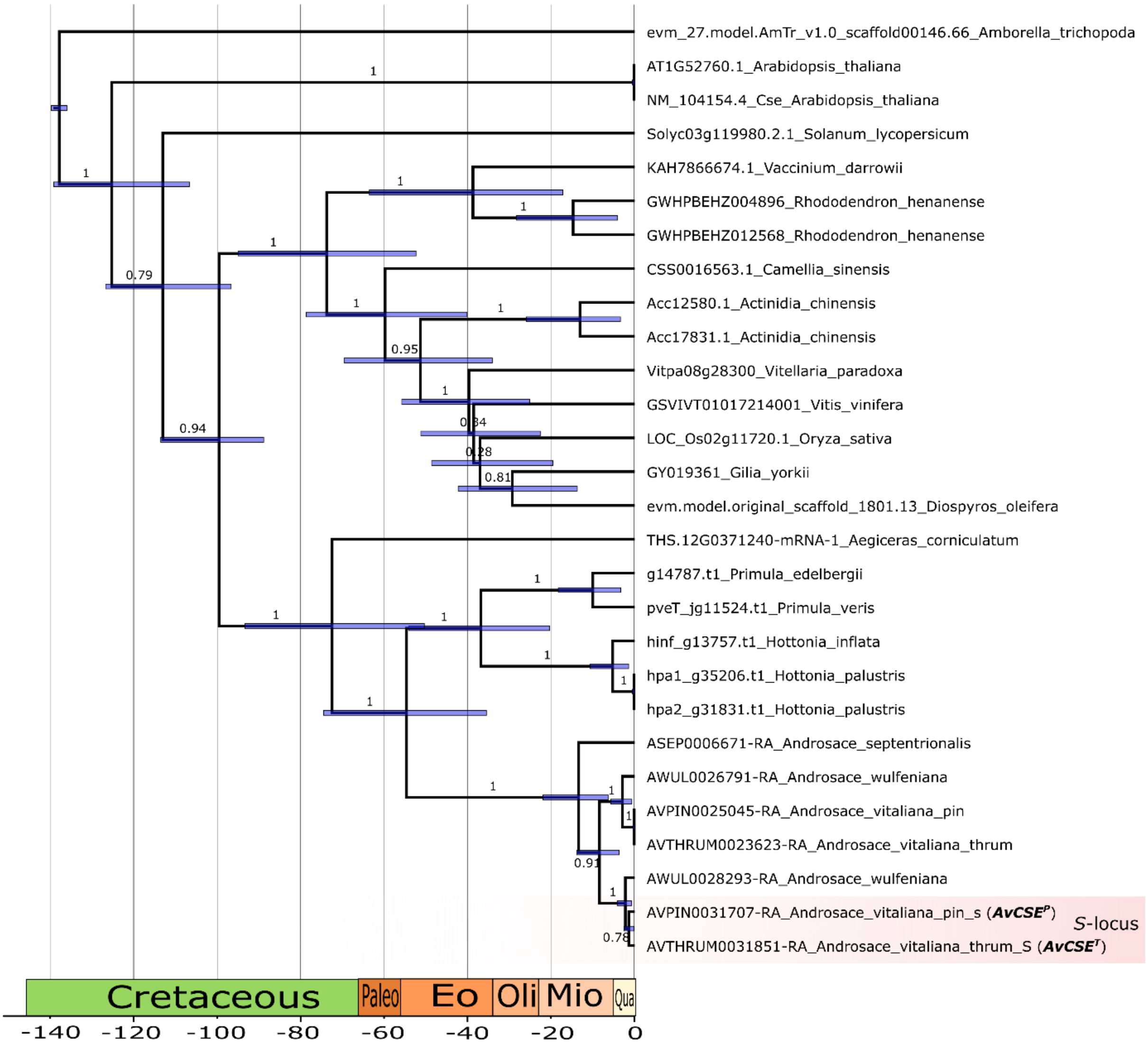

Fig. S39: Phylogeny of the *S*-gene *AvCSE*^{T} and close homologs.
Bayesian chronogram of *AvCSE*^{T} genes in selected genomes. Bottom scale bar indicates time before present in million years (My), with boxes indicating geological periods (pre-Cenozoic) or epochs (Cenozoic). Blue bars at nodes represented 95% Bayesian credibility intervals around age estimates. Branch labels represent posterior probabilities for the subtended clade.

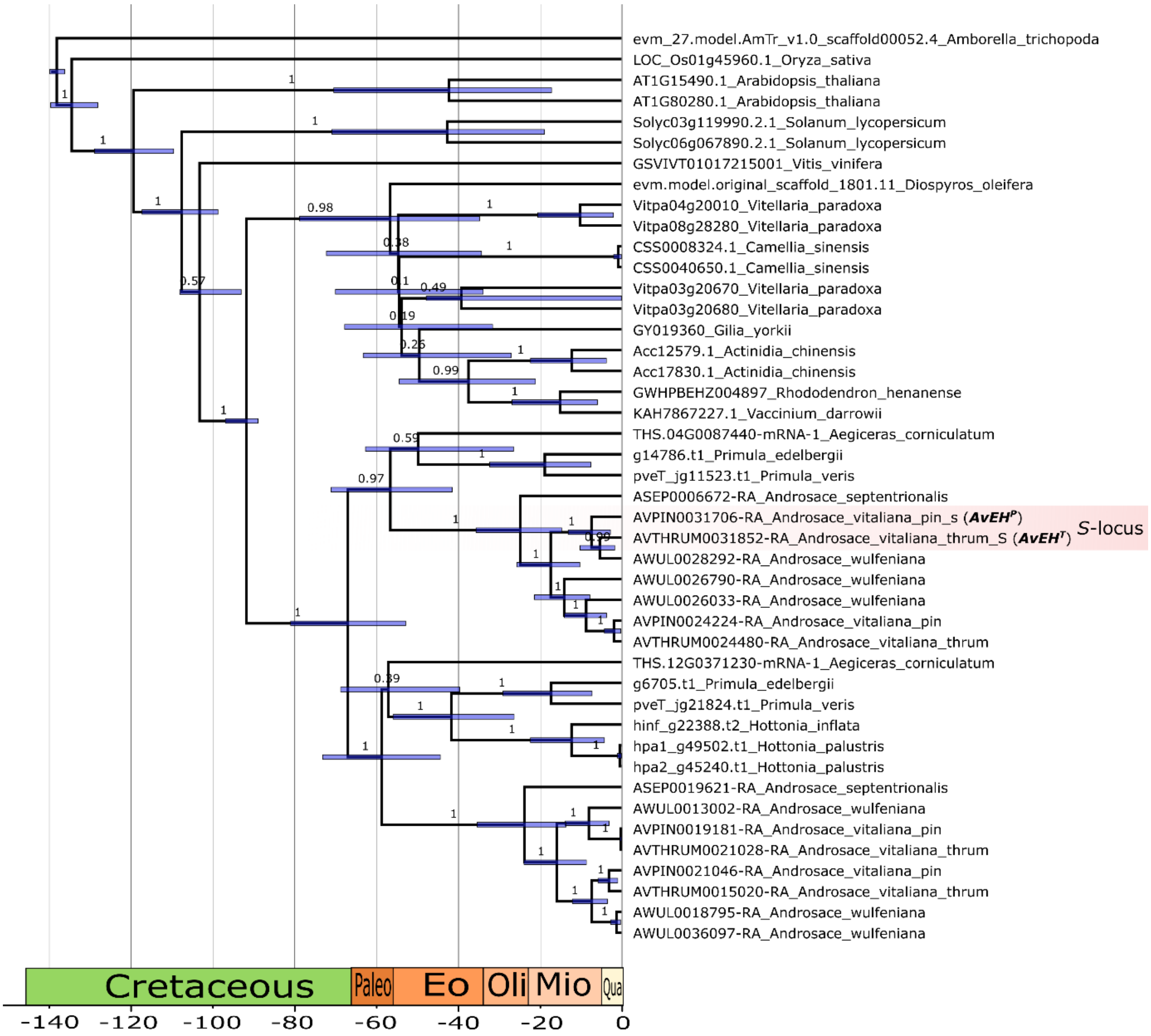

Fig. S40: Phylogeny of the *S*-gene $AvEH^T$ and close homologs.
Bayesian chronogram of $AvEH^T$ genes in selected genomes. Bottom scale bar indicates time before present in million years (My), with boxes indicating geological periods (pre-Cenozoic) or epochs (Cenozoic). Blue bars at nodes represented 95% Bayesian credibility intervals around age estimates. Branch labels represent posterior probabilities for the subtended clade.

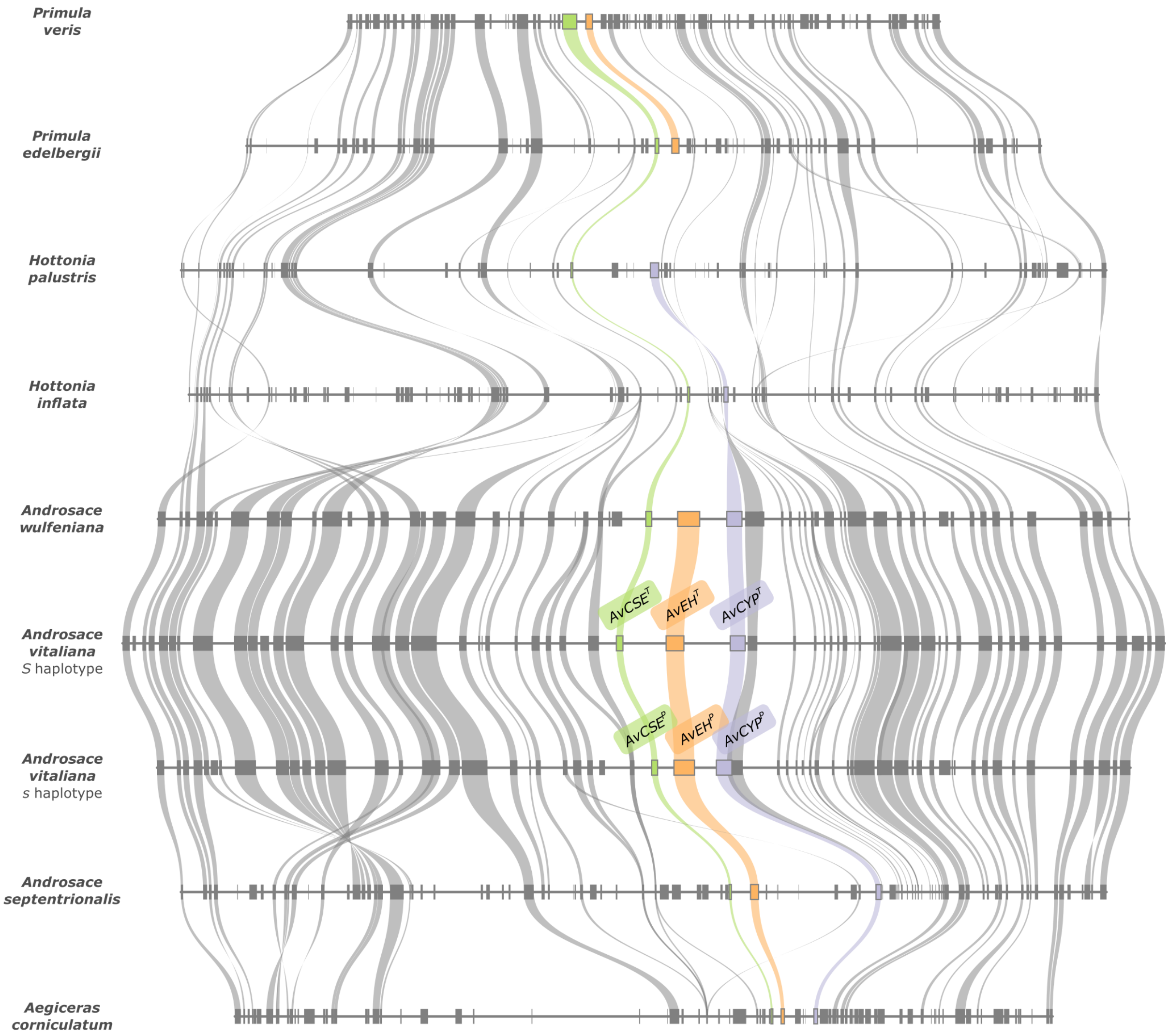

Fig. S41: The *Androsace vitaliana S*-genes were ancestrally colocalized.
Microsynteny plot across nine Primulaceae genome assemblies, showing that the *A. vitaliana S*-genes (*AvCSE*, *AvEH*, and *AvCYP*, colored in green, orange, and purple, respectively) are contained in a genomic region syntenic across Primulaceae. The orthologs of the three *A. vitaliana S*-genes are present in this region in all *Androsace* species and in the outgroup *Aegiceras corniculatum*, while *Primula* and *Hottonia* species lack orthologs of *AvCYP* and *AvEH*, respectively.

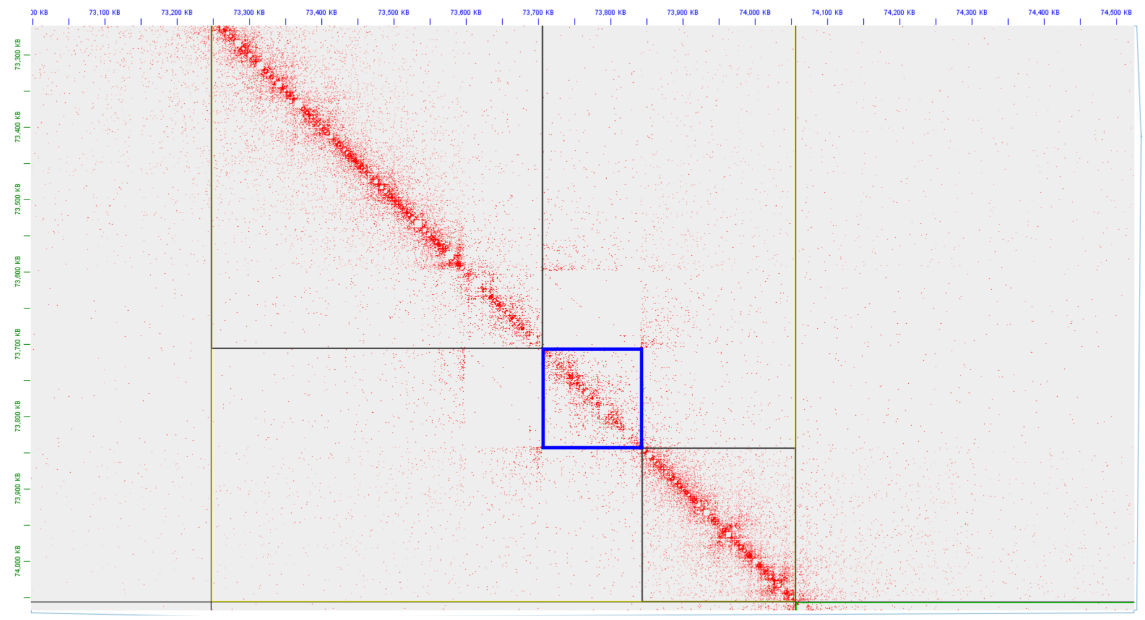

Fig. S42: The *Androsace vitaliana S*-locus is assembled in a single contig.
Hi-C contact map of the thrum assembly of *A. vitaliana* before scaffolding. Given the high sequence divergence between the *S*- and *s*-haplotypes, the *S*-locus was assembled in two different contigs. Specifically, contig “2035” (here highlighted by a blue box) contained the *S*-haplotype.

Data S1 (separate file)

All supplementary tables (Table S1-S21) are included in Data S1:

- Table S1: Flow-cytometry estimates of genome sizes.
- Table S2: Short-read DNA sequencing data.
- Table S3: Long-read DNA sequencing data.
- Table S4: Statistics on genome assemblies presented in the current study.
- Table S5: Identification of centromeric repeats using QuarTeT.
- Table S6: Identification of telomeric repeats using QuarTeT.
- Table S7: Short-read RNA sequencing data.
- Table S8: Functional annotation of *Androsace septentrionalis* genes.
- Table S9: Functional annotation of *Androsace vitaliana* genes (pin haplotype).
- Table S10: Functional annotation of *Androsace vitaliana* genes (thrum haplotype).
- Table S11: Functional annotation of *Androsace wulfeniana* genes.
- Table S12: Functional annotation of *Hottonia inflata* genes.
- Table S13: Functional annotation of *Hottonia palustris* genes (pin haplotype).
- Table S14: Functional annotation of *Hottonia palustris* genes (thrum haplotype).
- Table S15: Classification of genes in the *H. palustris s*-haplotype.
- Table S16: Classification of genes in the *H. palustris S*-haplotype.
- Table S17: Repetitive elements in distylous species of Primulaceae.
- Table S18: S-genes and their orthologs in Primulaceae.
- Table S19: Genes of *Primula* and *Androsace* species syntenic to the *H. plaustris S*-locus.
- Table S20: Orthogroups generated by OrthoFinder.
- Table S21: Genome assembly BioProjects.
